# The Role of Obesity, Type 2 Diabetes, and Metabolic Factors in Pancreatic Cancer: A Mendelian Randomization Study

**DOI:** 10.1093/jnci/djx012

**Published:** 2017-04-28

**Authors:** Robert Carreras-Torres, Mattias Johansson, Valerie Gaborieau, Philip C. Haycock, Kaitlin H. Wade, Caroline L. Relton, Richard M. Martin, George Davey Smith, Paul Brennan

**Affiliations:** * **Affiliations of authors:** Section of Genetics, International Agency for Research on Cancer (IARC), Lyon, France (RCT, MJ, VG, PB); MRC Integrative Epidemiology Unit, School of Social and Community Medicine, University of Bristol, Bristol, UK (PCH, KHW, CLR, RMM, GDS); National Institute for Health Research Biomedical Research Unit in Nutrition, Diet and Lifestyle at University Hospitals Bristol NHS Foundation Trust and the University of Bristol, Bristol, UK (RMM).

## Abstract

**Background:**

Risk factors for pancreatic cancer include a cluster of metabolic conditions such as obesity, hypertension, dyslipidemia, insulin resistance, and type 2 diabetes. Given that these risk factors are correlated, separating out causal from confounded effects is challenging. Mendelian randomization (MR), or the use of genetic instrumental variables, may facilitate the identification of the metabolic drivers of pancreatic cancer.

**Methods:**

We identified genetic instruments for obesity, body shape, dyslipidemia, insulin resistance, and type 2 diabetes in order to evaluate their causal role in pancreatic cancer etiology. These instruments were analyzed in relation to risk using a likelihood-based MR approach within a series of 7110 pancreatic cancer patients and 7264 control subjects using genome-wide data from the Pancreatic Cancer Cohort Consortium (PanScan) and the Pancreatic Cancer Case-Control Consortium (PanC4). Potential unknown pleiotropic effects were assessed using a weighted median approach and MR-Egger sensitivity analyses.

**Results:**

Results indicated a robust causal association of increasing body mass index (BMI) with pancreatic cancer risk (odds ratio [OR] = 1.34, 95% confidence interval [CI] = 1.09 to 1.65, for each standard deviation increase in BMI [4.6 kg/m^2^]). There was also evidence that genetically increased fasting insulin levels were causally associated with an increased risk of pancreatic cancer (OR = 1.66, 95% CI = 1.05 to 2.63, per SD [44.4 pmol/L]). Notably, no evidence of a causal relationship was observed for type 2 diabetes, nor for dyslipidemia. Sensitivity analyses did not indicate that pleiotropy was an important source of bias.

**Conclusions:**

Our results suggest a causal role of BMI and fasting insulin in pancreatic cancer etiology.

Pancreatic cancer is usually asymptomatic at early stages and presents at an advanced incurable stage with five-year survival rates of around 5% (1). Population-based screening for pancreatic cancer is not currently an option because of the lack of an accurate screening biomarker (2). The identification of risk factors for primary prevention is therefore of major interest as a method to reduce the incidence and consequences of the disease.

Tobacco exposure and obesity are the only modifiable factors with convincing evidence to be considered causal risk factors for pancreatic cancer (3,4). A dose-response relationship has been observed with cigarette smoking (5,6). Body mass index (BMI) is also associated with a modest increase in risk, estimated to be between 10% and 50% per five-unit BMI (kg/m^2^) increment (7–9). Other related anthropometric and metabolic factors have also been reported with modest effect sizes, including height and waist-to-hip ratio (8,10). Additionally, obesity is linked to a cascade of metabolic conditions, including hypercholesterolemia, hyperglycemia, insulin resistance, and type 2 diabetes. Cholesterol intake, higher glucose levels, hyperinsulinemia, and type 2 diabetes status have all been identified as potential pancreatic cancer risk factors (11–15). The clustering of these conditions is often referred to as metabolic syndrome, although the specific parameters that lead to an increase in pancreatic cancer risk are unclear (6,16).

Mendelian randomization (MR) is an analytical approach based on instrumental variable analysis and uses gene variants associated with the risk factors of interest as unconfounded markers of those factors (17). Important assumptions in instrumental variable analysis are that the chosen genetic variants are associated with the exposure of interest, they are not associated with any confounders, and they are not associated with the cancer outcome via any pathway other than through the exposure of interest (known as genetic pleiotropy) (18). Genetic variants satisfying these three assumptions divide a study population into subgroups that are analogous to treatment arms in a randomized controlled trial, in that they differ systematically with respect to the exposure of interest, but not with respect to confounders. If all the instrumental variable assumptions are met, an association between the genetic variant and the outcome implies that the risk factor of interest has a causal effect on the outcome (19).

In this study, we used genetic variation associated with obesity and other metabolic traits as unconfounded instruments to investigate the causal relationship between these metabolic exposures and pancreatic cancer in case and control individuals of similar European origin. Genetic proxies for modifiable exposures were identified in several large genome-wide association studies (GWAS) of the risk factors of interest, and these genetic proxies were tested for association in a total of 7110 pancreatic cancer cases and 7264 controls obtained from the Pancreatic Cancer Cohort Consortium (PanScan) and Pancreatic Cancer Case-Control Consortium (PanC4) (20–22) via dbGaP (23). We applied two-sample MR, an approach that combines summary statistics on the genetic variant to exposure and genetic variant to outcome associations from different samples (24,25) and provides estimates of the strength of the association between exposure and outcome.

## Methods

### Genetic Instruments for Putative Risk Factors

Genetic instruments for each risk factor were single-nucleotide polymorphisms (SNPs) independently (linkage disequilibrium [LD] *R*^2^ measure < 0.2) associated with the trait at a genome-wide level (*P* < 5x10^−8^) identified in the most recent and largest GWAS results on that trait from samples of European ethnicity. Results from the Genetic Investigation of ANthropometric Traits (GIANT) consortium were used to identify genetic proxies for height (26), BMI (27), and waist-to-hip ratio (28). High-density and low-density lipoprotein cholesterol (HDL and LDL, respectively), total cholesterol, and triglycerides were selected as lipid profile components. Genetic loci influencing bloodstream levels of these lipids were identified from GWAS data provided by the Global Lipids Genetic Consortium (GLGC) (29). Similarly, data from the Meta-Analysis of Glucose and Insulin related traits Consortium (MAGIC) were used to identify genetic loci for glycemic traits including fasting glucose, fasting insulin, and two-hour-postchallenge glucose (30). Finally, genetic instruments for type 2 diabetes were identified from a recent genetic fine mapping study (31,32). For each identified SNP, the reported effect allele size was for the allele associated with an increase in the trait and expressed in one standard deviation of the trait per allele (β_GP_), along with the standard error. For studies in which the genetic effects were not originally reported in SD units of the trait, they were recalibrated according to the mean SD and weighted for sample size across the different case-control samples. SNPs with ambiguous strand codification (A/T or C/G) were replaced by SNPs in tight genetic linkage (LD *R*^2^ > 0.8) using the SNP Annotation and Proxy Search (SNAP; https://www.broadinstitute.org/mpg/snap/ldsearch.php) or removed from the analyses. The number of identified SNPs and proportion of variance explained for each risk factor are detailed in Table 1.

**Table 1.** Potential risk factors for pancreatic cancer, number of identified instrumental SNPs, phenotype distribution (mean and SD) in the discovery sample, and percentage of the phenotype variance explained by the instruments*

Phenotype	No. of SNPs	Mean ± SD	Units	Variance explained, %	Consortium	Reference No.
Height	567	169.9 ± 6.9	cm	16.0	GIANT	26
Body mass index	96	27.0 ± 4.6	kg/m^2^	2.7	GIANT	27
Waist-to-hip ratio	34	1.1 ± 0.1	cm/cm	1.4	GIANT	28
High-density cholesterol	70	53.3 ± 15.5	mg/dL	13.7	GLGC	29
Low-density cholesterol	54	133.6 ± 38	mg/dL	14.6	GLGC	29
Total cholesterol	72	213.28 ± 42.6	mg/dL	15.0	GLGC	29
Triglycerides	40	140.85 ± 87.8	mg/dL	11.7	GLGC	29
Fasting glucose	37	5.2 ± 0.8	mmol/L	4.8	MAGIC	30
Fasting insulin	17	56.9 ± 44.4	pmol/L	1.2	MAGIC	30
2-h-postchallenge glucose	9	5.6 ± 1.7	mmol/L	1.7	MAGIC	30
Type 2 diabetes	44			5.7	DIAGRAM	31,32

* DIAGRAM = DIAbetes Genetics Replication And Meta-analysis; GIANT = Genetic Investigation of ANthropometric Traits; GLGC = Global Lipids Genetic Consortium; MAGIC =Meta-Analysis of Glucose and Insulin related traits Consortium; SNP = single-nucleotide polymorphism.

### Pancreatic Cancer Samples and Meta-analysis

GWAS data from pancreatic cancer samples were obtained from the PanScan (12 studies) and PanC4 (10 studies) consortia through the National Center for Biotechnology Information database of Genotypes and Phenotypes (dbGaP; Study Accession: phs000206.v3.p2 and phs000648.v1.p1; project reference #9314) (23) and were originally published in three different sets called PanScan I (1788 cases and 1769 controls), PanScan II (1696 cases and 1563 controls), and PanC4 (3626 PanC4 cases and 3932 controls) (20–22). These samples comprised 7638 cases and 7364 controls of European origin and were originally genotyped using Illuminia HumanHap550, Human610-Quad, and HumanOmniExpressExome-8v1 arrays, respectively (Illumina Inc. San Diego, CA). Detailed sample characteristics can be observed in Supplementary Table 1 (available online). Initial quality control steps and analyses were performed within each publication set. After removing duplicates, related samples, samples with sex discrepancy, and population outliers, 7110 cases and 7264 controls remained. Genotype imputation was performed using the Michigan Imputation Server (33). Genotypes were prephased using SHAPEIT v2 (34) and imputed with Minimach v3 (35) using the Haplotype Reference Consortium panel (36). After imputation, SNPs with imputation quality (*R*^2^) lower than 0.7 were removed from the data sets. Association statistics on pancreatic cancer risk were obtained adjusting for age, sex, and statistically significant eigenvectors for population stratification using R software. Association statistics were also obtained for sex strata. Results from each set were then combined using a fixed-effects inverse-SE approach implemented in METAL (37), obtaining the pancreatic cancer risk estimates (β_GD_) and SE. For each SNP used as an instrument in this report, SNP to phenotype effect (β_GP_) and SNP to disease effect (β_GD_) can be observed in Supplementary Table 2 (available online).

### Statistical Analyses

#### Power Assessment

Power calculations on MR analyses were performed for four genetic instruments, based on the number of cases and controls of the pooled sample (38). The four genetic instruments corresponded to different explained proportions of phenotype variance (1.5% representing waist-to-hip ratio, fasting insulin, and glucose at two hours postchallenge; 2.7% for BMI; 5% representing fasting glucose and type 2 diabetes; and 10% as a lower threshold of lipid parameters) (Table 1). Power calculations can be observed in Supplementary Figure 1 (available online). Additionally, we assessed our power to validate previously observed risk increase from potential risk parameters analyzed in this study. Our sample had a high power to validate previously observed risk increases for BMI (86.3% of power for a risk increase of 39% per SD increase) and type 2 diabetes (99.5% for an increase of 40%), although it was only modestly powered for height (57.7% for an increase of 10%) and fasting glucose (65.5% for a 21% risk increase), and underpowered for a risk increase of 19% from waist-to-hip ratio (21.2%) (Supplementary Table 3, available online).

#### Mendelian Randomization Analyses

The causal effect on pancreatic cancer was estimated using a likelihood-based approach (24) for the pooled sample, after stratifying by publication set (PanScan I, PanScan II, and PanC4) and for sex. The MR likelihood-based approach is considered the most accurate method to estimate causal effects when there is a continuous log-linear association between risk factor and disease risk. The resulting odds ratios (ORs) and 95% confidence intervals (CIs) provided an estimate of relative risk caused by each SD increase in the trait (Table 1). We also investigated the between-study and between-sex heterogeneity of causal effects, estimating the percentage of variance that is attributable to study or sex heterogeneity (I^2^ statistic), the Q statistic for heterogeneity, and its *P* value (*P*_Heterogeneity_), assuming a fixed-effect model of 2 degrees of freedom for study heterogeneity and 1 degree of freedom for sex heterogeneity. This was done using the *meta* R package (R project). To evaluate the potential effect of pleiotropy on the likelihood risk estimates, we used different complementary approaches. Because genetic variants for metabolic factors can be confounded by BMI effect, a likelihood-based approach was performed for the nonobesity exposures excluding the genetic variants known to be also robustly associated with BMI. Additionally, two approaches, namely the weighted median estimation (39) and the MR-Egger approach (40), were performed on the initial set of genetic variants to detect bias due to pleiotropy from unknown origin. The weighted median estimator is the median of a distribution in which Wald ratio estimates have been ordered and represent percentiles of this distribution (39), which is less sensitive to the effect of pleiotropic variants behaving as outliers. On the other hand, the MR-Egger approach performs a weighted linear regression of the SNP to disease effects (β_GD_) on the SNP to phenotype effects (β_GP_). In this test, the analyses of the regression intercept detects an overall directional pleiotropic contribution of weak instrumental SNPs on the risk estimate (assuming that any pleiotropic contribution biasing the risk estimation is acting in the same direction) (40). For each potential risk factor, a scatter plot of the SNP risk increase (exp(β_GD_/β_GP_)) against the strength of instrumental SNPs (β_GP_/SE_GD_) was constructed, providing a funnel plot for visual assessment of asymmetry of instrumental causal estimates. These plots were generated using the *ggplot2* R package (R Project).

Finally, in order to explore the causal effect of mechanistic pathways in which genetic instruments clustered, for those risk factors with more than 50 (ie, height, BMI, and lipid parameters), the genetic instrument set was divided in subsets according to mechanistic pathways as described in the original GWAS study (with a minimum of five SNPs in each subset). These genetic instrument subsets were subsequently tested for their association with pancreatic cancer using the MR likelihood-based approach.

All statistical tests were two-sided, a *P* value of less than .05 was considered statistically significant, and a Bonferroni correction for multiple testing was applied for mechanistic pathways tests.

## Results

### MR Likelihood-Based Results

**Figure 1.**
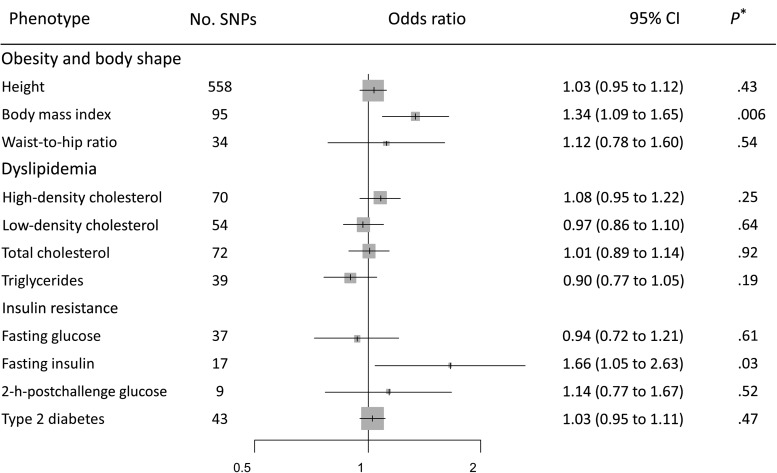
Forest plot of risk increase on pancreatic cancer for each standard deviation increase in the exposure. *Likelihood-based Mendelian randomization test. *P* values are two-sided. CI = confidence interval; OR = odds ratio.

**Figure 2.**
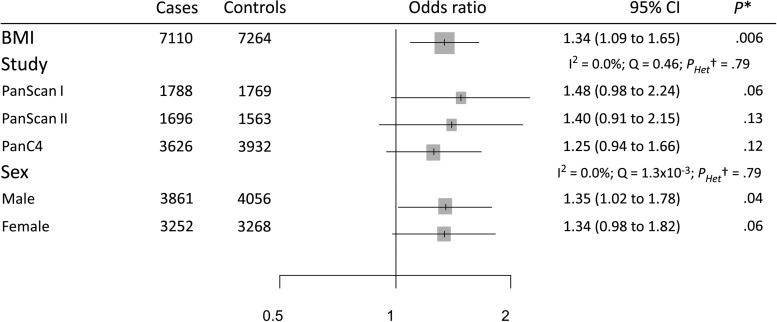
Forest plot of risk increase on pancreatic cancer for each standard deviation increase in body mass index stratified by publication sets and sex. *Likelihood-based Mendelian randomization test. *P* values are two-sided. †Heterogeneity Q test. *P*_heterogeneity_ values are two-sided. BMI = body mass index; CI = confidence interval; I^2^ = index of between-strata heterogeneity; OR = odds ratio; Q = statistic for between-strata heterogeneity.

**Figure 3.**
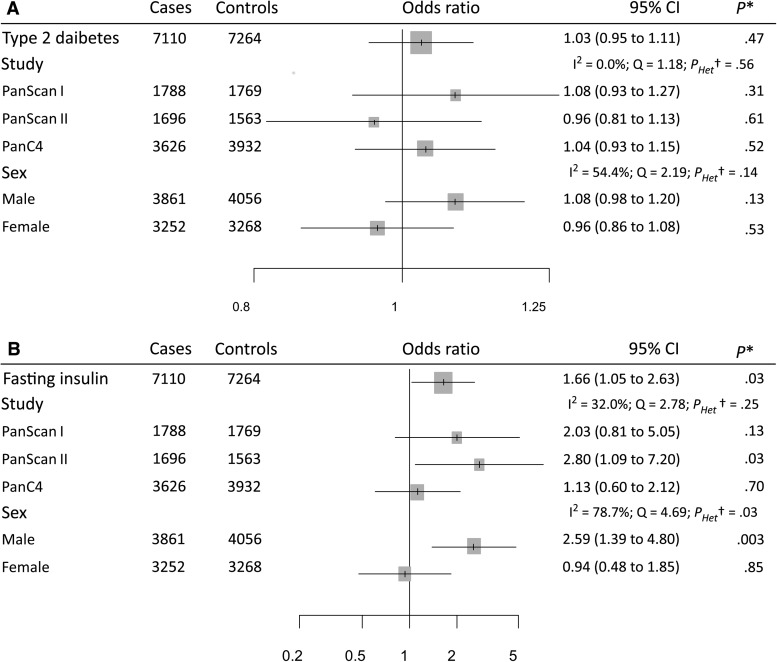
Forest plots of risk increase on pancreatic cancer for type 2 diabetes status **(A)** and for each standard deviation increase in fasting insulin levels **(B)**, stratified by publication sets and sex. *Likelihood-based Mendelian randomization test. *P* values are two-sided. †Heterogeneity Q test. *P*_heterogeneity_ values are two-sided. CI = confidence interval; I^2^ = index of between-strata heterogeneity; OR = odds ratio; Q = statistic for between-strata heterogeneity.

The genetic instrument for BMI comprising 95 instrumental SNPs indicated that the effect of each SD increase in BMI (4.6 kg/m^2^) increased pancreatic cancer risk (OR = 1.34, 95% CI = 1.09 to 1.65) (Figure 1). Stratified analyses by publication set and sex suggested consistent odds ratio estimates (OR ranging from 1.25 to 1.48, *P*_Heterogeneity_ > .79) (Figure 2). The genetic instrument for height (558 SNPs) did not indicate any causal association with risk (OR = 1.03, 95% CI = 0.95 to 1.12), nor for waist-to-hip ratio (34 SNPs; OR = 1.12, 95% CI = 0.78 to 1.60) (Figure 1; Supplementary Figure 2, A and B, available online, for the stratified analyses). Instruments for the lipid traits, including HDL, LDL, total cholesterol, and triglycerides, did not indicate an effect on overall risk of pancreatic cancer (Figure 1). Heterogeneity was not observed in the subgroup analyses (see, for instance, Supplementary Figure 2, C and D, available online, for stratified analyses on HDL and triglycerides, respectively). Four potential risk factors related to diabetes were evaluated, including type 2 diabetes, fasting glucose, fasting insulin, and two-hour-postchallenge glucose (Table 1). The genetic instrument for type 2 diabetes status was not associated with pancreatic cancer risk (OR = 1.03, 95% CI = 0.95 to 1.11) (Figure 1), although results by sex indicated a potential role among men (OR =  1.08, 95% CI =  0.98 to 1.20) but not for women (OR = 0.96, 95% CI = 0.86 to 1.08) (Figure 3A). In contrast, each SD increase in fasting insulin was associated with an increased risk of pancreatic cancer (OR = 1.66, 95% CI = 1.05 to 2.63) (Figure 1), with little evidence for between-study heterogeneity (Figure 3B). Conversely, the effect of fasting insulin appeared to differ by sex, the odds ratio estimate being 2.59 in men (95% CI = 1.39 to 4.80) and 0.94 in women (95% CI = 0.48 to 1.85; I^2^ = 78.7%, *P*_Heterogeneity_ = .03) (Figure 3B). Finally, there was no evidence that the genetic instruments for the glycemic traits were associated with pancreatic cancer risk (Figure 1; Supplementary Figures 2, E and F, available online, for the stratified analyses).

### Likelihood-Based Approach Excluding SNPs Robustly Associated With BMI

After removing BMI SNPs, four SNPs were dropped in analyses for type 2 diabetes, three for HDL and fasting insulin, two for two-hour-postchallenge glucose, and one for LDL, total cholesterol, triglycerides, and fasting glucose. This analysis resulted in odds ratio estimates that were similar to the initial estimates (Supplementary Table 4, available online), although the effect of fasting insulin was attenuated (OR = 1.26, 95% CI = 0.73 to 2.16). However, in the analyses stratified by sex, the fasting insulin effect on pancreatic cancer remained increased in men (OR = 2.27, 95% CI = 1.09 to 4.71) but not in women (OR = 0.64, 95% CI = 0.29 to 1.42).

### Weighted Median MR Results

Using the weighted median MR approach, the corresponding estimation of the risk increase was also increased for BMI (OR = 1.70, 95% CI = 1.22 to 2.36) and fasting insulin (OR = 1.94, 95% CI = 1.02 to 3.68) (Supplementary Table 5, available online), indicating that the original likelihood-based results were not due to a small subgroup of variants with a strong pleiotropic effect.

### MR-Egger Test

Finally, the analysis of the intercept in the MR-Egger test (providing an estimate of directional pleiotropy) for the different instruments suggested that directional pleiotropy was not an important phenomenon for the observed associations with BMI or fasting insulin. There was some evidence of directional pleiotropy on the overall risk estimation for triglycerides (intercept estimate = 0.02, 95% CI = 0.01 to 0.04) (Supplementary Table 5, available online). MR-Egger regression suggested an inverse association of triglycerides with risk of pancreatic cancer (OR = 0.63, 95% CI = 0.48 to 0.83; for an increase of 87.8 mg/dL) (Supplementary Table 5, available online).

**Figure 4.**
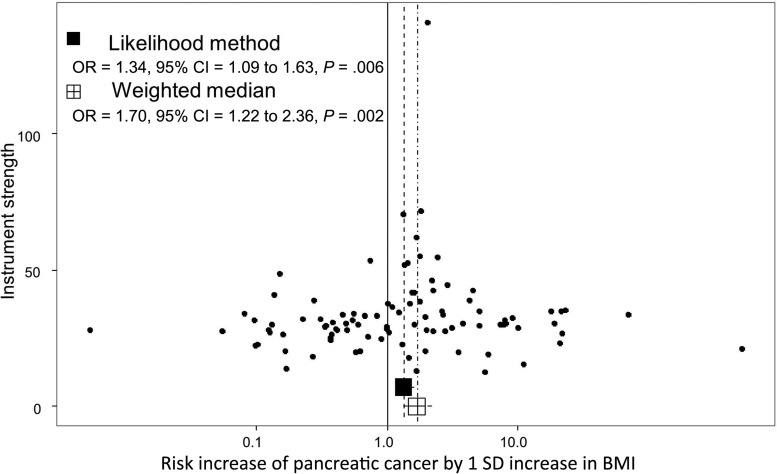
Funnel plot of risk estimates of body mass index (BMI)–instrumental single-nucleotide polymorphisms (SNPs) on pancreatic cancer against instrumental strength. Instrumental strength is SNP to pancreatic cancer effect corrected by SNP to BMI standard error of the effect. X-axis is in logarithmic scale. *P* values are two-sided, Mendelian randomization test. BMI = Body mass index; CI = confidence interval; OR = odds ratio.

**Figure 5.**
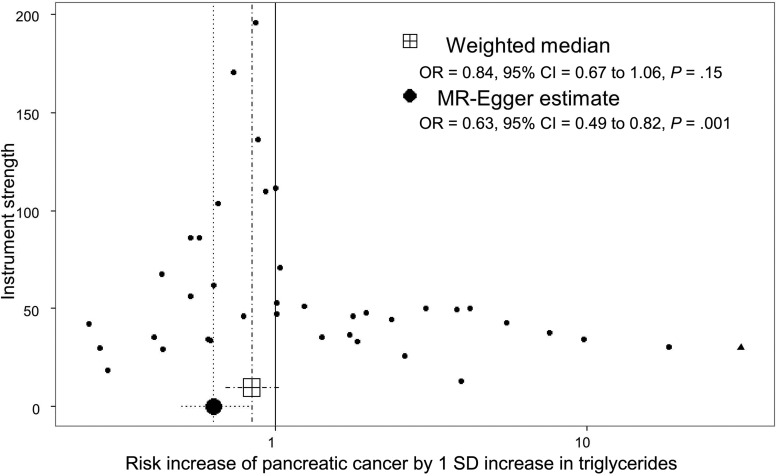
Funnel plot of risk estimates of triglyceride–instrumental single-nucleotide polymorphisms (SNPs) on pancreatic cancer against instrumental strength. Instrumental strength is SNP to pancreatic cancer effect corrected by SNP to triglycerides standard error of the effect. X-axis is in logarithmic scale. SNPs also robustly associated with BMI (1 SNPs) are depicted as triangles. *P* values are two-sided, Mendelian randomization test. CI = confidence interval; OR = odds ratio.

**Figure 6.**
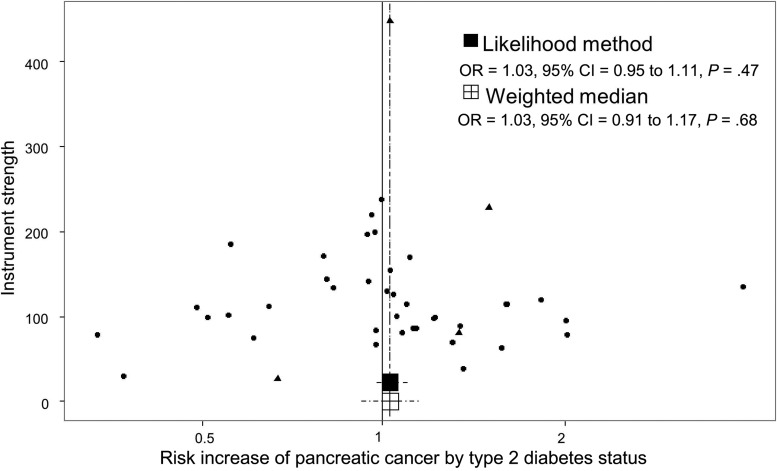
Funnel plot of risk estimates of type 2 diabetes–instrumental single-nucleotide polymorphisms (SNPs) on pancreatic cancer against instrumental strength. Instrumental strength is SNP to pancreatic cancer effect corrected by SNP to type 2 diabetes standard error of the effect. X-axis is in logarithmic scale. SNPs also robustly associated with BMI (4 SNPs) are depicted as triangles. *P* values are two-sided, Mendelian randomization test. CI = confidence interval; OR = odds ratio.

**Figure 7.**
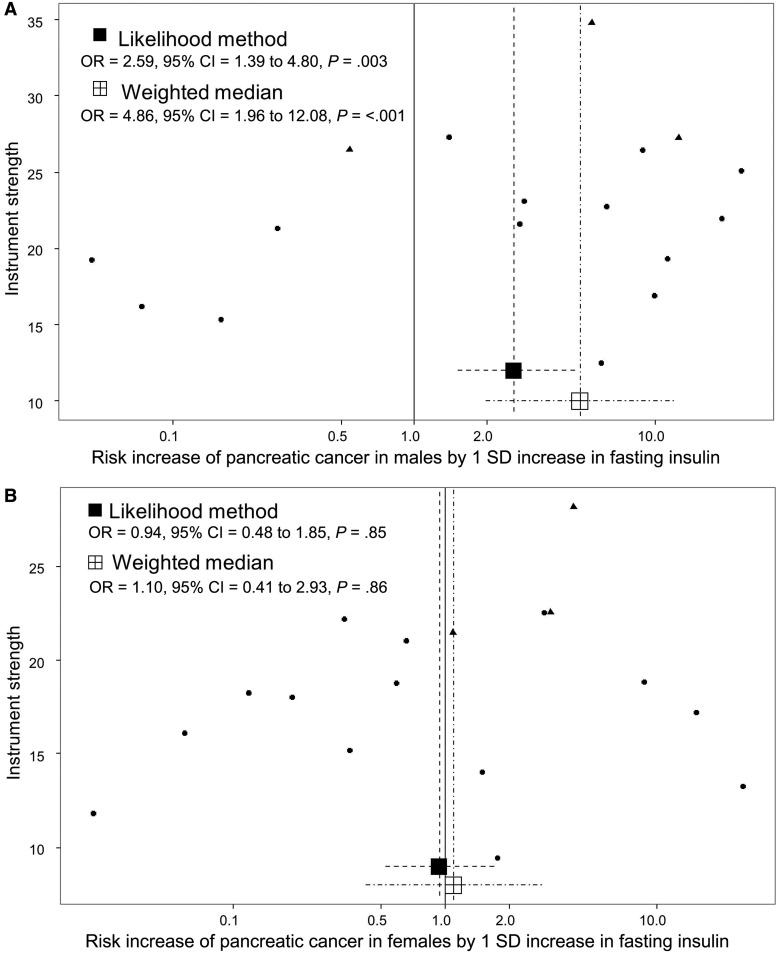
Funnel plots of risk estimates of fasting insulin–instrumental single-nucleotide polymorphisms (SNPs) on pancreatic cancer in male **(A)** and female **(B)** against instrumental strength. Instrumental strength is SNP to pancreatic cancer effect corrected by SNP to fasting insulin standard error of the effect. X-axis is in logarithmic scale. SNPs also robustly associated with BMI (three SNPs) are depicted as triangles. *P* values are two-sided, Mendelian randomization test. CI = confidence interval; OR = odds ratio.

The distribution of risk estimates of BMI SNPs along with the likelihood-based and weighted median risk causal effects for BMI can be observed in the funnel plot of Figure 4. In this figure, the overall symmetrical distribution suggests a lack of pleiotropy on the BMI causal estimates. In contrast, the corresponding funnel plot for triglycerides (Figure 5) showed evidence of a pleiotropic effect of some instrumental SNPs for triglycerides on the initial risk estimate detected in the MR-Egger analysis (asymmetry of instrumental risk estimations toward positive effects). For type 2 diabetes, the distribution of SNP risk estimates showed symmetry around unity (Figure 6). Finally, sex discrepancies on causal effects for fasting insulin on pancreatic cancer can be observed in Figure 7, A and B, for men and women, respectively. Funnel plots for the other tested parameters were included in Supplementary Figure 3 (available online).

### MR Likelihood-Based Results for Mechanistic Pathway Components of Risk Factors

Genetic instruments clustered in 127 different mechanistic pathways for height, 19 for BMI, three for HDL and LDL, four for total cholesterol, and one for triglycerides. No mechanistic pathway component appeared associated with pancreatic cancer risk after Bonferroni correction for multiple testing (*P* < 3.2 x10^-4^; lowest *P* = .02) (Supplementary Table 6, available online).

## Discussion

We have used data from large GWA studies on pancreatic cancer to evaluate the causal relevance of metabolic risk factors within an MR framework. Our results support higher BMI as a causal risk factor of pancreatic cancer, as well a potential causal role of higher insulin, in particular among men. Conversely, our results provided little support for a causal role of type 2 diabetes or dyslipidemia in pancreatic cancer.

For BMI, we observed a 34% increase in pancreatic cancer risk per SD increase (4.6 kg/m^2^). This is similar to the associations reported in observational studies using measured BMI (7–9). In the case of height and waist-to-hip ratio, our analyses did not detect higher risk effects than those previously observed in the literature, for which our sample was underpowered. Thus, our analyses of the relationship between height and waist-to-hip ratio and pancreatic cancer cannot be considered conclusive. For fasting insulin, we observed a 66% increased risk of pancreatic cancer per SD increase (44.4 pmol/L), although the strength of the association was marginal (*P* = .03), especially if the number of comparisons is considered. This association was driven by a strong risk increase in men (*P* = .003), whereas no association was seen in women. Conversely, we did not identify any risk increase for type 2 diabetes, nor for glucose levels. Finally, we found little evidence of a causal relationship between lipid parameters and pancreatic cancer risk. However, potential modest effects cannot be discarded, especially in the case of triglycerides.

Obesity and additional metabolic factors are associated with risk of several cancers (16), but traditional observational studies have had difficulties disentangling and establishing causality for the individual factors. Our results would support a direct role for obesity and the insulin pathway in pancreatic cancer. One hypothesis that would be in line with our results is that obesity leads to increasing insulin levels and risk of hyperinsulinemia, which in turns decreases insulin-like growth factor (IGF) binding proteins, thus allowing increasing circulating levels of insulin-like growth factor I (IGF1) (41–43). Both insulin and IGF1 are promoters of cell proliferation and inhibition of apoptosis in tumor cells (16,41,44,45). The interaction of the insulin pathway with sex hormones could explain the observed sex differences in terms of risk and deserves further investigation (43). Our results also lend further support to classical epidemiological studies showing an association between elevated circulating insulin and pancreatic cancer risk, especially in men (13), but little evidence for a role of type 2 diabetes. The latter observation would be in line with some studies suggesting that the observational association between type 2 diabetes risk may be due to reverse causation, that is, type 2 diabetes reflecting an early manifestation of pancreatic cancer, rather than being a causal factor (14,15). As our results suggest a potential important causal role of fasting insulin, the occurrence of hyperinsulinemia in early type 2 diabetes (46) would also be in line with insulin acting as a confounder for any observed association between type 2 diabetes and pancreatic cancer risk.

The main limitation in MR studies is the potential violation of assumptions of linearity and pleiotropy. Firstly, our MR analysis assumes a linear relation between each genetic instrument and the risk factor of interest, as well as a log-linear association between the risk factors and pancreatic cancer risk. It is not possible to test these assumptions with current data, but deviations from these assumptions would result in reduced statistical power in risk analyses, rather than generating spurious associations. However, the estimated effects may not be representative of the effects of the traits in the extremes of their distributions. Therefore, some caution is needed for the general interpretation of the results. In regards to pleiotropy, the use of complementary MR approaches and the visual inspection of funnel plots allowed us to evaluate the presence of pleiotropic effects on the instrumental SNPs. This is of particular concern for metabolic traits, where potential genetic confounding from BMI could bias initial estimates. Our additional analyses did not, however, indicate that pleiotropic effects were biasing the risk associations of BMI and fasting insulin.

In conclusion, using a two-sample MR approach, this study assessed a range of metabolic factors in relation to pancreatic cancer risk. Our results suggest that increases in BMI and fasting insulin are causally associated with an increased risk of pancreatic cancer. These findings provide important novel evidence on the etiology of pancreatic cancer.

## Funding

This work was supported by the Cancer Research UK (C18281/A19169) Programme Grant (the Integrative Cancer Epidemiology Programme), the National Institute for Health Research (NIHR) Bristol Nutritional Biomedical Research Unit based at University Hospitals Bristol NHS Foundation Trust and the University of Bristol (to RMM), the MRC Integrative Epidemiology Unit at the University of Bristol (MC_UU_12013/1, MC_UU_12013/2 to CLR and GDS), and the Cancer Research UK Population Research Postdoctoral Fellowship (C52724/A20138 to PCH).

## Notes

The funders had no role in the design of the study; the collection, analysis, or interpretation of the data; the writing of the manuscript; or the decision to submit the manuscript for publication.

## Supplementary Material

Supplementary Data

## References

[1] Torre LA, Bray F, Siegel RL, Ferlay J. Global Cancer Statistics, 2012. CA Cancer J Clin. 2015;65(2):87–108. 25651787 10.3322/caac.21262

[2] Canto MI, Harinck F, Hruban RH, International Cancer of the Pancreas Screening (CAPS) Consortium summit on the management of patients with increased risk for familial pancreatic cancer. Gut. 2013;62:339–347. 23135763 10.1136/gutjnl-2012-303108 PMC3585492

[3] Lauby-Secretan B, Scoccianti C, Loomis D, Body fatness and cancer—viewpoint of the IARC Working Group. N Engl J Med. 2016;375(8):794–798. 27557308 10.1056/NEJMsr1606602 PMC6754861

[4] World Cancer Research Fund/American Institute for Cancer Research. Food, Nutrition, Physical Activity, and the Prevention of Cancer: A Global Perspective. 2007 http://www.aicr.org/assets/docs/pdf/reports/Second_Expert_Report.pdf. Accessed March 24, 2017.

[5] Bosetti C, Lucenteforte E, Silverman DT, Cigarette smoking and pancreatic cancer: An analysis from the International Pancreatic Cancer Case-Control Consortium (Panc4). Ann Oncol. 2012;23:280–284. 22104574 10.1093/annonc/mdr541 PMC3387822

[6] Maisonneuve P, Lowenfels AB. Risk factors for pancreatic cancer: A summary review of meta-analytical studies. Int J Epidemiol. 2015;44(1):186–198. 25502106 10.1093/ije/dyu240

[7] Renehan AG, Tyson M, Egger M, Heller RF, Zwahlen M. Body-mass index and incidence of cancer: A systematic review and meta-analysis of prospective observational studies. Lancet. 2008;371:569–578. 18280327 10.1016/S0140-6736(08)60269-X

[8] Aune D, Greenwood DC, Chan DSM, Body mass index, abdominal fatness and pancreatic cancer risk: A systematic review and non-linear dose—response meta-analysis of prospective studies. Ann Oncol. 2012;23:843–852. 21890910 10.1093/annonc/mdr398

[9] Urayama KY, Holcatova I, Janout V, Body mass index and body size in early adulthood and risk case—control study. Int J Cancer. 2011;129:2875–2884. 21520034 10.1002/ijc.25959 PMC3146968

[10] Aune D, Rita A, Doris V, Man S, Norat T. Height and pancreatic cancer risk: A systematic review and meta-analysis of cohort studies. Cancer Causes Control. 2012;23:1213–1222. 22689322 10.1007/s10552-012-9983-0

[11] Chen H, Qin S, Wang M, Zhang T, Zhang S. Association between cholesterol intake and pancreatic cancer risk: Evidence from a meta-analysis. Sci Rep. 2015;5:8243–8248. 25649888 10.1038/srep08243 PMC4316166

[12] Liao W-C, Tu Y-K, Wu M-S, Lin J-T, Wang H-P, Chien K-L. Blood glucose concentration and risk of pancreatic cancer: Systematic review and dose-response meta-analysis. BMJ. 2015;349:g7371. 10.1136/bmj.g7371 PMC4282179 25556126

[13] Pisani P. Hyper-insulinaemia and cancer, meta-analyses of epidemiological studies. Arch Physiol Biochem. 2008;114:63–70. 18465360 10.1080/13813450801954451

[14] Elena JW, Steplowski E, Yu K, Diabetes and risk of pancreatic cancer: A pooled analysis from the pancreatic cancer cohort consortium. Cancer causes Control. 2013;24:13–25. 23112111 10.1007/s10552-012-0078-8 PMC3529822

[15] Huxley R, Ansary-Moghaddam A, Berrington de González A, Barzi F, Woodward M. Type-II diabetes and pancreatic cancer: A meta-analysis of 36 studies. Br J Cancer. 2005;92(11):2076–2083. 15886696 10.1038/sj.bjc.6602619 PMC2361795

[16] Esposito K, Chiodini P, Colao A, Lenzi A, Giugliano D. Metabolic syndrome and risk of cancer: A systematic review and meta-analysis. Diabetes Care. 2012;35:2402–2411. 23093685 10.2337/dc12-0336 PMC3476894

[17] Davey Smith G, Ebrahim S. ‘Mendelian randomization’: Can genetic epidemiology contribute to understanding environmental determinants of disease? Int J Epidemiol. 2003;(32):1–22. 12689998 10.1093/ije/dyg070

[18] Davey Smith G, Hemani G. Mendelian randomization: Genetic anchors for causal inference in epidemiological studies. Hum Mol Genet. 2014;23(1):1–10. 25064373 10.1093/hmg/ddu328 PMC4170722

[19] Burgess S, Butterworth AS, Thompson JR. Beyond Mendelian randomization: How to interpret evidence of shared genetic predictors. J Clin Epidemiol. 2016;69:208–216. 26291580 10.1016/j.jclinepi.2015.08.001 PMC4687951

[20] Petersen GM, Amundadottir L, Fuchs CS, A genome-wide association study identifies pancreatic cancer susceptibility loci on chromosomes 13q22.1, 1q32.1 and 5p15.33. Nat Genet. 2010;42(3):224–228. 20101243 10.1038/ng.522 PMC2853179

[21] Amundadottir L, Kraft P, Stolzenberg-solomon RZ, Genome-wide association study identifies variants in the ABO locus associated with susceptibility to pancreatic cancer. Nat Genet. 2009;41(9):986–990. 19648918 10.1038/ng.429 PMC2839871

[22] Childs EJ, Mocci E, Campa D, Common variation at 2p13.3, 3q29, 7p13 and 17q25.1 associated with susceptibility to pancreatic cancer. Nat Genet. 2015;47(8):911–916. 26098869 10.1038/ng.3341 PMC4520746

[23] Tryka KA, Hao L, Sturcke A, NCBI’s database of genotypes and phenotypes: DbGaP. Nucleic Acids Res. 2014;42(D1):975–979. 10.1093/nar/gkt1211 PMC3965052 24297256

[24] Burgess S, Butterworth A, Thompson SG. Mendelian randomization analysis with multiple genetic variants using summarized data. Genet Epidemiol. 2013;37(7):658–665. 24114802 10.1002/gepi.21758 PMC4377079

[25] Burgess S, Scott RA, Timpson NJ, Davey-smith G, Thompson SG. Using published data in Mendelian randomization: A blueprint for efficient identification of causal risk factors. Eur J Epidemiol. 2014;(100114):1–39. 25773750 10.1007/s10654-015-0011-z PMC4516908

[26] Wood AR, Esko T, Yang J, Defining the role of common variation in the genomic and biological architecture of adult human height. Nat Genet. 2014;46(11):1173–1186. 25282103 10.1038/ng.3097 PMC4250049

[27] Locke AE, Kahali B, Berndt SI, Genetic studies of body mass index yield new insights for obesity biology. Nature. 2015;518:197–206. 25673413 10.1038/nature14177 PMC4382211

[28] Shungin D, Winkler TW, Croteau-Chonka DC, New genetic loci link adipose and insulin biology to body fat distribution. Nature. 2015;518:187–196. 25673412 10.1038/nature14132 PMC4338562

[29] Willer CJ, Schmidt EM, Sengupta S, Discovery and refinement of loci associated with lipid levels. Nat Genet. 2013;45(11):1274–1283. 24097068 10.1038/ng.2797 PMC3838666

[30] Scott RA, Lagou V, Welch RP, Wheeler E, May E. Large-scale association analyses identify new loci influencing glycemic traits and provide insight into the underlying biological pathways. Nat Genet. 2012;44(9):991–1005. 22885924 10.1038/ng.2385 PMC3433394

[31] Morris AP, Voight BF, Teslovich TM, Large-scale association analysis provides insights into the genetic architecture and pathophysiology of type 2 diabetes. Nat Genet. 2012;44(9):981–989. 22885922 10.1038/ng.2383 PMC3442244

[32] Gaulton KJ, Ferreira T, Lee Y, Genetic fine mapping and genomic annotation defines causal mechanisms at type 2 diabetes susceptibility loci. Nat Genet. 2015;47(12):1415–1425. 26551672 10.1038/ng.3437 PMC4666734

[33] Michigan Imputation Server. https://imputationserver.sph.umich.edu/index.html. Accessed March, 2016.

[34] Delaneau O, Marchini J, Zagury J-F. A linear complexity phasing method for thousands of genomes. Nat Methods. 2012;9:179–181. 10.1038/nmeth.1785 22138821

[35] Howie B, Fuchsberger C, Stephens M, Marchini J, Abecasis GR. Fast and accurate genotype imputation in genome-wide association studies through pre-phasing. Nat Genet. 2012;44(8):955–959. 22820512 10.1038/ng.2354 PMC3696580

[36] Haplotype Reference Consortium. http://www.haplotype-reference-consortium.org/home. Accessed March 24, 2017.

[37] Willer CJ, Li Y, Abecasis GR. METAL: Fast and efficient meta-analysis of genomewide association scans. Bioinformatics. 2010;26(17):2190–2191. 20616382 10.1093/bioinformatics/btq340 PMC2922887

[38] Burgess S. Sample size and power calculations in Mendelian randomization with a single instrumental variable and a binary outcome. Int J Epidemiol. 2014;43(3):922–929. 24608958 10.1093/ije/dyu005 PMC4052137

[39] Bowden J, Smith GD, Haycock PC, Burgess S. Consistent estimation in Mendelian randomization with some invalid instruments using a weighted median estimator. Genet Epidemiol. 2016;40(4):304–314. 27061298 10.1002/gepi.21965 PMC4849733

[40] Bowden J, Davey Smith G, Burgess S. Mendelian randomization with invalid instruments: Effect estimation and bias detection through Egger regression. Int J Epidemiol. 2015;44(2):512–525. 26050253 10.1093/ije/dyv080 PMC4469799

[41] Braun S, Bitton-worms K, Leroith D. The link between the metabolic syndrome and cancer. Int J Biol Sci. 2011;7(7):1003–1015. 21912508 10.7150/ijbs.7.1003 PMC3164150

[42] Kahn BB, Flier JS. Obesity and insulin resistance. J Clin Invest. 2000;106(4):473–481. 10953022 10.1172/JCI10842 PMC380258

[43] Renehan AG, Zwahlen M, Egger M. Adiposity and cancer risk: New mechanistic insights from epidemiology. Nat Rev Cancer. 2015;15(8):484–498. 26205341 10.1038/nrc3967

[44] Hezel AF, Kimmelman AC, Stanger BZ, Bardeesy N, Depinho RA. Genetics and biology of pancreatic ductal adenocarcinoma. Genes Dev. 2006;20:1218–1249. 16702400 10.1101/gad.1415606

[45] Pothiwala P, Jain SK, Ph D, Yaturu S. Metabolic syndrome and cancer. Metab Syndr Relat Disord. 2009;7(4):279–288. 19284314 10.1089/met.2008.0065 PMC3191378

[46] Ammon HPT. Hyper- and hypoinsulinemia in type-2 diabetes: What may be wrong in the secretory mechanism of the B-cell. Exp Clin Endocrinol Diabetes. 1997;105(suppl 2):43–47. 10.1055/s-0029-1211796 9288544

