# Supplementary material for: The Role of Obesity, Type 2 Diabetes, and Metabolic Factors in Pancreatic Cancer: A Mendelian Randomization Study
Source: J Natl Cancer Inst. 2017 Apr 28;109(9):djx012. doi: 10.1093/jnci/djx012 (PMC5721813; doi:10.1093/jnci/djx012)
Supplement: Supplementary Data [file 16-1194r2_brennan_supp_mat_011317_djx012.docx]

**SUPPLEMENTARY MATERIALS**

**The role of obesity, type 2 diabetes and metabolic factors in pancreatic cancer: A Mendelian randomization study.**

Robert Carreras-Torres^1¶^, Mattias Johansson^1¶^, Valerie Gaborieau^1^, Philip C Haycock^2^, Kaitlin H Wade^2^, Caroline L Relton^2^, Richard M Martin^2,3^, George Davey Smith^2^, Paul Brennan^1*^

^1^ Section of Genetics, International Agency for Research on Cancer (IARC), Lyon, France.

^2^ MRC Integrative Epidemiology Unit, School of Social and Community Medicine, University of Bristol, Bristol, UK.

^3^ National Institute for Health Research Biomedical Research Unit in Nutrition, Diet and Lifestyle at University Hospitals Bristol NHS Foundation Trust and the University of Bristol, BS2 8AE, Bristol, UK.

^¶^ Contributed equally

*Corresponding Author:

Paul Brennan, Ph.D.

Section of Genetics

International Agency for Research on Cancer (IARC)

Lyon, France

+33 (0)4 72 73 85 33

[gep@iarc.fr](mailto:gep@iarc.fr)

**Supplementary Table 1. Detailed sample characteristics form PanScan and PanC4 consortia**

| **PanScan samples** |  |  |  |  |  |  |  |  |  |  |  |  |  |
| --- | --- | --- | --- | --- | --- | --- | --- | --- | --- | --- | --- | --- | --- |
| **Cohort** | **Location** |  |  | **Matching of controls to cases** | **Enrolment** | **Mean time to case diagnosis (years)** | **n Cases** | **n Controls** | **Case diagnosis age, mean (SD)** |  | **Male (%)** | **Caucasian (%)** | **Genotyping Array** |
| The Alpha-Tocopherol, Beta-Carotene Prevention Study (ATBC) | Finland |  |  | Race, sex, age, date of blood draw, alive at the time the case was diagnosed | 1985-1988 | 6.6 | 194 | 206 | 70.5  (5.6) |  | 100 | 100 | HumanHap500 |
| Give Us a Clue to Cancer and Heart Disease Study (CLUEII) | Washington County MD, USA |  |  | Race, sex, age, date of blood draw, alive at the time the case was diagnosed | 1989 | 8.5 | 68 | 71 | 69.7 (11.3) |  | 46 | 100 | HumanHap500 |
| Cancer Prevention Study (CPS II) | USA |  |  | Race, sex, birth year, DNA source (blood or buccal), alive at the time the case was diagnosed | 1992-1993 | 1.9 | 120 | 118 | 75.0  (5.8) |  | 56.7 | 97.9 | HumanHap500 |
| European Prospective Investigation Into Cancer and Nutrition Study (EPIC) | Europe |  |  | Race, sex, age, date of blood draw, alive at the time the case was diagnosed | 1988-2002 | 5 | 420 | 436 | 62.1  (7.8) |  | 46.6 | 100 | HumanHap500 |
| Health Professionals Follow-up Study (HFPS) | USA |  |  | Sex, age, date of blood draw, alive at the time the case was diagnosed, smoking status (never/former/current) | 1986 | 5.2 | 53 | 51 | 71.1  (7.8) |  | 100 | 100 | HumanHap500 |
| Nurse's Health Study (NHS) | USA |  |  | Race, sex, age, date of blood draw, alive at the time the case was diagnosed, smoking status (never/former/current) | 1976 | 8 | 82 | 84 | 69.6  (6.4) |  | 0 | 91.6 | HumanHap500 |
| The New York University Women’s Health Study (NYU-WHS) | USA |  |  | Race, sex, age, date of blood draw, alive at the time the case was diagnosed, menopausal status at enrolment | 1985-1990 | 8.5 | 13 | 13 | 67.9 (10.0) |  | 0 | 76.9 | HumanHap500 |
| Physicians Health Study (PHS) | USA |  |  | Race, sex, age, date of blood draw, alive at the time the case was diagnosed, smoking status (never/former/current) | 1982-1983 | 13.4 | 49 | 54 | 70.4 (10.0) |  | 100 | 79.6 | HumanHap500 |
| Prostate, Lung, Colorectal, Ovarian Cancer Screening Trial (PLCO) | USA |  |  | Race, sex, age, birth year, date of blood draw, alive at the time the case was diagnosed, DNA source (blood or buccal cell), study arm, and center | 1994-2001 | 6.1 | 201 | 222 | 71.7  (5.9) |  | 61 | 91.7 | HumanHap500 |
| Shanghai Men’s and Women’s Health Study (SMWHS) | China |  |  | Sex, age, birth year, menopausal status at baseline, date of blood draw, alive at the time the case was diagnosed | 1996 (F) | 3.3 | 61 | 65 | 66.3  (7.4) |  | 20.6 | 0 | HumanHap500 |
| Women’s Health Initiative (WHI) | USA |  |  | Race, sex, age, center, enrolment date, study arm, hysterectomy status, menopausal status, alive at the time the case was diagnosed | 1992-1998 | 4.2 | 242 | 242 | 71.8  (7.5) |  | 0 | 85.5 | HumanHap500 |
| Womens' Heath Study (WHS) | USA |  |  | Race, age, birth year, smoking status (never/former/current) date of blood draw, alive at the time the case was diagnosed | 1992-1993 | 3.9 | 25 | 32 | 61.9  (8.4) |  | 0 | 94.7 | HumanHap500 |
|  |  |  |  |  |  |  |  |  |  |  |  |  |  |
| **Cohort total** |  |  |  |  | **1976-2004** | **5.5** | **1528** | **1594** | **68.5  (8.5)** |  | **47.7** | **91** |  |
|  |  |  |  |  |  |  |  |  |  |  |  |  |  |
|  |  |  |  |  |  |  |  |  |  |  |  |  |  |
|  |  |  |  |  |  |  |  |  |  |  |  |  |  |
| **PanC4 samples** |  |  |  |  |  |  |  |  |  |  |  |  |  |
| **Case-Control** | **Location** | **Source of cases** | **Source of controls** | **Matching method and variables** | **Date of case diagnosis** | **Date of control recruitment** | **n Cases** | **n Controls** | **Case diagnosis age, mean (SD)** | **Age of controls, mean (SD)** | **Male (%)** | **Caucasian (%)** | **Genotyping Array** |
| IARC | France | Academic hospitals | General practitioners | Age, sex, region | 2006-2010 |  | 448 | 456 | 63.84 (11.16) | 61.88 (11.88) | 57 | 100 | Human OmniExpress Exome-8v1 |
| Johns Hopkins Hospital | Baltimore, USA | JHU Clinic | Spouse (in-Law) of Pancreatic Cancer Patient, no history pancreatic cancer | None | 1996-2007 | 1996-2007 | 201 | 190 | 64.5 (10.4) |  | 44.3 | 95.9 | Human  610-Quad |
|  |  |  |  |  | 2007-2011 |  | 315 | 81 | 64.29 (11.53) | 63.45 (14.73) | 51 | 93 | Human OmniExpress Exome-8v1 |
| Mayo Clinic Molecular Epidemiology Case-Control Study | Upper Mid-West: Minn., Iowa, Wisconsin, USA | Clinic | General Medical Evaluation (primary care) patients, no personal history of cancer personal history of cancer skin) | Frequency matched by age, race, sex, and residence | 1991-2008 | 2004-2007 | 651 | 622 | 66.3 (10.3) |  | 54.7 | 98.6 | Human  610-Quad |
|  |  |  |  |  | 2000-2010 |  | 1104 | 1027 | 65.92 (11.09) | 63.34 (10.58) | 56 | 93 | Human OmniExpress Exome-8v1 |
| MD Anderson | Texas, USA | Hospital | Friends and spouses of nonpancreatic cancer patients at MDA, no personal history of cancer (except nonmelanoma of skin) | Frequency matched by ange, race, and sex | 1997-2007 | 2004-2007 | 616 | 509 | 62.6 (9.69) | 59.2 (10.6) | 59 | 100 | Human OmniExpress Exome-8v1 |
| Memorial Sloan Kettering | NYC, USA | Clinic | Spouses of patients; visitors accompanying patients, no personal history of cancer (except non-melanoma of skin) | None | 2000-2008 |  | 317 | 139 | 64.03 (10.39) | 61.68 (10.98) | 64 | 87 | Human OmniExpress Exome-8v1 |
| PACIFIC Study | Seattle, WA and Northern California, USA | Group Health (Seattle Pufet Sound) and Kaiser Permanente Northern CA) | Group Health (Seattle Pufet Sound) and Kaiser Permanente Northern CA) | Frequency matched by race, age, enrolment duration in HMO, and sex | 2005-2008 | 2005-2008 | 276 | 270 | 68.7 (11.1) |  | 50 | 100 | Human  610-Quad |
| Queensland |  | Population-based hospitals & cancer registry | Electoral rolls | Age, sex | 2007-2011 |  | 559 | 604 | 66.64 (11.26) | 67.51 (10.91) | 60 | 96 | Human OmniExpress Exome-8v1 |
| Toronto | Ontario (cases) Greater Toronto (controls), Cananda | Population-based cancer registry | Ontario population-based case control study of colorectal cancer, ARCTIC | Age, gender, and ethnicity. No personal history of personal history of colorectal cancer | 2000-2008 | 1999-2003 | 294 | 275 | 63.7 (10.0) |  | 54.1 | 94.6 | Human  610-Quad |
|  |  |  |  |  | 2003-2012 |  | 402 | 401 | 64.92 (11.03) | 62.95 (11.73) | 50 | 85 | Human OmniExpress Exome-8v1 |
| UCSF | San Francisco Bay Area, USA | Population-based cancer registry | Random digit-dial within six SF Bay Area counties, no history pancreatic cancer | Frequency matched by sex and age | 1993-1999 | 1995-1999 | 265 | 267 | 64.9 (10.7) |  | 54 | 84.8 | Human  610-Quad |
|  |  |  |  |  | 2006-2010 |  | 253 | 248 | 62.52 (10.35) | 60.4 (10.96) | 54 | 82 | Human OmniExpress Exome-8v1 |
| Yale | Connecticut, USA | Population-based cancer registry | Block list-directed Random digit-dial, no personal history of cancer (except non-melanoma of skin) | Frequency matched by sex and age | 2004-2008 | 2005-2008 | 221 | 229 | 66.9  (9.9) |  | 54.2 | 94.2 | Human  610-Quad |
|  |  |  |  |  | 2005-2009 |  | 156 | 366 | 67.02 (10.43) | 65.15 (10.6) | 59 | 93 | Human OmniExpress Exome-8v1 |
|  |  |  |  |  |  |  |  |  |  |  |  |  |  |
| **Case-control total** |  |  |  |  |  |  | **6078** | **5684** |  |  |  |  |  |

**Supplementary Table 2- Association parameters of instrumental SNPs of potential risk factors for pancreatic cancer***

|  |  |  |  |  |  | **SNP to phenotype effect (β_GP_)** | | **SNP to disease effect (β_GD_)** | | | | | | |  |  |
| --- | --- | --- | --- | --- | --- | --- | --- | --- | --- | --- | --- | --- | --- | --- | --- | --- |
|  |  |  |  |  |  |  |  | **Meta-analyses** | | **PanScan I** | | **PanScan II** | | **PanC4** | |  |
| **SNP** | **Chr** | **Position** | **Mean imputation quality** | **Effect allele** | **Other allele** | **Estimate** | **SE** | **Estimate** | **SE** | **Estimate** | **SE** | **Estimate** | **SE** | **Estimate** | **SE** | **Trait** |
| rs425277 | 1 | 2069172 | 1.00 | T | C | 0.03 | 0.00 | -0.04 | 0.03 | -0.10 | 0.05 | -0.08 | 0.06 | 0.01 | 0.04 | Height |
| rs9434723 | 1 | 9292282 | 0.97 | A | G | 0.03 | 0.00 | 0.01 | 0.03 | -0.04 | 0.07 | 0.10 | 0.07 | -0.01 | 0.05 | Height |
| rs10779751 | 1 | 11284336 | 0.99 | A | G | 0.02 | 0.00 | 0.04 | 0.03 | -0.04 | 0.05 | 0.15 | 0.06 | 0.03 | 0.04 | Height |
| rs12137162 | 1 | 19763396 | 1.00 | A | C | 0.02 | 0.00 | 0.00 | 0.03 | 0.01 | 0.05 | -0.04 | 0.06 | 0.01 | 0.04 | Height |
| rs212524 | 1 | 21583311 | 1.00 | C | T | 0.02 | 0.00 | -0.03 | 0.02 | -0.05 | 0.05 | 0.00 | 0.05 | -0.03 | 0.03 | Height |
| rs2806561 | 1 | 23504795 | 1.00 | A | G | 0.03 | 0.00 | 0.00 | 0.02 | -0.01 | 0.05 | 0.07 | 0.05 | -0.02 | 0.03 | Height |
| rs4601530 | 1 | 25044111 | 1.00 | C | T | 0.03 | 0.00 | 0.00 | 0.03 | 0.03 | 0.05 | -0.04 | 0.06 | 0.00 | 0.04 | Height |
| rs926438 | 1 | 25753638 | 0.99 | T | C | 0.02 | 0.00 | 0.00 | 0.02 | -0.01 | 0.05 | 0.06 | 0.05 | -0.02 | 0.03 | Height |
| rs17163588 | 1 | 26450009 | 1.00 | T | C | 0.03 | 0.00 | -0.02 | 0.03 | -0.04 | 0.06 | 0.01 | 0.07 | -0.03 | 0.04 | Height |
| rs2219320 | 1 | 26803430 | 0.99 | T | C | 0.02 | 0.00 | -0.05 | 0.03 | -0.08 | 0.06 | -0.01 | 0.06 | -0.05 | 0.04 | Height |
| rs16834765 | 1 | 32371442 | 1.00 | T | C | 0.05 | 0.01 | 0.05 | 0.05 | 0.00 | 0.10 | 0.13 | 0.10 | 0.02 | 0.07 | Height |
| rs7544462 | 1 | 37962756 | 1.00 | A | C | 0.03 | 0.01 | 0.03 | 0.05 | 0.05 | 0.09 | 0.16 | 0.09 | -0.04 | 0.06 | Height |
| rs209918 | 1 | 40777842 | 0.96 | A | G | 0.02 | 0.00 | -0.03 | 0.03 | -0.11 | 0.06 | 0.00 | 0.06 | 0.00 | 0.04 | Height |
| rs6600365 | 1 | 41556253 | 1.00 | C | T | 0.03 | 0.00 | 0.01 | 0.02 | 0.00 | 0.05 | 0.02 | 0.05 | 0.01 | 0.03 | Height |
| rs12855 | 1 | 51440093 | 1.00 | T | C | 0.04 | 0.01 | 0.03 | 0.04 | 0.02 | 0.08 | 0.00 | 0.09 | 0.06 | 0.06 | Height |
| rs17387330 | 1 | 54119578 | 0.99 | A | G | 0.02 | 0.00 | -0.01 | 0.03 | 0.00 | 0.05 | -0.05 | 0.05 | -0.01 | 0.03 | Height |
| rs6691924 | 1 | 54954245 | 0.99 | T | C | 0.03 | 0.01 | 0.03 | 0.04 | -0.12 | 0.08 | 0.08 | 0.08 | 0.09 | 0.05 | Height |
| rs2815379 | 1 | 67510474 | 1.00 | G | A | 0.02 | 0.00 | -0.02 | 0.03 | -0.06 | 0.05 | -0.07 | 0.06 | 0.02 | 0.04 | Height |
| rs17391694 | 1 | 78623626 | 1.00 | T | C | 0.04 | 0.01 | 0.04 | 0.04 | -0.03 | 0.07 | 0.02 | 0.08 | 0.09 | 0.05 | Height |
| rs567401 | 1 | 85988158 | 0.86 | T | C | 0.03 | 0.00 | 0.02 | 0.04 | -0.01 | 0.07 | 0.04 | 0.07 | 0.02 | 0.05 | Height |
| rs2046158 | 1 | 86330770 | 1.00 | C | T | 0.02 | 0.00 | 0.03 | 0.03 | 0.13 | 0.07 | 0.03 | 0.07 | -0.02 | 0.04 | Height |
| rs2811594 | 1 | 93343282 | 1.00 | G | A | 0.02 | 0.00 | 0.05 | 0.02 | -0.05 | 0.05 | 0.10 | 0.05 | 0.07 | 0.03 | Height |
| rs17113369 | 1 | 95787223 | 1.00 | T | C | 0.07 | 0.01 | 0.07 | 0.06 | 0.04 | 0.12 | 0.10 | 0.13 | 0.07 | 0.08 | Height |
| rs7517682 | 1 | 103519589 | 0.99 | G | A | 0.02 | 0.00 | 0.01 | 0.02 | 0.02 | 0.05 | -0.03 | 0.05 | 0.02 | 0.03 | Height |
| rs12120956 | 1 | 113202571 | 1.00 | G | A | 0.02 | 0.00 | -0.04 | 0.03 | -0.04 | 0.06 | -0.05 | 0.06 | -0.04 | 0.04 | Height |
| rs1321666 | 1 | 118492052 | 1.00 | C | T | 0.02 | 0.00 | 0.01 | 0.02 | 0.04 | 0.05 | 0.02 | 0.05 | 0.00 | 0.03 | Height |
| rs9428104 | 1 | 118855587 | 0.98 | G | A | 0.04 | 0.00 | 0.00 | 0.03 | -0.05 | 0.06 | 0.02 | 0.06 | 0.01 | 0.04 | Height |
| rs1409156 | 1 | 119491784 | 0.99 | G | A | 0.02 | 0.00 | -0.05 | 0.03 | 0.01 | 0.05 | -0.08 | 0.05 | -0.07 | 0.03 | Height |
| rs6658763 | 1 | 146692373 | 1.00 | C | T | 0.03 | 0.01 | 0.01 | 0.04 | -0.14 | 0.09 | 0.04 | 0.09 | 0.07 | 0.06 | Height |
| rs3767627 | 1 | 149938898 | 1.00 | T | C | 0.03 | 0.00 | 0.01 | 0.03 | 0.00 | 0.07 | 0.10 | 0.07 | -0.02 | 0.05 | Height |
| rs956796 | 1 | 150186091 | 1.00 | G | A | 0.03 | 0.01 | -0.02 | 0.04 | -0.04 | 0.08 | 0.00 | 0.08 | -0.02 | 0.05 | Height |
| rs2298265 | 1 | 151259043 | 1.00 | C | T | 0.03 | 0.00 | 0.00 | 0.04 | -0.04 | 0.07 | -0.06 | 0.08 | 0.06 | 0.05 | Height |
| rs6688100 | 1 | 160399586 | 1.00 | T | C | 0.02 | 0.00 | -0.05 | 0.02 | -0.02 | 0.05 | -0.05 | 0.05 | -0.07 | 0.03 | Height |
| rs4656220 | 1 | 170649277 | 1.00 | T | C | 0.02 | 0.00 | 0.03 | 0.02 | 0.10 | 0.05 | -0.08 | 0.05 | 0.05 | 0.03 | Height |
| rs6694089 | 1 | 172083881 | 1.00 | A | G | 0.03 | 0.00 | -0.03 | 0.03 | 0.09 | 0.05 | -0.02 | 0.06 | -0.09 | 0.04 | Height |
| rs2421992 | 1 | 172241251 | 1.00 | T | C | 0.03 | 0.00 | -0.03 | 0.03 | -0.05 | 0.05 | 0.03 | 0.05 | -0.05 | 0.04 | Height |
| rs17369123 | 1 | 172355841 | 1.00 | T | C | 0.03 | 0.00 | 0.01 | 0.03 | 0.00 | 0.06 | -0.01 | 0.07 | 0.03 | 0.04 | Height |
| rs1325596 | 1 | 176794066 | 1.00 | A | G | 0.03 | 0.00 | 0.06 | 0.02 | 0.06 | 0.05 | 0.11 | 0.05 | 0.03 | 0.03 | Height |
| rs4652773 | 1 | 183054827 | 1.00 | A | G | 0.02 | 0.00 | 0.03 | 0.02 | 0.00 | 0.05 | 0.09 | 0.05 | 0.02 | 0.03 | Height |
| rs3814333 | 1 | 184007119 | 1.00 | T | C | 0.05 | 0.00 | -0.01 | 0.03 | 0.00 | 0.05 | -0.01 | 0.05 | -0.01 | 0.04 | Height |
| rs10863936 | 1 | 212237798 | 1.00 | G | A | 0.02 | 0.00 | -0.02 | 0.02 | -0.03 | 0.05 | -0.08 | 0.05 | 0.01 | 0.03 | Height |
| rs6540834 | 1 | 214627419 | 1.00 | C | T | 0.03 | 0.00 | -0.02 | 0.02 | -0.08 | 0.05 | 0.05 | 0.05 | -0.02 | 0.03 | Height |
| rs1244981 | 1 | 215046892 | 1.00 | A | G | 0.03 | 0.00 | -0.04 | 0.03 | -0.04 | 0.07 | 0.00 | 0.07 | -0.05 | 0.04 | Height |
| rs10495098 | 1 | 218516310 | 0.98 | T | G | 0.02 | 0.00 | -0.03 | 0.02 | 0.00 | 0.05 | -0.05 | 0.05 | -0.03 | 0.03 | Height |
| rs991967 | 1 | 218615451 | 1.00 | C | A | 0.04 | 0.00 | 0.03 | 0.03 | -0.04 | 0.05 | 0.07 | 0.05 | 0.04 | 0.04 | Height |
| rs12411277 | 1 | 218975475 | 1.00 | G | A | 0.02 | 0.00 | 0.01 | 0.02 | 0.07 | 0.05 | 0.00 | 0.05 | -0.01 | 0.03 | Height |
| rs4428898 | 1 | 219739966 | 1.00 | G | A | 0.02 | 0.00 | -0.01 | 0.02 | 0.03 | 0.05 | -0.04 | 0.05 | -0.03 | 0.03 | Height |
| rs1544196 | 1 | 224632782 | 1.00 | G | A | 0.02 | 0.00 | 0.01 | 0.03 | 0.07 | 0.06 | 0.04 | 0.06 | -0.02 | 0.04 | Height |
| rs6696239 | 1 | 227750068 | 1.00 | G | A | 0.04 | 0.00 | -0.01 | 0.03 | -0.03 | 0.06 | 0.00 | 0.06 | -0.01 | 0.04 | Height |
| rs11799609 | 1 | 243618317 | 1.00 | T | G | 0.03 | 0.00 | 0.01 | 0.03 | 0.07 | 0.07 | 0.00 | 0.07 | -0.02 | 0.05 | Height |
| rs17038954 | 2 | 1645673 | 0.99 | T | C | 0.04 | 0.01 | 0.02 | 0.05 | -0.04 | 0.10 | 0.07 | 0.10 | 0.02 | 0.07 | Height |
| rs10048625 | 2 | 1775648 | 0.97 | T | C | 0.02 | 0.00 | -0.01 | 0.03 | -0.08 | 0.06 | -0.03 | 0.07 | 0.02 | 0.04 | Height |
| rs3885668 | 2 | 10178479 | 1.00 | C | T | 0.02 | 0.00 | 0.02 | 0.02 | -0.02 | 0.05 | 0.06 | 0.05 | 0.02 | 0.03 | Height |
| rs2345835 | 2 | 18574952 | 1.00 | C | T | 0.02 | 0.00 | 0.01 | 0.02 | 0.02 | 0.05 | 0.01 | 0.05 | 0.01 | 0.03 | Height |
| rs7561273 | 2 | 24247514 | 1.00 | G | A | 0.02 | 0.00 | 0.02 | 0.02 | 0.03 | 0.05 | 0.03 | 0.05 | 0.01 | 0.03 | Height |
| rs2278483 | 2 | 25040082 | 1.00 | T | C | 0.03 | 0.00 | -0.01 | 0.03 | -0.01 | 0.06 | -0.01 | 0.06 | -0.01 | 0.04 | Height |
| rs2289195 | 2 | 25463483 | 0.99 | A | G | 0.04 | 0.00 | 0.02 | 0.02 | 0.01 | 0.05 | 0.03 | 0.05 | 0.02 | 0.03 | Height |
| rs10460566 | 2 | 25483121 | 1.00 | G | A | 0.02 | 0.00 | -0.02 | 0.03 | -0.05 | 0.06 | -0.06 | 0.06 | 0.01 | 0.04 | Height |
| rs780094 | 2 | 27741237 | 1.00 | C | T | 0.02 | 0.00 | 0.01 | 0.02 | 0.00 | 0.05 | 0.08 | 0.05 | -0.02 | 0.03 | Height |
| rs6714546 | 2 | 33361425 | 0.99 | G | A | 0.04 | 0.00 | 0.05 | 0.03 | 0.15 | 0.05 | -0.11 | 0.06 | 0.07 | 0.04 | Height |
| rs6751657 | 2 | 33405151 | 1.00 | C | T | 0.02 | 0.00 | -0.02 | 0.02 | 0.02 | 0.05 | -0.02 | 0.05 | -0.05 | 0.03 | Height |
| rs711245 | 2 | 36768875 | 1.00 | G | A | 0.02 | 0.00 | 0.04 | 0.03 | 0.02 | 0.05 | 0.09 | 0.05 | 0.02 | 0.03 | Height |
| rs6544089 | 2 | 37758745 | 0.99 | T | C | 0.02 | 0.00 | -0.01 | 0.02 | -0.07 | 0.05 | -0.03 | 0.05 | 0.03 | 0.03 | Height |
| rs13416119 | 2 | 42462930 | 0.99 | A | G | 0.03 | 0.01 | -0.03 | 0.04 | 0.07 | 0.08 | -0.10 | 0.09 | -0.04 | 0.06 | Height |
| rs9309101 | 2 | 43629612 | 1.00 | G | A | 0.02 | 0.00 | 0.00 | 0.02 | 0.01 | 0.05 | -0.06 | 0.05 | 0.03 | 0.03 | Height |
| rs897080 | 2 | 44774202 | 0.99 | C | T | 0.03 | 0.00 | 0.01 | 0.03 | 0.04 | 0.06 | -0.06 | 0.06 | 0.02 | 0.04 | Height |
| rs17032525 | 2 | 44907331 | 0.99 | A | G | 0.03 | 0.00 | 0.03 | 0.03 | 0.02 | 0.07 | -0.06 | 0.07 | 0.07 | 0.05 | Height |
| rs12474201 | 2 | 46921285 | 0.99 | A | G | 0.03 | 0.00 | -0.03 | 0.02 | 0.04 | 0.05 | 0.03 | 0.05 | -0.09 | 0.03 | Height |
| rs354196 | 2 | 54966407 | 0.99 | G | A | 0.02 | 0.00 | -0.03 | 0.02 | 0.01 | 0.05 | -0.14 | 0.05 | 0.00 | 0.03 | Height |
| rs1367226 | 2 | 56089540 | 1.00 | G | A | 0.04 | 0.00 | -0.02 | 0.02 | 0.02 | 0.05 | -0.03 | 0.05 | -0.04 | 0.03 | Height |
| rs3791679 | 2 | 56096892 | 1.00 | A | G | 0.08 | 0.00 | 0.03 | 0.03 | 0.06 | 0.06 | 0.00 | 0.06 | 0.03 | 0.04 | Height |
| rs3791673 | 2 | 56113538 | 0.99 | G | A | 0.05 | 0.01 | -0.03 | 0.06 | -0.08 | 0.11 | -0.09 | 0.12 | 0.02 | 0.08 | Height |
| rs2120335 | 2 | 68495002 | 0.96 | G | A | 0.02 | 0.00 | -0.01 | 0.02 | 0.02 | 0.05 | -0.02 | 0.05 | -0.02 | 0.03 | Height |
| rs7568069 | 2 | 71584485 | 1.00 | G | A | 0.02 | 0.00 | 0.01 | 0.02 | 0.07 | 0.05 | -0.03 | 0.05 | 0.01 | 0.03 | Height |
| rs11684404 | 2 | 88924622 | 0.99 | C | T | 0.03 | 0.00 | 0.00 | 0.03 | 0.01 | 0.05 | -0.07 | 0.05 | 0.03 | 0.04 | Height |
| rs11683207 | 2 | 98333290 | 0.99 | T | C | 0.02 | 0.00 | 0.01 | 0.03 | 0.02 | 0.06 | 0.02 | 0.06 | 0.00 | 0.04 | Height |
| rs13388725 | 2 | 109047190 | 0.99 | G | A | 0.02 | 0.00 | 0.01 | 0.02 | -0.04 | 0.05 | -0.01 | 0.05 | 0.04 | 0.03 | Height |
| rs2166898 | 2 | 121612659 | 1.00 | G | A | 0.03 | 0.00 | -0.01 | 0.03 | -0.03 | 0.06 | 0.02 | 0.07 | -0.02 | 0.04 | Height |
| rs7567288 | 2 | 134434824 | 0.98 | C | T | 0.03 | 0.00 | -0.03 | 0.03 | -0.05 | 0.06 | 0.10 | 0.07 | -0.07 | 0.04 | Height |
| rs4953951 | 2 | 136187345 | 0.99 | C | T | 0.03 | 0.01 | -0.01 | 0.04 | 0.06 | 0.08 | -0.02 | 0.08 | -0.04 | 0.05 | Height |
| rs749234 | 2 | 145231349 | 0.99 | A | G | 0.02 | 0.00 | -0.04 | 0.03 | -0.12 | 0.05 | 0.02 | 0.05 | -0.02 | 0.04 | Height |
| rs540652 | 2 | 169707428 | 0.99 | T | C | 0.02 | 0.00 | 0.02 | 0.02 | 0.06 | 0.05 | -0.01 | 0.05 | 0.01 | 0.03 | Height |
| rs12987566 | 2 | 172152646 | 0.99 | T | C | 0.02 | 0.00 | 0.01 | 0.03 | -0.03 | 0.05 | 0.00 | 0.06 | 0.03 | 0.04 | Height |
| rs6746356 | 2 | 174815898 | 1.00 | A | C | 0.02 | 0.00 | -0.07 | 0.03 | -0.10 | 0.06 | -0.02 | 0.06 | -0.08 | 0.04 | Height |
| rs833152 | 2 | 183219101 | 0.99 | C | A | 0.02 | 0.00 | 0.02 | 0.02 | 0.00 | 0.05 | 0.00 | 0.05 | 0.03 | 0.03 | Height |
| rs12693589 | 2 | 191832662 | 1.00 | C | T | 0.02 | 0.00 | 0.05 | 0.03 | 0.04 | 0.06 | 0.00 | 0.06 | 0.08 | 0.04 | Height |
| rs6435143 | 2 | 203194256 | 0.99 | A | C | 0.02 | 0.00 | -0.01 | 0.02 | 0.05 | 0.05 | -0.02 | 0.05 | -0.04 | 0.03 | Height |
| rs12329133 | 2 | 217935116 | 0.99 | C | T | 0.02 | 0.00 | 0.02 | 0.02 | 0.04 | 0.05 | -0.04 | 0.05 | 0.04 | 0.03 | Height |
| rs17181956 | 2 | 218146080 | 0.93 | C | T | 0.03 | 0.00 | 0.00 | 0.04 | 0.13 | 0.08 | -0.06 | 0.08 | -0.03 | 0.05 | Height |
| rs1864439 | 2 | 218616633 | 0.95 | T | C | 0.04 | 0.00 | 0.01 | 0.04 | -0.01 | 0.08 | 0.14 | 0.09 | -0.03 | 0.06 | Height |
| rs992157 | 2 | 219154781 | 1.00 | A | G | 0.02 | 0.00 | -0.02 | 0.02 | 0.02 | 0.05 | -0.03 | 0.05 | -0.03 | 0.03 | Height |
| rs4674354 | 2 | 219903723 | 1.00 | T | C | 0.02 | 0.00 | 0.00 | 0.03 | 0.05 | 0.06 | 0.01 | 0.06 | -0.04 | 0.04 | Height |
| rs12470505 | 2 | 219908369 | 1.00 | T | G | 0.05 | 0.00 | -0.02 | 0.04 | 0.00 | 0.08 | -0.03 | 0.09 | -0.02 | 0.06 | Height |
| rs16859517 | 2 | 219949184 | 1.00 | T | C | 0.07 | 0.01 | 0.00 | 0.06 | -0.17 | 0.13 | -0.12 | 0.14 | 0.15 | 0.09 | Height |
| rs12621643 | 2 | 223917983 | 0.97 | G | T | 0.02 | 0.00 | -0.01 | 0.03 | 0.03 | 0.05 | -0.05 | 0.05 | -0.02 | 0.04 | Height |
| rs6761041 | 2 | 225030129 | 0.97 | T | C | 0.02 | 0.00 | 0.01 | 0.02 | 0.00 | 0.05 | 0.01 | 0.05 | 0.01 | 0.03 | Height |
| rs6733349 | 2 | 232268312 | 1.00 | T | C | 0.02 | 0.00 | 0.01 | 0.03 | 0.02 | 0.05 | 0.00 | 0.05 | 0.01 | 0.04 | Height |
| rs6754426 | 2 | 232322779 | 0.96 | G | A | 0.02 | 0.00 | 0.02 | 0.02 | 0.07 | 0.05 | -0.08 | 0.05 | 0.04 | 0.03 | Height |
| rs4973429 | 2 | 232377818 | 0.99 | G | T | 0.02 | 0.00 | 0.01 | 0.02 | 0.08 | 0.05 | -0.07 | 0.05 | 0.01 | 0.03 | Height |
| rs2679184 | 2 | 232779223 | 1.00 | T | C | 0.03 | 0.00 | -0.01 | 0.03 | -0.04 | 0.06 | -0.03 | 0.06 | 0.01 | 0.04 | Height |
| rs749052 | 2 | 232796610 | 0.99 | T | C | 0.05 | 0.01 | -0.02 | 0.05 | -0.06 | 0.10 | 0.06 | 0.10 | -0.03 | 0.06 | Height |
| rs3116168 | 2 | 232989831 | 1.00 | C | T | 0.02 | 0.00 | -0.08 | 0.03 | -0.07 | 0.05 | -0.07 | 0.06 | -0.09 | 0.04 | Height |
| rs2343240 | 2 | 233087483 | 0.99 | T | C | 0.06 | 0.01 | -0.07 | 0.09 | -0.11 | 0.17 | -0.09 | 0.18 | -0.04 | 0.12 | Height |
| rs13393800 | 2 | 233442091 | 0.99 | A | G | 0.02 | 0.00 | 0.00 | 0.03 | -0.04 | 0.05 | 0.01 | 0.06 | 0.01 | 0.04 | Height |
| rs4344931 | 2 | 241818527 | 0.99 | C | A | 0.02 | 0.00 | -0.04 | 0.03 | -0.01 | 0.05 | -0.10 | 0.05 | -0.04 | 0.04 | Height |
| rs2633761 | 3 | 4728104 | 0.99 | A | G | 0.02 | 0.00 | -0.01 | 0.02 | -0.04 | 0.05 | -0.05 | 0.05 | 0.03 | 0.03 | Height |
| rs13078528 | 3 | 11646954 | 1.00 | A | G | 0.05 | 0.01 | 0.09 | 0.05 | 0.00 | 0.11 | 0.20 | 0.11 | 0.07 | 0.07 | Height |
| rs2597513 | 3 | 13555836 | 1.00 | C | T | 0.04 | 0.00 | 0.00 | 0.04 | 0.10 | 0.08 | -0.12 | 0.08 | 0.01 | 0.05 | Height |
| rs11708412 | 3 | 13733624 | 0.95 | A | G | 0.02 | 0.00 | 0.00 | 0.03 | -0.04 | 0.06 | 0.06 | 0.06 | 0.00 | 0.04 | Height |
| rs3915129 | 3 | 41243742 | 0.99 | G | T | 0.02 | 0.00 | 0.02 | 0.02 | 0.05 | 0.05 | 0.03 | 0.05 | 0.00 | 0.03 | Height |
| rs13088462 | 3 | 51071713 | 1.00 | C | T | 0.05 | 0.01 | 0.02 | 0.05 | -0.01 | 0.11 | 0.11 | 0.12 | 0.01 | 0.07 | Height |
| rs4256170 | 3 | 51192126 | 0.91 | A | G | 0.16 | 0.03 | 0.07 | 0.08 | 0.27 | 0.17 | 0.16 | 0.18 | -0.05 | 0.11 | Height |
| rs2581830 | 3 | 53134098 | 1.00 | T | C | 0.02 | 0.00 | -0.04 | 0.02 | -0.07 | 0.05 | -0.02 | 0.05 | -0.04 | 0.03 | Height |
| rs2034172 | 3 | 55411763 | 0.99 | G | A | 0.02 | 0.00 | 0.03 | 0.03 | 0.02 | 0.05 | -0.03 | 0.05 | 0.06 | 0.04 | Height |
| rs1658351 | 3 | 58013573 | 0.99 | C | T | 0.02 | 0.00 | 0.01 | 0.03 | -0.05 | 0.05 | 0.11 | 0.05 | -0.01 | 0.04 | Height |
| rs6794009 | 3 | 61513495 | 1.00 | G | A | 0.02 | 0.00 | 0.02 | 0.02 | 0.03 | 0.05 | -0.11 | 0.05 | 0.08 | 0.03 | Height |
| rs17806888 | 3 | 67416322 | 0.99 | T | C | 0.03 | 0.00 | 0.07 | 0.04 | 0.11 | 0.08 | 0.04 | 0.08 | 0.06 | 0.05 | Height |
| rs2175513 | 3 | 68622366 | 0.99 | G | A | 0.02 | 0.00 | -0.02 | 0.02 | -0.03 | 0.05 | 0.00 | 0.05 | -0.03 | 0.03 | Height |
| rs12330322 | 3 | 72455355 | 1.00 | C | T | 0.03 | 0.00 | -0.04 | 0.03 | -0.09 | 0.06 | -0.02 | 0.06 | -0.02 | 0.04 | Height |
| rs7633464 | 3 | 98715823 | 1.00 | A | G | 0.02 | 0.00 | 0.04 | 0.02 | 0.08 | 0.05 | 0.04 | 0.05 | 0.02 | 0.03 | Height |
| rs1533269 | 3 | 114214611 | 0.99 | C | A | 0.02 | 0.00 | 0.00 | 0.03 | -0.10 | 0.05 | 0.05 | 0.05 | 0.03 | 0.04 | Height |
| rs6439168 | 3 | 129050943 | 1.00 | G | A | 0.04 | 0.00 | -0.05 | 0.03 | -0.02 | 0.06 | 0.00 | 0.06 | -0.08 | 0.04 | Height |
| rs6762606 | 3 | 134379752 | 0.99 | T | C | 0.02 | 0.00 | 0.00 | 0.03 | 0.04 | 0.05 | -0.01 | 0.06 | -0.02 | 0.04 | Height |
| rs9880211 | 3 | 136107549 | 1.00 | G | A | 0.03 | 0.00 | -0.01 | 0.03 | -0.02 | 0.06 | -0.04 | 0.06 | 0.01 | 0.04 | Height |
| rs724016 | 3 | 141105570 | 1.00 | G | A | 0.08 | 0.00 | 0.03 | 0.02 | 0.02 | 0.05 | 0.01 | 0.05 | 0.04 | 0.03 | Height |
| rs936339 | 3 | 142535505 | 1.00 | T | C | 0.02 | 0.00 | 0.01 | 0.03 | -0.05 | 0.06 | 0.00 | 0.06 | 0.04 | 0.04 | Height |
| rs4325879 | 3 | 156851984 | 0.99 | C | T | 0.02 | 0.00 | 0.01 | 0.03 | 0.10 | 0.05 | -0.06 | 0.06 | 0.00 | 0.04 | Height |
| rs6441170 | 3 | 157806960 | 1.00 | C | T | 0.02 | 0.00 | -0.01 | 0.02 | 0.00 | 0.05 | -0.03 | 0.05 | -0.01 | 0.03 | Height |
| rs509035 | 3 | 172163449 | 1.00 | A | G | 0.03 | 0.00 | 0.01 | 0.03 | 0.01 | 0.05 | 0.03 | 0.05 | 0.01 | 0.04 | Height |
| rs9858528 | 3 | 183355405 | 1.00 | A | G | 0.02 | 0.00 | 0.01 | 0.03 | -0.04 | 0.06 | 0.02 | 0.06 | 0.04 | 0.04 | Height |
| rs16860216 | 3 | 185488882 | 0.99 | G | A | 0.05 | 0.00 | 0.01 | 0.03 | -0.04 | 0.06 | 0.06 | 0.06 | 0.01 | 0.04 | Height |
| rs720390 | 3 | 185548683 | 0.98 | A | G | 0.07 | 0.00 | -0.01 | 0.02 | -0.02 | 0.05 | -0.06 | 0.05 | 0.01 | 0.03 | Height |
| rs2300921 | 3 | 185651001 | 1.00 | C | T | 0.02 | 0.00 | -0.01 | 0.02 | 0.07 | 0.05 | -0.02 | 0.05 | -0.04 | 0.03 | Height |
| rs4686904 | 3 | 187438522 | 0.99 | C | T | 0.02 | 0.00 | 0.02 | 0.03 | 0.05 | 0.05 | -0.07 | 0.05 | 0.05 | 0.03 | Height |
| rs7646824 | 3 | 190815978 | 0.99 | A | G | 0.03 | 0.00 | 0.08 | 0.04 | 0.05 | 0.08 | 0.09 | 0.08 | 0.09 | 0.05 | Height |
| rs9841435 | 3 | 191111160 | 1.00 | G | A | 0.02 | 0.00 | 0.04 | 0.03 | 0.07 | 0.05 | 0.08 | 0.05 | 0.01 | 0.03 | Height |
| rs3958122 | 4 | 1693931 | 0.97 | T | C | 0.03 | 0.00 | -0.03 | 0.03 | -0.10 | 0.05 | 0.08 | 0.05 | -0.05 | 0.03 | Height |
| rs11722554 | 4 | 5016883 | 0.99 | G | A | 0.06 | 0.01 | -0.08 | 0.06 | -0.05 | 0.12 | 0.09 | 0.13 | -0.16 | 0.08 | Height |
| rs6446315 | 4 | 5035587 | 0.98 | G | A | 0.03 | 0.00 | -0.03 | 0.03 | 0.06 | 0.06 | -0.06 | 0.07 | -0.05 | 0.04 | Height |
| rs868489 | 4 | 7055253 | 0.97 | C | T | 0.02 | 0.00 | 0.01 | 0.03 | 0.01 | 0.06 | -0.04 | 0.06 | 0.03 | 0.04 | Height |
| rs6829680 | 4 | 7912333 | 1.00 | A | G | 0.02 | 0.00 | -0.02 | 0.02 | -0.06 | 0.05 | -0.03 | 0.05 | 0.00 | 0.03 | Height |
| rs2302580 | 4 | 8608634 | 0.98 | C | T | 0.03 | 0.00 | -0.02 | 0.02 | 0.01 | 0.05 | -0.05 | 0.05 | -0.02 | 0.03 | Height |
| rs763318 | 4 | 12963574 | 1.00 | G | A | 0.02 | 0.00 | 0.00 | 0.02 | -0.04 | 0.05 | 0.02 | 0.05 | 0.01 | 0.03 | Height |
| rs4834927 | 4 | 13194091 | 1.00 | G | A | 0.02 | 0.00 | 0.00 | 0.02 | -0.02 | 0.05 | -0.01 | 0.05 | 0.02 | 0.03 | Height |
| rs7692995 | 4 | 17936634 | 1.00 | T | C | 0.10 | 0.00 | 0.03 | 0.03 | 0.09 | 0.07 | -0.02 | 0.07 | 0.03 | 0.04 | Height |
| rs961014 | 4 | 18010384 | 0.97 | A | G | 0.04 | 0.00 | -0.01 | 0.02 | 0.02 | 0.05 | -0.03 | 0.05 | -0.01 | 0.03 | Height |
| rs16994718 | 4 | 38688362 | 1.00 | C | T | 0.03 | 0.00 | 0.07 | 0.03 | 0.16 | 0.07 | 0.03 | 0.07 | 0.05 | 0.05 | Height |
| rs2306596 | 4 | 39343940 | 1.00 | A | C | 0.02 | 0.00 | -0.01 | 0.02 | -0.02 | 0.05 | 0.04 | 0.05 | -0.03 | 0.03 | Height |
| rs1996422 | 4 | 48687351 | 0.99 | G | A | 0.02 | 0.00 | 0.00 | 0.03 | 0.07 | 0.05 | 0.00 | 0.06 | -0.03 | 0.04 | Height |
| rs13113518 | 4 | 56399648 | 0.99 | C | T | 0.02 | 0.00 | -0.04 | 0.02 | -0.14 | 0.05 | 0.00 | 0.05 | -0.02 | 0.03 | Height |
| rs17081935 | 4 | 57823476 | 1.00 | T | C | 0.03 | 0.00 | -0.02 | 0.03 | -0.13 | 0.06 | 0.07 | 0.06 | 0.00 | 0.04 | Height |
| rs9993613 | 4 | 73476014 | 0.99 | T | G | 0.03 | 0.00 | 0.02 | 0.02 | 0.05 | 0.05 | -0.01 | 0.05 | 0.01 | 0.03 | Height |
| rs17556750 | 4 | 82155568 | 0.98 | A | C | 0.04 | 0.00 | 0.02 | 0.03 | 0.00 | 0.05 | 0.09 | 0.05 | 0.00 | 0.04 | Height |
| rs17499117 | 4 | 82204091 | 0.70 | G | A | 0.13 | 0.02 | 0.02 | 0.07 | 0.01 | 0.12 | 0.06 | 0.15 | 0.01 | 0.09 | Height |
| rs11731978 | 4 | 88194170 | 1.00 | A | G | 0.02 | 0.00 | -0.06 | 0.03 | -0.06 | 0.07 | -0.03 | 0.07 | -0.07 | 0.05 | Height |
| rs12639764 | 4 | 106216205 | 1.00 | T | C | 0.03 | 0.00 | 0.04 | 0.02 | 0.04 | 0.05 | -0.01 | 0.05 | 0.07 | 0.03 | Height |
| rs7659107 | 4 | 114742249 | 1.00 | G | A | 0.02 | 0.00 | -0.02 | 0.03 | -0.04 | 0.06 | 0.11 | 0.06 | -0.06 | 0.04 | Height |
| rs6838153 | 4 | 122720999 | 1.00 | G | A | 0.02 | 0.00 | -0.03 | 0.03 | 0.02 | 0.05 | -0.11 | 0.05 | -0.02 | 0.03 | Height |
| rs12513181 | 4 | 123835656 | 1.00 | C | A | 0.02 | 0.00 | -0.03 | 0.03 | 0.01 | 0.05 | -0.05 | 0.06 | -0.04 | 0.04 | Height |
| rs11100790 | 4 | 144442611 | 1.00 | C | T | 0.02 | 0.00 | 0.02 | 0.03 | 0.03 | 0.06 | 0.01 | 0.06 | 0.02 | 0.04 | Height |
| rs7654571 | 4 | 145321006 | 0.98 | A | G | 0.02 | 0.00 | -0.04 | 0.03 | 0.00 | 0.06 | -0.06 | 0.06 | -0.05 | 0.04 | Height |
| rs6845999 | 4 | 145565826 | 1.00 | T | C | 0.02 | 0.00 | 0.02 | 0.02 | 0.03 | 0.05 | 0.06 | 0.05 | 0.01 | 0.03 | Height |
| rs1812175 | 4 | 145574844 | 1.00 | G | A | 0.05 | 0.00 | 0.02 | 0.03 | 0.09 | 0.06 | 0.05 | 0.07 | -0.03 | 0.04 | Height |
| rs17777628 | 4 | 145592686 | 0.99 | G | A | 0.04 | 0.01 | -0.05 | 0.06 | -0.17 | 0.12 | -0.10 | 0.12 | 0.05 | 0.09 | Height |
| rs4240326 | 4 | 145839264 | 1.00 | A | G | 0.02 | 0.00 | 0.01 | 0.02 | 0.01 | 0.05 | -0.06 | 0.05 | 0.05 | 0.03 | Height |
| rs996743 | 4 | 146128884 | 0.84 | A | G | 0.04 | 0.01 | -0.01 | 0.05 | 0.07 | 0.08 | -0.06 | 0.10 | -0.03 | 0.06 | Height |
| rs13150868 | 4 | 152180671 | 1.00 | T | G | 0.02 | 0.00 | -0.01 | 0.02 | 0.04 | 0.05 | -0.04 | 0.05 | -0.01 | 0.03 | Height |
| rs955748 | 4 | 184215675 | 1.00 | G | A | 0.03 | 0.00 | 0.00 | 0.03 | 0.06 | 0.06 | -0.08 | 0.06 | 0.01 | 0.04 | Height |
| rs17410035 | 5 | 31541142 | 1.00 | T | G | 0.02 | 0.00 | 0.01 | 0.03 | 0.05 | 0.05 | -0.05 | 0.05 | 0.02 | 0.03 | Height |
| rs7731703 | 5 | 32694942 | 1.00 | C | T | 0.03 | 0.00 | 0.05 | 0.03 | 0.02 | 0.05 | 0.06 | 0.05 | 0.05 | 0.04 | Height |
| rs3811958 | 5 | 32772043 | 1.00 | G | A | 0.05 | 0.00 | 0.00 | 0.03 | 0.05 | 0.05 | -0.09 | 0.06 | 0.01 | 0.04 | Height |
| rs9292468 | 5 | 32819073 | 0.99 | T | C | 0.05 | 0.00 | 0.00 | 0.02 | 0.01 | 0.05 | -0.06 | 0.05 | 0.02 | 0.03 | Height |
| rs13183624 | 5 | 32821168 | 0.99 | G | A | 0.03 | 0.00 | 0.01 | 0.03 | -0.03 | 0.06 | 0.12 | 0.06 | -0.03 | 0.04 | Height |
| rs11745439 | 5 | 33230034 | 1.00 | G | A | 0.02 | 0.00 | -0.03 | 0.03 | -0.04 | 0.05 | 0.03 | 0.05 | -0.05 | 0.04 | Height |
| rs301901 | 5 | 37046626 | 1.00 | A | G | 0.03 | 0.00 | 0.05 | 0.02 | -0.01 | 0.05 | 0.06 | 0.05 | 0.06 | 0.03 | Height |
| rs3812040 | 5 | 39426020 | 0.99 | T | C | 0.02 | 0.00 | 0.04 | 0.03 | 0.00 | 0.05 | 0.05 | 0.06 | 0.04 | 0.04 | Height |
| rs17574650 | 5 | 42436916 | 0.87 | C | A | 0.04 | 0.01 | 0.02 | 0.04 | 0.01 | 0.08 | 0.06 | 0.09 | 0.00 | 0.06 | Height |
| rs7716219 | 5 | 54955071 | 1.00 | T | C | 0.03 | 0.00 | 0.04 | 0.03 | 0.00 | 0.05 | 0.11 | 0.05 | 0.03 | 0.04 | Height |
| rs2662027 | 5 | 56254485 | 1.00 | G | T | 0.03 | 0.00 | -0.05 | 0.04 | 0.01 | 0.08 | 0.01 | 0.08 | -0.10 | 0.05 | Height |
| rs7727731 | 5 | 64674446 | 1.00 | T | C | 0.03 | 0.00 | -0.01 | 0.04 | 0.02 | 0.08 | -0.11 | 0.08 | 0.03 | 0.05 | Height |
| rs9291926 | 5 | 67599656 | 1.00 | T | G | 0.02 | 0.00 | 0.01 | 0.02 | 0.00 | 0.05 | 0.01 | 0.05 | 0.01 | 0.03 | Height |
| rs34651 | 5 | 72144005 | 0.94 | C | T | 0.04 | 0.01 | -0.04 | 0.05 | -0.01 | 0.09 | -0.14 | 0.10 | -0.02 | 0.06 | Height |
| rs820848 | 5 | 73964660 | 0.98 | G | A | 0.02 | 0.00 | 0.01 | 0.03 | 0.06 | 0.05 | -0.03 | 0.06 | -0.01 | 0.04 | Height |
| rs12519505 | 5 | 77505876 | 1.00 | C | T | 0.02 | 0.00 | -0.01 | 0.03 | -0.02 | 0.06 | 0.03 | 0.06 | -0.02 | 0.04 | Height |
| rs7712162 | 5 | 78945171 | 1.00 | T | C | 0.02 | 0.00 | -0.08 | 0.03 | -0.10 | 0.06 | 0.01 | 0.07 | -0.10 | 0.04 | Height |
| rs32855 | 5 | 79836192 | 0.99 | A | G | 0.02 | 0.00 | 0.04 | 0.03 | 0.05 | 0.06 | 0.06 | 0.06 | 0.03 | 0.04 | Height |
| rs6894139 | 5 | 88327782 | 1.00 | T | G | 0.03 | 0.00 | -0.02 | 0.02 | -0.02 | 0.05 | 0.02 | 0.05 | -0.05 | 0.03 | Height |
| rs2247870 | 5 | 90151589 | 1.00 | A | G | 0.02 | 0.00 | -0.03 | 0.02 | -0.10 | 0.05 | -0.04 | 0.05 | 0.00 | 0.03 | Height |
| rs6594336 | 5 | 108073085 | 1.00 | C | T | 0.02 | 0.00 | 0.02 | 0.02 | 0.07 | 0.05 | 0.00 | 0.05 | 0.01 | 0.03 | Height |
| rs13177718 | 5 | 108113344 | 1.00 | C | T | 0.05 | 0.01 | 0.06 | 0.05 | 0.01 | 0.10 | 0.23 | 0.10 | 0.01 | 0.06 | Height |
| rs1582931 | 5 | 122657199 | 0.97 | G | A | 0.03 | 0.00 | 0.06 | 0.02 | 0.12 | 0.05 | 0.08 | 0.05 | 0.03 | 0.03 | Height |
| rs26024 | 5 | 127696022 | 1.00 | C | A | 0.02 | 0.00 | -0.02 | 0.03 | 0.04 | 0.05 | -0.11 | 0.05 | -0.02 | 0.03 | Height |
| rs7701414 | 5 | 131585958 | 1.00 | G | A | 0.04 | 0.00 | 0.01 | 0.02 | 0.01 | 0.05 | 0.01 | 0.05 | 0.01 | 0.03 | Height |
| rs526896 | 5 | 134356705 | 0.97 | T | G | 0.04 | 0.00 | -0.01 | 0.03 | 0.00 | 0.05 | -0.01 | 0.06 | -0.01 | 0.04 | Height |
| rs9327705 | 5 | 134510303 | 1.00 | A | G | 0.02 | 0.00 | -0.03 | 0.03 | -0.06 | 0.06 | 0.02 | 0.06 | -0.03 | 0.04 | Height |
| rs165189 | 5 | 139145747 | 0.97 | G | A | 0.03 | 0.00 | 0.03 | 0.03 | 0.04 | 0.07 | -0.03 | 0.07 | 0.06 | 0.05 | Height |
| rs4624820 | 5 | 141681788 | 1.00 | A | G | 0.02 | 0.00 | -0.03 | 0.02 | 0.00 | 0.05 | 0.00 | 0.05 | -0.05 | 0.03 | Height |
| rs2974438 | 5 | 168250903 | 0.99 | G | A | 0.04 | 0.00 | 0.03 | 0.03 | 0.03 | 0.06 | 0.03 | 0.06 | 0.02 | 0.04 | Height |
| rs4620037 | 5 | 170875097 | 1.00 | A | C | 0.03 | 0.00 | -0.03 | 0.03 | -0.06 | 0.06 | -0.03 | 0.06 | -0.01 | 0.04 | Height |
| rs1529701 | 5 | 171000977 | 0.99 | C | T | 0.02 | 0.00 | 0.04 | 0.03 | 0.02 | 0.05 | 0.01 | 0.05 | 0.05 | 0.04 | Height |
| rs33852 | 5 | 171189571 | 1.00 | G | A | 0.02 | 0.00 | -0.01 | 0.03 | 0.00 | 0.05 | -0.03 | 0.05 | 0.00 | 0.03 | Height |
| rs12153391 | 5 | 171203438 | 0.96 | C | A | 0.02 | 0.00 | -0.01 | 0.03 | 0.04 | 0.06 | 0.03 | 0.06 | -0.05 | 0.04 | Height |
| rs4868126 | 5 | 171283469 | 1.00 | G | T | 0.03 | 0.00 | 0.01 | 0.02 | -0.01 | 0.05 | -0.01 | 0.05 | 0.02 | 0.03 | Height |
| rs1368380 | 5 | 171285632 | 0.96 | T | C | 0.02 | 0.00 | 0.03 | 0.03 | -0.01 | 0.05 | -0.01 | 0.05 | 0.07 | 0.04 | Height |
| rs17075869 | 5 | 172811280 | 0.98 | T | C | 0.03 | 0.01 | -0.02 | 0.04 | -0.13 | 0.09 | 0.13 | 0.09 | -0.03 | 0.06 | Height |
| rs7733195 | 5 | 172994624 | 0.99 | G | A | 0.03 | 0.00 | 0.00 | 0.02 | -0.08 | 0.05 | 0.04 | 0.05 | 0.02 | 0.03 | Height |
| rs4868645 | 5 | 175947118 | 1.00 | G | T | 0.02 | 0.00 | 0.04 | 0.02 | 0.06 | 0.05 | 0.03 | 0.05 | 0.03 | 0.03 | Height |
| rs422421 | 5 | 176517326 | 0.98 | C | T | 0.03 | 0.00 | 0.04 | 0.03 | 0.07 | 0.06 | 0.08 | 0.06 | 0.01 | 0.04 | Height |
| rs12055154 | 5 | 176675423 | 0.99 | A | G | 0.06 | 0.01 | 0.04 | 0.08 | -0.10 | 0.16 | -0.01 | 0.16 | 0.13 | 0.11 | Height |
| rs11750568 | 5 | 178535713 | 1.00 | A | G | 0.02 | 0.00 | 0.04 | 0.03 | 0.08 | 0.05 | -0.05 | 0.05 | 0.06 | 0.03 | Height |
| rs6879260 | 5 | 179731014 | 0.99 | C | T | 0.03 | 0.00 | 0.00 | 0.02 | -0.01 | 0.05 | 0.00 | 0.05 | 0.00 | 0.03 | Height |
| rs932445 | 6 | 2167225 | 0.97 | T | C | 0.02 | 0.00 | 0.00 | 0.02 | -0.02 | 0.05 | 0.01 | 0.05 | 0.01 | 0.03 | Height |
| rs4246079 | 6 | 6889818 | 0.99 | G | A | 0.04 | 0.01 | 0.02 | 0.04 | 0.00 | 0.07 | -0.12 | 0.07 | 0.09 | 0.05 | Height |
| rs17603945 | 6 | 7213016 | 1.00 | A | G | 0.03 | 0.00 | -0.01 | 0.03 | 0.09 | 0.06 | 0.08 | 0.07 | -0.09 | 0.04 | Height |
| rs9392918 | 6 | 7708631 | 1.00 | C | T | 0.04 | 0.00 | -0.01 | 0.02 | 0.00 | 0.05 | 0.01 | 0.05 | -0.03 | 0.03 | Height |
| rs9328445 | 6 | 7792947 | 1.00 | C | T | 0.04 | 0.00 | 0.04 | 0.02 | 0.03 | 0.05 | 0.07 | 0.05 | 0.03 | 0.03 | Height |
| rs9405356 | 6 | 7804377 | 0.97 | T | C | 0.02 | 0.00 | -0.03 | 0.02 | -0.08 | 0.05 | -0.04 | 0.05 | 0.00 | 0.03 | Height |
| rs17330192 | 6 | 17589375 | 0.99 | C | T | 0.02 | 0.00 | -0.04 | 0.03 | 0.04 | 0.05 | 0.00 | 0.06 | -0.11 | 0.04 | Height |
| rs1047014 | 6 | 19841493 | 1.00 | C | T | 0.03 | 0.00 | 0.01 | 0.03 | 0.04 | 0.06 | 0.01 | 0.06 | -0.01 | 0.04 | Height |
| rs806794 | 6 | 26200677 | 1.00 | A | G | 0.05 | 0.00 | 0.02 | 0.03 | 0.01 | 0.05 | -0.04 | 0.05 | 0.05 | 0.04 | Height |
| rs1265097 | 6 | 31106459 | 1.00 | C | A | 0.04 | 0.01 | -0.01 | 0.04 | 0.17 | 0.08 | -0.03 | 0.08 | -0.08 | 0.05 | Height |
| rs12204421 | 6 | 33628863 | 1.00 | A | G | 0.03 | 0.00 | 0.04 | 0.03 | 0.00 | 0.05 | 0.08 | 0.06 | 0.04 | 0.04 | Height |
| rs3957165 | 6 | 33784864 | 1.00 | T | C | 0.03 | 0.00 | -0.05 | 0.03 | -0.05 | 0.06 | -0.06 | 0.06 | -0.05 | 0.04 | Height |
| rs12214804 | 6 | 34188866 | 0.99 | C | T | 0.09 | 0.01 | 0.02 | 0.04 | 0.04 | 0.09 | 0.06 | 0.08 | -0.01 | 0.06 | Height |
| rs6919534 | 6 | 35246903 | 1.00 | A | G | 0.03 | 0.00 | 0.01 | 0.04 | -0.03 | 0.07 | -0.03 | 0.07 | 0.05 | 0.05 | Height |
| rs6899744 | 6 | 35286295 | 1.00 | G | T | 0.10 | 0.01 | -0.09 | 0.10 | 0.04 | 0.20 | -0.17 | 0.18 | -0.11 | 0.14 | Height |
| rs4713902 | 6 | 35614026 | 0.99 | T | C | 0.02 | 0.00 | -0.02 | 0.03 | 0.05 | 0.05 | -0.04 | 0.06 | -0.05 | 0.04 | Height |
| rs16895130 | 6 | 41924931 | 1.00 | G | A | 0.03 | 0.00 | 0.02 | 0.03 | 0.05 | 0.05 | 0.05 | 0.06 | 0.00 | 0.04 | Height |
| rs1040941 | 6 | 44539761 | 0.99 | A | G | 0.02 | 0.00 | -0.01 | 0.02 | 0.05 | 0.05 | 0.00 | 0.05 | -0.04 | 0.03 | Height |
| rs10948222 | 6 | 45244415 | 1.00 | C | T | 0.03 | 0.00 | 0.01 | 0.02 | 0.03 | 0.05 | -0.01 | 0.05 | 0.01 | 0.03 | Height |
| rs9395264 | 6 | 47475022 | 0.99 | G | T | 0.02 | 0.00 | -0.01 | 0.03 | 0.00 | 0.05 | -0.02 | 0.05 | -0.01 | 0.04 | Height |
| rs12209223 | 6 | 76164589 | 0.99 | A | C | 0.05 | 0.00 | 0.01 | 0.04 | 0.05 | 0.08 | -0.18 | 0.08 | 0.07 | 0.06 | Height |
| rs6903448 | 6 | 76173832 | 1.00 | C | T | 0.03 | 0.00 | 0.01 | 0.03 | -0.04 | 0.07 | 0.11 | 0.07 | -0.01 | 0.05 | Height |
| rs648831 | 6 | 80956208 | 1.00 | T | C | 0.03 | 0.00 | 0.05 | 0.02 | 0.14 | 0.05 | 0.06 | 0.05 | 0.00 | 0.03 | Height |
| rs1341278 | 6 | 81038921 | 1.00 | G | T | 0.04 | 0.01 | -0.06 | 0.05 | 0.01 | 0.10 | -0.07 | 0.11 | -0.08 | 0.07 | Height |
| rs9443804 | 6 | 81315597 | 1.00 | G | A | 0.02 | 0.00 | 0.06 | 0.02 | 0.03 | 0.05 | 0.04 | 0.05 | 0.08 | 0.03 | Height |
| rs310421 | 6 | 81792063 | 1.00 | T | G | 0.03 | 0.00 | -0.03 | 0.02 | -0.12 | 0.05 | 0.08 | 0.05 | -0.02 | 0.03 | Height |
| rs3828760 | 6 | 82456984 | 1.00 | T | C | 0.03 | 0.00 | 0.02 | 0.04 | 0.07 | 0.07 | -0.02 | 0.08 | 0.02 | 0.05 | Height |
| rs761391 | 6 | 85448103 | 1.00 | C | T | 0.02 | 0.00 | 0.03 | 0.02 | 0.08 | 0.05 | 0.02 | 0.05 | 0.01 | 0.03 | Height |
| rs314263 | 6 | 105392745 | 1.00 | C | T | 0.04 | 0.00 | 0.02 | 0.03 | 0.05 | 0.05 | 0.00 | 0.05 | 0.02 | 0.04 | Height |
| rs479744 | 6 | 109020032 | 0.98 | G | T | 0.02 | 0.00 | 0.05 | 0.03 | 0.06 | 0.06 | -0.05 | 0.06 | 0.08 | 0.04 | Height |
| rs6920372 | 6 | 109723939 | 1.00 | G | A | 0.03 | 0.00 | 0.02 | 0.02 | 0.00 | 0.05 | 0.00 | 0.05 | 0.03 | 0.03 | Height |
| rs2145357 | 6 | 116451442 | 1.00 | G | A | 0.02 | 0.00 | 0.00 | 0.03 | 0.04 | 0.05 | 0.03 | 0.05 | -0.04 | 0.04 | Height |
| rs1405212 | 6 | 117490664 | 1.00 | C | T | 0.02 | 0.00 | 0.01 | 0.02 | 0.02 | 0.05 | -0.01 | 0.05 | 0.01 | 0.03 | Height |
| rs389663 | 6 | 117868051 | 1.00 | T | C | 0.02 | 0.00 | -0.03 | 0.03 | 0.03 | 0.05 | -0.01 | 0.05 | -0.06 | 0.03 | Height |
| rs1155939 | 6 | 126866133 | 0.99 | A | C | 0.04 | 0.00 | -0.08 | 0.02 | -0.04 | 0.05 | -0.12 | 0.05 | -0.08 | 0.03 | Height |
| rs1415701 | 6 | 130345835 | 0.96 | G | A | 0.03 | 0.00 | -0.05 | 0.03 | -0.03 | 0.05 | -0.08 | 0.06 | -0.05 | 0.04 | Height |
| rs6921207 | 6 | 131327956 | 1.00 | A | G | 0.02 | 0.00 | -0.03 | 0.02 | -0.01 | 0.05 | 0.00 | 0.05 | -0.06 | 0.03 | Height |
| rs7745166 | 6 | 142617680 | 0.95 | C | A | 0.03 | 0.00 | -0.01 | 0.02 | -0.03 | 0.05 | -0.04 | 0.05 | 0.02 | 0.03 | Height |
| rs4896582 | 6 | 142703877 | 0.99 | G | A | 0.05 | 0.00 | -0.01 | 0.03 | -0.01 | 0.05 | 0.06 | 0.05 | -0.04 | 0.04 | Height |
| rs6911389 | 6 | 144079629 | 1.00 | T | G | 0.02 | 0.00 | -0.02 | 0.02 | -0.06 | 0.05 | 0.03 | 0.05 | -0.02 | 0.03 | Height |
| rs6902771 | 6 | 152157881 | 1.00 | T | C | 0.03 | 0.00 | -0.02 | 0.02 | -0.07 | 0.05 | -0.01 | 0.05 | -0.01 | 0.03 | Height |
| rs3020418 | 6 | 152345162 | 1.00 | A | G | 0.03 | 0.00 | 0.00 | 0.03 | 0.04 | 0.05 | -0.07 | 0.05 | 0.01 | 0.04 | Height |
| rs11156098 | 6 | 156587831 | 0.98 | T | C | 0.03 | 0.00 | 0.04 | 0.04 | 0.10 | 0.08 | -0.08 | 0.08 | 0.07 | 0.05 | Height |
| rs1832871 | 6 | 158722034 | 1.00 | A | G | 0.02 | 0.00 | 0.00 | 0.03 | 0.09 | 0.05 | -0.06 | 0.05 | -0.02 | 0.03 | Height |
| rs991946 | 6 | 166329862 | 0.99 | C | T | 0.02 | 0.00 | 0.03 | 0.02 | 0.00 | 0.05 | 0.02 | 0.05 | 0.06 | 0.03 | Height |
| rs2763273 | 6 | 168834623 | 0.99 | C | T | 0.02 | 0.00 | -0.01 | 0.03 | -0.04 | 0.06 | 0.02 | 0.06 | -0.01 | 0.04 | Height |
| rs7774834 | 6 | 169349731 | 1.00 | A | C | 0.02 | 0.00 | 0.02 | 0.02 | 0.01 | 0.05 | -0.09 | 0.05 | 0.07 | 0.03 | Height |
| rs798497 | 7 | 2795957 | 1.00 | A | G | 0.06 | 0.00 | 0.07 | 0.03 | 0.03 | 0.05 | 0.05 | 0.06 | 0.10 | 0.04 | Height |
| rs4725061 | 7 | 8086639 | 0.99 | G | A | 0.02 | 0.00 | -0.03 | 0.02 | -0.01 | 0.05 | -0.02 | 0.05 | -0.04 | 0.03 | Height |
| rs929637 | 7 | 12276522 | 1.00 | G | T | 0.02 | 0.00 | -0.01 | 0.03 | 0.03 | 0.06 | -0.06 | 0.06 | 0.00 | 0.04 | Height |
| rs17140875 | 7 | 19248278 | 0.98 | G | T | 0.04 | 0.01 | -0.04 | 0.06 | -0.08 | 0.11 | 0.22 | 0.12 | -0.13 | 0.08 | Height |
| rs2390151 | 7 | 19642100 | 1.00 | T | G | 0.03 | 0.00 | -0.05 | 0.03 | -0.09 | 0.06 | -0.06 | 0.06 | -0.02 | 0.04 | Height |
| rs3807931 | 7 | 20381674 | 1.00 | A | G | 0.03 | 0.00 | 0.04 | 0.02 | 0.07 | 0.05 | 0.07 | 0.05 | 0.01 | 0.03 | Height |
| rs7782764 | 7 | 23475919 | 0.99 | A | G | 0.03 | 0.00 | 0.02 | 0.04 | 0.05 | 0.07 | -0.04 | 0.07 | 0.03 | 0.05 | Height |
| rs12538407 | 7 | 23521316 | 1.00 | A | G | 0.04 | 0.00 | -0.03 | 0.02 | -0.04 | 0.05 | 0.07 | 0.05 | -0.06 | 0.03 | Height |
| rs1055144 | 7 | 25871109 | 1.00 | T | C | 0.02 | 0.00 | 0.00 | 0.03 | 0.02 | 0.06 | -0.07 | 0.06 | 0.02 | 0.04 | Height |
| rs552707 | 7 | 28205303 | 0.97 | T | C | 0.05 | 0.00 | -0.04 | 0.03 | -0.09 | 0.05 | -0.14 | 0.06 | 0.01 | 0.04 | Height |
| rs12533079 | 7 | 28751137 | 1.00 | T | G | 0.02 | 0.00 | 0.05 | 0.03 | 0.10 | 0.06 | -0.03 | 0.06 | 0.07 | 0.04 | Height |
| rs6462432 | 7 | 32935524 | 1.00 | A | G | 0.02 | 0.00 | -0.03 | 0.02 | -0.12 | 0.05 | -0.04 | 0.05 | 0.02 | 0.03 | Height |
| rs1007358 | 7 | 46201355 | 1.00 | G | A | 0.02 | 0.00 | -0.02 | 0.03 | 0.08 | 0.06 | -0.01 | 0.06 | -0.07 | 0.04 | Height |
| rs2715094 | 7 | 50730452 | 1.00 | G | A | 0.02 | 0.00 | -0.01 | 0.03 | -0.02 | 0.06 | -0.01 | 0.06 | 0.00 | 0.04 | Height |
| rs1113765 | 7 | 55889334 | 1.00 | G | A | 0.02 | 0.00 | -0.03 | 0.03 | 0.08 | 0.06 | -0.08 | 0.06 | -0.05 | 0.04 | Height |
| rs12669267 | 7 | 73304636 | 1.00 | C | T | 0.03 | 0.01 | 0.01 | 0.03 | 0.03 | 0.07 | -0.03 | 0.07 | 0.01 | 0.05 | Height |
| rs17807185 | 7 | 77308295 | 1.00 | G | A | 0.02 | 0.00 | -0.02 | 0.02 | 0.02 | 0.05 | -0.02 | 0.05 | -0.04 | 0.03 | Height |
| rs2888877 | 7 | 92228400 | 0.98 | T | C | 0.04 | 0.00 | 0.05 | 0.03 | 0.02 | 0.06 | 0.17 | 0.06 | 0.02 | 0.04 | Height |
| rs42039 | 7 | 92244422 | 0.98 | T | C | 0.05 | 0.00 | 0.00 | 0.03 | -0.03 | 0.06 | 0.06 | 0.06 | -0.01 | 0.04 | Height |
| rs2188177 | 7 | 92659160 | 0.98 | T | C | 0.02 | 0.00 | -0.01 | 0.02 | -0.11 | 0.05 | -0.01 | 0.05 | 0.05 | 0.03 | Height |
| rs17250196 | 7 | 99817196 | 0.95 | T | G | 0.04 | 0.01 | -0.08 | 0.06 | -0.07 | 0.11 | 0.03 | 0.12 | -0.12 | 0.08 | Height |
| rs6952113 | 7 | 120777619 | 1.00 | G | A | 0.02 | 0.00 | 0.00 | 0.02 | 0.02 | 0.05 | -0.01 | 0.05 | 0.00 | 0.03 | Height |
| rs6962887 | 7 | 135045786 | 0.96 | T | G | 0.02 | 0.00 | 0.06 | 0.03 | 0.00 | 0.05 | 0.16 | 0.05 | 0.05 | 0.04 | Height |
| rs273945 | 7 | 137611566 | 1.00 | C | A | 0.02 | 0.00 | 0.01 | 0.02 | 0.00 | 0.05 | 0.07 | 0.05 | -0.01 | 0.03 | Height |
| rs822531 | 7 | 148629759 | 1.00 | T | C | 0.04 | 0.00 | 0.05 | 0.03 | 0.11 | 0.06 | 0.06 | 0.06 | 0.02 | 0.04 | Height |
| rs6955948 | 7 | 150508720 | 0.99 | T | C | 0.03 | 0.00 | 0.00 | 0.03 | -0.08 | 0.05 | 0.02 | 0.05 | 0.03 | 0.04 | Height |
| rs429433 | 8 | 8747894 | 1.00 | A | G | 0.05 | 0.01 | 0.05 | 0.06 | 0.11 | 0.12 | 0.13 | 0.12 | -0.01 | 0.08 | Height |
| rs7834383 | 8 | 13273477 | 1.00 | T | G | 0.02 | 0.00 | -0.01 | 0.03 | -0.03 | 0.05 | -0.01 | 0.05 | 0.01 | 0.03 | Height |
| rs7823327 | 8 | 22562352 | 1.00 | T | G | 0.02 | 0.00 | 0.01 | 0.02 | 0.00 | 0.05 | 0.03 | 0.05 | 0.00 | 0.03 | Height |
| rs4273857 | 8 | 23173053 | 1.00 | A | G | 0.03 | 0.00 | -0.05 | 0.03 | 0.02 | 0.06 | -0.11 | 0.06 | -0.05 | 0.04 | Height |
| rs2013265 | 8 | 24092500 | 1.00 | C | T | 0.03 | 0.00 | 0.00 | 0.03 | 0.00 | 0.05 | 0.04 | 0.06 | -0.01 | 0.04 | Height |
| rs568610 | 8 | 27527995 | 0.99 | T | C | 0.02 | 0.00 | 0.03 | 0.03 | 0.07 | 0.06 | -0.01 | 0.06 | 0.02 | 0.04 | Height |
| rs6988484 | 8 | 49413780 | 1.00 | C | T | 0.02 | 0.00 | 0.00 | 0.03 | -0.06 | 0.05 | 0.04 | 0.06 | 0.02 | 0.04 | Height |
| rs6999671 | 8 | 56998480 | 1.00 | A | G | 0.05 | 0.01 | -0.02 | 0.06 | -0.13 | 0.13 | 0.04 | 0.13 | 0.01 | 0.09 | Height |
| rs10958476 | 8 | 57095808 | 1.00 | C | T | 0.04 | 0.00 | -0.02 | 0.03 | -0.01 | 0.06 | -0.02 | 0.06 | -0.03 | 0.04 | Height |
| rs9650315 | 8 | 57155598 | 0.97 | G | T | 0.06 | 0.00 | 0.01 | 0.03 | 0.13 | 0.07 | 0.01 | 0.07 | -0.04 | 0.05 | Height |
| rs2956605 | 8 | 75883054 | 0.99 | A | C | 0.03 | 0.00 | 0.00 | 0.02 | -0.03 | 0.05 | 0.08 | 0.05 | -0.02 | 0.03 | Height |
| rs16939034 | 8 | 76040583 | 1.00 | T | C | 0.04 | 0.01 | 0.05 | 0.04 | 0.18 | 0.09 | -0.06 | 0.09 | 0.03 | 0.06 | Height |
| rs2737220 | 8 | 116637685 | 0.99 | T | C | 0.02 | 0.00 | 0.00 | 0.02 | 0.06 | 0.05 | -0.07 | 0.05 | 0.00 | 0.03 | Height |
| rs1550162 | 8 | 117563532 | 1.00 | G | A | 0.02 | 0.00 | 0.02 | 0.03 | -0.03 | 0.05 | 0.03 | 0.06 | 0.04 | 0.04 | Height |
| rs1599473 | 8 | 120475358 | 0.98 | G | T | 0.03 | 0.00 | -0.04 | 0.03 | -0.12 | 0.06 | -0.01 | 0.06 | -0.01 | 0.04 | Height |
| rs10283100 | 8 | 120596023 | 0.84 | G | A | 0.05 | 0.01 | 0.09 | 0.05 | 0.13 | 0.11 | 0.16 | 0.13 | 0.06 | 0.07 | Height |
| rs11779459 | 8 | 123980551 | 1.00 | T | C | 0.02 | 0.00 | -0.03 | 0.02 | -0.05 | 0.05 | -0.01 | 0.05 | -0.03 | 0.03 | Height |
| rs4733724 | 8 | 130723728 | 1.00 | A | G | 0.05 | 0.00 | -0.03 | 0.03 | 0.16 | 0.06 | -0.03 | 0.06 | -0.12 | 0.04 | Height |
| rs894343 | 8 | 135612595 | 0.99 | A | G | 0.03 | 0.00 | -0.03 | 0.03 | 0.03 | 0.05 | -0.07 | 0.05 | -0.04 | 0.04 | Height |
| rs1036821 | 8 | 135650483 | 0.99 | G | A | 0.05 | 0.00 | 0.01 | 0.03 | 0.00 | 0.05 | 0.02 | 0.05 | 0.02 | 0.04 | Height |
| rs6577717 | 8 | 135653832 | 1.00 | T | G | 0.04 | 0.00 | 0.00 | 0.03 | 0.01 | 0.05 | 0.05 | 0.05 | -0.03 | 0.03 | Height |
| rs3927536 | 9 | 16787670 | 1.00 | T | C | 0.02 | 0.00 | 0.03 | 0.03 | 0.07 | 0.06 | -0.01 | 0.06 | 0.03 | 0.04 | Height |
| rs1576900 | 9 | 18629792 | 1.00 | G | A | 0.02 | 0.00 | 0.00 | 0.03 | 0.01 | 0.05 | 0.08 | 0.05 | -0.04 | 0.04 | Height |
| rs10972628 | 9 | 35937611 | 1.00 | G | A | 0.02 | 0.00 | -0.05 | 0.03 | 0.06 | 0.06 | -0.05 | 0.06 | -0.10 | 0.04 | Height |
| rs11144688 | 9 | 78542286 | 0.85 | G | A | 0.06 | 0.01 | -0.07 | 0.04 | -0.12 | 0.08 | -0.09 | 0.08 | -0.03 | 0.05 | Height |
| rs7853235 | 9 | 86660782 | 0.98 | T | C | 0.03 | 0.00 | 0.02 | 0.03 | 0.00 | 0.06 | -0.01 | 0.06 | 0.05 | 0.04 | Height |
| rs181338 | 9 | 89108161 | 1.00 | T | C | 0.03 | 0.00 | -0.02 | 0.02 | -0.09 | 0.05 | 0.03 | 0.05 | -0.02 | 0.03 | Height |
| rs1571892 | 9 | 94258836 | 1.00 | C | A | 0.02 | 0.00 | -0.05 | 0.03 | -0.02 | 0.05 | -0.02 | 0.06 | -0.07 | 0.04 | Height |
| rs7043114 | 9 | 95387983 | 1.00 | C | T | 0.03 | 0.00 | 0.02 | 0.02 | 0.04 | 0.05 | 0.09 | 0.05 | -0.02 | 0.03 | Height |
| rs1257763 | 9 | 96893945 | 0.96 | A | G | 0.07 | 0.01 | 0.12 | 0.06 | 0.20 | 0.12 | -0.03 | 0.13 | 0.14 | 0.08 | Height |
| rs12347744 | 9 | 97575273 | 1.00 | C | T | 0.06 | 0.01 | 0.08 | 0.05 | 0.05 | 0.10 | 0.03 | 0.11 | 0.11 | 0.07 | Height |
| rs4448343 | 9 | 98266370 | 1.00 | G | A | 0.04 | 0.00 | 0.01 | 0.03 | 0.04 | 0.05 | 0.02 | 0.05 | -0.01 | 0.03 | Height |
| rs1329393 | 9 | 98318926 | 0.89 | T | C | 0.04 | 0.00 | -0.02 | 0.03 | -0.05 | 0.07 | -0.10 | 0.07 | 0.01 | 0.04 | Height |
| rs817300 | 9 | 98380222 | 0.92 | G | A | 0.07 | 0.01 | 0.03 | 0.05 | 0.01 | 0.09 | 0.01 | 0.10 | 0.05 | 0.07 | Height |
| rs10990303 | 9 | 98410405 | 1.00 | T | C | 0.03 | 0.00 | -0.03 | 0.03 | 0.03 | 0.06 | -0.06 | 0.06 | -0.04 | 0.04 | Height |
| rs7870753 | 9 | 99201585 | 1.00 | G | A | 0.04 | 0.00 | -0.01 | 0.03 | 0.04 | 0.06 | -0.04 | 0.06 | -0.03 | 0.04 | Height |
| rs953199 | 9 | 100482976 | 1.00 | C | A | 0.02 | 0.00 | -0.05 | 0.03 | -0.03 | 0.06 | -0.08 | 0.06 | -0.05 | 0.04 | Height |
| rs989393 | 9 | 101743336 | 0.99 | T | C | 0.02 | 0.00 | 0.00 | 0.03 | -0.02 | 0.05 | 0.01 | 0.05 | 0.01 | 0.04 | Height |
| rs10820814 | 9 | 108304500 | 0.99 | C | A | 0.03 | 0.01 | -0.04 | 0.04 | 0.00 | 0.08 | 0.06 | 0.09 | -0.10 | 0.06 | Height |
| rs9409082 | 9 | 108901049 | 0.99 | C | T | 0.03 | 0.00 | -0.02 | 0.03 | -0.02 | 0.05 | 0.04 | 0.06 | -0.04 | 0.04 | Height |
| rs902143 | 9 | 109181911 | 1.00 | T | C | 0.02 | 0.00 | 0.01 | 0.02 | 0.00 | 0.05 | 0.04 | 0.05 | 0.00 | 0.03 | Height |
| rs2451948 | 9 | 109518208 | 1.00 | G | A | 0.02 | 0.00 | 0.11 | 0.03 | 0.06 | 0.06 | 0.04 | 0.07 | 0.16 | 0.04 | Height |
| rs7027110 | 9 | 109599046 | 1.00 | A | G | 0.03 | 0.00 | 0.02 | 0.03 | 0.10 | 0.06 | 0.02 | 0.06 | -0.02 | 0.04 | Height |
| rs3739707 | 9 | 113792706 | 1.00 | C | A | 0.02 | 0.00 | -0.03 | 0.03 | -0.04 | 0.06 | -0.04 | 0.06 | -0.03 | 0.04 | Height |
| rs999599 | 9 | 117011595 | 1.00 | T | C | 0.02 | 0.00 | -0.04 | 0.02 | 0.01 | 0.05 | -0.03 | 0.05 | -0.06 | 0.03 | Height |
| rs10759774 | 9 | 118169080 | 1.00 | A | G | 0.02 | 0.00 | -0.03 | 0.02 | -0.10 | 0.05 | 0.03 | 0.05 | -0.02 | 0.03 | Height |
| rs10119624 | 9 | 118305438 | 1.00 | A | G | 0.03 | 0.00 | -0.02 | 0.03 | -0.03 | 0.05 | 0.02 | 0.05 | -0.03 | 0.03 | Height |
| rs7033487 | 9 | 119129257 | 1.00 | T | C | 0.04 | 0.00 | -0.02 | 0.03 | -0.05 | 0.06 | 0.00 | 0.06 | -0.01 | 0.04 | Height |
| rs10817960 | 9 | 119688061 | 0.97 | A | G | 0.03 | 0.00 | -0.04 | 0.03 | -0.02 | 0.06 | 0.02 | 0.06 | -0.08 | 0.05 | Height |
| rs7466269 | 9 | 133464084 | 1.00 | A | G | 0.03 | 0.00 | 0.03 | 0.03 | -0.01 | 0.05 | 0.09 | 0.05 | 0.02 | 0.03 | Height |
| rs3132297 | 9 | 137301866 | 0.98 | G | A | 0.02 | 0.00 | 0.09 | 0.03 | 0.10 | 0.06 | 0.04 | 0.07 | 0.10 | 0.04 | Height |
| rs7849585 | 9 | 139111870 | 0.99 | T | G | 0.04 | 0.00 | -0.03 | 0.03 | -0.07 | 0.05 | -0.03 | 0.05 | -0.01 | 0.03 | Height |
| rs3812591 | 9 | 139341612 | 0.99 | C | T | 0.02 | 0.00 | 0.01 | 0.03 | 0.02 | 0.05 | -0.02 | 0.06 | 0.02 | 0.04 | Height |
| rs4332428 | 10 | 4965434 | 1.00 | A | G | 0.04 | 0.00 | -0.01 | 0.04 | 0.02 | 0.07 | 0.02 | 0.08 | -0.04 | 0.05 | Height |
| rs12779328 | 10 | 12943973 | 0.99 | C | T | 0.03 | 0.00 | -0.03 | 0.03 | 0.00 | 0.05 | -0.02 | 0.05 | -0.04 | 0.04 | Height |
| rs4350272 | 10 | 25056118 | 0.99 | A | G | 0.02 | 0.00 | 0.06 | 0.03 | 0.06 | 0.05 | 0.08 | 0.06 | 0.05 | 0.04 | Height |
| rs7069985 | 10 | 27890831 | 0.99 | G | A | 0.02 | 0.00 | -0.06 | 0.03 | 0.08 | 0.06 | -0.15 | 0.06 | -0.08 | 0.04 | Height |
| rs10995319 | 10 | 52762887 | 1.00 | T | C | 0.02 | 0.00 | 0.01 | 0.03 | 0.01 | 0.06 | 0.04 | 0.06 | 0.00 | 0.04 | Height |
| rs1171615 | 10 | 61469090 | 1.00 | C | T | 0.02 | 0.00 | -0.03 | 0.03 | -0.01 | 0.06 | 0.06 | 0.06 | -0.08 | 0.04 | Height |
| rs10997979 | 10 | 69937192 | 1.00 | G | A | 0.02 | 0.00 | 0.01 | 0.02 | 0.01 | 0.05 | -0.03 | 0.05 | 0.03 | 0.03 | Height |
| rs4746769 | 10 | 70196580 | 1.00 | T | C | 0.02 | 0.00 | -0.02 | 0.03 | 0.00 | 0.07 | -0.05 | 0.07 | -0.02 | 0.05 | Height |
| rs1815314 | 10 | 80928793 | 1.00 | G | A | 0.03 | 0.00 | -0.04 | 0.02 | -0.04 | 0.05 | 0.02 | 0.05 | -0.07 | 0.03 | Height |
| rs2631676 | 10 | 93037409 | 0.99 | G | A | 0.03 | 0.00 | -0.04 | 0.03 | -0.07 | 0.06 | -0.10 | 0.06 | 0.01 | 0.04 | Height |
| rs915506 | 10 | 97805074 | 1.00 | G | A | 0.02 | 0.00 | -0.03 | 0.02 | 0.02 | 0.05 | -0.05 | 0.05 | -0.04 | 0.03 | Height |
| rs11599750 | 10 | 101805442 | 1.00 | C | T | 0.02 | 0.00 | -0.02 | 0.02 | 0.01 | 0.05 | -0.02 | 0.05 | -0.03 | 0.03 | Height |
| rs10883563 | 10 | 102684380 | 1.00 | A | C | 0.02 | 0.00 | -0.01 | 0.02 | -0.03 | 0.05 | 0.02 | 0.05 | -0.02 | 0.03 | Height |
| rs7899004 | 10 | 104341435 | 1.00 | T | C | 0.02 | 0.00 | -0.04 | 0.02 | -0.02 | 0.05 | -0.07 | 0.05 | -0.03 | 0.03 | Height |
| rs6584575 | 10 | 105577409 | 1.00 | A | G | 0.03 | 0.01 | -0.04 | 0.04 | 0.06 | 0.08 | -0.10 | 0.08 | -0.06 | 0.05 | Height |
| rs291979 | 10 | 121129797 | 1.00 | A | G | 0.03 | 0.00 | -0.05 | 0.03 | -0.05 | 0.06 | -0.02 | 0.06 | -0.07 | 0.04 | Height |
| rs1614303 | 10 | 123396806 | 1.00 | T | G | 0.02 | 0.00 | -0.02 | 0.03 | 0.01 | 0.06 | 0.00 | 0.06 | -0.05 | 0.04 | Height |
| rs7097701 | 10 | 124171857 | 1.00 | C | T | 0.02 | 0.00 | 0.01 | 0.02 | -0.04 | 0.05 | -0.01 | 0.05 | 0.03 | 0.03 | Height |
| rs10794175 | 10 | 126358073 | 1.00 | T | G | 0.02 | 0.00 | 0.04 | 0.02 | 0.06 | 0.05 | 0.06 | 0.05 | 0.03 | 0.03 | Height |
| rs11245515 | 10 | 126824068 | 1.00 | T | C | 0.02 | 0.00 | -0.02 | 0.02 | -0.06 | 0.05 | -0.03 | 0.05 | 0.01 | 0.03 | Height |
| rs11244750 | 10 | 127673877 | 0.99 | T | C | 0.02 | 0.00 | 0.01 | 0.03 | -0.04 | 0.05 | 0.01 | 0.05 | 0.03 | 0.04 | Height |
| rs2272566 | 11 | 244552 | 1.00 | A | G | 0.02 | 0.00 | -0.02 | 0.02 | -0.02 | 0.05 | -0.06 | 0.05 | -0.01 | 0.03 | Height |
| rs2735469 | 11 | 2022804 | 1.00 | A | G | 0.03 | 0.00 | -0.02 | 0.03 | 0.12 | 0.07 | -0.04 | 0.07 | -0.08 | 0.05 | Height |
| rs4320932 | 11 | 2171601 | 1.00 | T | C | 0.03 | 0.00 | 0.00 | 0.03 | -0.02 | 0.06 | 0.00 | 0.06 | 0.02 | 0.04 | Height |
| rs17659078 | 11 | 2284590 | 1.00 | A | C | 0.02 | 0.00 | 0.03 | 0.03 | -0.10 | 0.05 | 0.08 | 0.06 | 0.07 | 0.04 | Height |
| rs2237886 | 11 | 2810731 | 1.00 | T | C | 0.04 | 0.00 | 0.00 | 0.04 | -0.04 | 0.08 | 0.05 | 0.08 | 0.00 | 0.05 | Height |
| rs6485978 | 11 | 12678415 | 1.00 | C | T | 0.02 | 0.00 | -0.01 | 0.02 | -0.03 | 0.05 | -0.02 | 0.05 | 0.00 | 0.03 | Height |
| rs2099745 | 11 | 12924265 | 1.00 | G | A | 0.04 | 0.01 | -0.09 | 0.05 | -0.09 | 0.09 | -0.15 | 0.11 | -0.06 | 0.07 | Height |
| rs10766065 | 11 | 13277961 | 1.00 | C | T | 0.02 | 0.00 | 0.04 | 0.03 | 0.04 | 0.05 | 0.10 | 0.06 | 0.01 | 0.04 | Height |
| rs2915404 | 11 | 14404825 | 1.00 | T | C | 0.02 | 0.00 | -0.03 | 0.02 | -0.01 | 0.05 | -0.02 | 0.05 | -0.05 | 0.03 | Height |
| rs10767838 | 11 | 30347927 | 1.00 | A | G | 0.03 | 0.00 | 0.01 | 0.03 | -0.03 | 0.05 | -0.01 | 0.06 | 0.04 | 0.04 | Height |
| rs3802758 | 11 | 45936035 | 0.97 | A | G | 0.04 | 0.01 | 0.05 | 0.04 | -0.04 | 0.09 | 0.12 | 0.09 | 0.07 | 0.06 | Height |
| rs1681630 | 11 | 47969152 | 1.00 | T | C | 0.03 | 0.00 | -0.04 | 0.02 | 0.02 | 0.05 | -0.01 | 0.05 | -0.08 | 0.03 | Height |
| rs1945237 | 11 | 56230069 | 1.00 | C | T | 0.03 | 0.01 | 0.04 | 0.04 | 0.03 | 0.09 | -0.03 | 0.09 | 0.07 | 0.06 | Height |
| rs3782089 | 11 | 65336819 | 0.99 | C | T | 0.05 | 0.01 | 0.06 | 0.05 | -0.08 | 0.10 | 0.12 | 0.10 | 0.10 | 0.06 | Height |
| rs7112925 | 11 | 66826160 | 1.00 | C | T | 0.02 | 0.00 | -0.03 | 0.02 | -0.08 | 0.05 | 0.00 | 0.05 | -0.02 | 0.03 | Height |
| rs3750972 | 11 | 68830628 | 0.93 | G | T | 0.02 | 0.00 | 0.01 | 0.02 | 0.02 | 0.05 | 0.02 | 0.05 | 0.00 | 0.03 | Height |
| rs4357716 | 11 | 69163161 | 0.99 | T | C | 0.03 | 0.00 | 0.00 | 0.03 | 0.04 | 0.07 | 0.06 | 0.07 | -0.05 | 0.05 | Height |
| rs2509133 | 11 | 69933717 | 0.99 | C | T | 0.02 | 0.00 | 0.00 | 0.02 | -0.02 | 0.05 | -0.05 | 0.05 | 0.03 | 0.03 | Height |
| rs11236294 | 11 | 74739934 | 1.00 | T | G | 0.02 | 0.00 | -0.03 | 0.03 | -0.02 | 0.05 | 0.02 | 0.05 | -0.05 | 0.04 | Height |
| rs606452 | 11 | 75276178 | 1.00 | A | C | 0.04 | 0.00 | -0.01 | 0.03 | -0.11 | 0.07 | 0.03 | 0.07 | 0.01 | 0.05 | Height |
| rs10790381 | 11 | 120257495 | 1.00 | A | G | 0.03 | 0.00 | 0.07 | 0.03 | 0.04 | 0.06 | 0.08 | 0.07 | 0.09 | 0.04 | Height |
| rs1461503 | 11 | 122845075 | 1.00 | C | A | 0.02 | 0.00 | 0.05 | 0.02 | 0.09 | 0.05 | 0.01 | 0.05 | 0.05 | 0.03 | Height |
| rs11612228 | 12 | 576984 | 0.98 | T | C | 0.02 | 0.00 | -0.03 | 0.03 | -0.09 | 0.05 | 0.05 | 0.05 | -0.04 | 0.03 | Height |
| rs7299326 | 12 | 1573005 | 1.00 | C | T | 0.04 | 0.01 | 0.06 | 0.05 | 0.14 | 0.11 | 0.00 | 0.10 | 0.06 | 0.07 | Height |
| rs2856321 | 12 | 11855773 | 1.00 | G | A | 0.03 | 0.00 | -0.01 | 0.02 | 0.01 | 0.05 | -0.01 | 0.05 | -0.02 | 0.03 | Height |
| rs12228415 | 12 | 14520701 | 1.00 | G | A | 0.02 | 0.00 | -0.05 | 0.02 | -0.06 | 0.05 | -0.08 | 0.05 | -0.02 | 0.03 | Height |
| rs4326884 | 12 | 20536371 | 1.00 | A | G | 0.02 | 0.00 | 0.01 | 0.02 | -0.08 | 0.05 | -0.01 | 0.05 | 0.06 | 0.03 | Height |
| rs10770705 | 12 | 20857467 | 1.00 | A | C | 0.03 | 0.00 | 0.03 | 0.03 | 0.02 | 0.05 | 0.02 | 0.05 | 0.04 | 0.04 | Height |
| rs10492364 | 12 | 28112256 | 0.99 | T | C | 0.02 | 0.00 | 0.00 | 0.03 | 0.04 | 0.05 | 0.02 | 0.06 | -0.02 | 0.04 | Height |
| rs11049611 | 12 | 28600244 | 1.00 | C | T | 0.04 | 0.00 | 0.03 | 0.03 | -0.01 | 0.05 | 0.04 | 0.05 | 0.04 | 0.04 | Height |
| rs10843390 | 12 | 29496991 | 1.00 | T | C | 0.03 | 0.00 | -0.01 | 0.03 | -0.09 | 0.05 | 0.11 | 0.05 | -0.03 | 0.04 | Height |
| rs10880969 | 12 | 46827023 | 1.00 | C | T | 0.02 | 0.00 | 0.01 | 0.03 | 0.00 | 0.05 | 0.02 | 0.05 | 0.02 | 0.04 | Height |
| rs2306694 | 12 | 56680636 | 1.00 | G | A | 0.05 | 0.01 | -0.08 | 0.05 | 0.01 | 0.10 | -0.07 | 0.10 | -0.12 | 0.07 | Height |
| rs10877030 | 12 | 58256714 | 1.00 | T | G | 0.02 | 0.00 | 0.01 | 0.03 | 0.00 | 0.05 | 0.03 | 0.05 | 0.01 | 0.04 | Height |
| rs17122659 | 12 | 59956923 | 0.96 | G | A | 0.03 | 0.00 | -0.02 | 0.04 | -0.08 | 0.07 | 0.02 | 0.08 | -0.01 | 0.05 | Height |
| rs2164968 | 12 | 65677086 | 1.00 | C | T | 0.02 | 0.00 | 0.01 | 0.02 | 0.02 | 0.05 | 0.01 | 0.05 | 0.01 | 0.03 | Height |
| rs8756 | 12 | 66359752 | 1.00 | C | A | 0.05 | 0.00 | 0.01 | 0.02 | 0.03 | 0.05 | 0.06 | 0.05 | -0.02 | 0.03 | Height |
| rs10748128 | 12 | 69827658 | 1.00 | T | G | 0.04 | 0.00 | 0.01 | 0.02 | 0.03 | 0.05 | 0.04 | 0.05 | -0.01 | 0.03 | Height |
| rs17783015 | 12 | 90231386 | 0.99 | C | T | 0.02 | 0.00 | 0.04 | 0.03 | 0.03 | 0.07 | 0.11 | 0.07 | 0.01 | 0.05 | Height |
| rs11107062 | 12 | 93919840 | 0.99 | T | C | 0.03 | 0.00 | -0.02 | 0.03 | -0.04 | 0.06 | -0.04 | 0.06 | 0.00 | 0.04 | Height |
| rs3825199 | 12 | 93976954 | 1.00 | G | A | 0.05 | 0.00 | 0.03 | 0.03 | 0.05 | 0.06 | -0.03 | 0.06 | 0.04 | 0.04 | Height |
| rs10859567 | 12 | 94126925 | 0.99 | T | G | 0.04 | 0.00 | -0.02 | 0.02 | 0.04 | 0.05 | 0.00 | 0.05 | -0.05 | 0.03 | Height |
| rs833706 | 12 | 103062597 | 0.99 | A | G | 0.02 | 0.00 | 0.02 | 0.03 | -0.01 | 0.06 | 0.04 | 0.06 | 0.02 | 0.04 | Height |
| rs2164747 | 12 | 104344836 | 1.00 | G | A | 0.03 | 0.00 | 0.02 | 0.04 | 0.08 | 0.08 | -0.02 | 0.08 | 0.01 | 0.05 | Height |
| rs2888893 | 12 | 107338631 | 1.00 | C | T | 0.02 | 0.00 | -0.02 | 0.02 | 0.01 | 0.05 | 0.00 | 0.05 | -0.05 | 0.03 | Height |
| rs11616067 | 12 | 116393174 | 0.98 | A | G | 0.02 | 0.00 | 0.04 | 0.03 | 0.01 | 0.06 | 0.08 | 0.06 | 0.04 | 0.04 | Height |
| rs11835818 | 12 | 122494809 | 1.00 | C | T | 0.02 | 0.00 | -0.02 | 0.02 | -0.06 | 0.05 | 0.01 | 0.05 | -0.01 | 0.03 | Height |
| rs7980687 | 12 | 123822711 | 0.99 | A | G | 0.04 | 0.00 | -0.02 | 0.03 | -0.04 | 0.06 | 0.01 | 0.06 | -0.03 | 0.04 | Height |
| rs1809889 | 12 | 124801226 | 1.00 | T | C | 0.03 | 0.00 | -0.02 | 0.03 | -0.10 | 0.05 | -0.02 | 0.06 | 0.01 | 0.04 | Height |
| rs1199734 | 13 | 21570246 | 0.99 | G | T | 0.02 | 0.00 | 0.05 | 0.03 | 0.06 | 0.06 | 0.02 | 0.07 | 0.06 | 0.04 | Height |
| rs11618507 | 13 | 30172751 | 0.96 | T | G | 0.02 | 0.00 | -0.01 | 0.03 | -0.05 | 0.06 | -0.02 | 0.06 | 0.01 | 0.04 | Height |
| rs12323101 | 13 | 33143406 | 1.00 | A | G | 0.02 | 0.00 | 0.02 | 0.02 | 0.07 | 0.05 | -0.08 | 0.05 | 0.03 | 0.03 | Height |
| rs12863103 | 13 | 33723244 | 1.00 | C | T | 0.02 | 0.00 | 0.01 | 0.03 | -0.01 | 0.05 | 0.06 | 0.06 | 0.01 | 0.04 | Height |
| rs6561319 | 13 | 47112120 | 1.00 | A | C | 0.02 | 0.00 | 0.00 | 0.02 | 0.02 | 0.05 | 0.05 | 0.05 | -0.03 | 0.03 | Height |
| rs12871822 | 13 | 49201040 | 1.00 | G | T | 0.02 | 0.00 | 0.00 | 0.03 | -0.01 | 0.05 | 0.03 | 0.05 | -0.01 | 0.03 | Height |
| rs7334755 | 13 | 50469913 | 0.98 | C | T | 0.02 | 0.00 | 0.03 | 0.03 | 0.01 | 0.06 | -0.04 | 0.06 | 0.06 | 0.04 | Height |
| rs2687950 | 13 | 50718468 | 1.00 | T | C | 0.03 | 0.00 | 0.01 | 0.03 | 0.03 | 0.06 | 0.03 | 0.06 | -0.01 | 0.04 | Height |
| rs1753637 | 13 | 51084173 | 1.00 | T | G | 0.02 | 0.00 | -0.03 | 0.03 | -0.01 | 0.05 | -0.03 | 0.05 | -0.04 | 0.04 | Height |
| rs3118905 | 13 | 51105334 | 1.00 | G | A | 0.04 | 0.00 | -0.03 | 0.03 | -0.05 | 0.05 | -0.03 | 0.06 | -0.02 | 0.04 | Height |
| rs3818416 | 13 | 78474468 | 1.00 | C | A | 0.02 | 0.00 | -0.01 | 0.03 | 0.00 | 0.06 | -0.08 | 0.06 | 0.02 | 0.04 | Height |
| rs11616380 | 13 | 80705315 | 0.99 | T | G | 0.02 | 0.00 | 0.01 | 0.03 | 0.00 | 0.05 | 0.07 | 0.06 | -0.01 | 0.04 | Height |
| rs6563199 | 13 | 81550449 | 0.99 | T | C | 0.02 | 0.00 | 0.01 | 0.02 | 0.05 | 0.05 | 0.01 | 0.05 | 0.00 | 0.03 | Height |
| rs7319045 | 13 | 92024574 | 0.99 | A | G | 0.02 | 0.00 | 0.05 | 0.02 | 0.09 | 0.05 | 0.07 | 0.05 | 0.01 | 0.03 | Height |
| rs8017130 | 14 | 23759156 | 1.00 | G | A | 0.02 | 0.00 | 0.01 | 0.03 | 0.01 | 0.05 | -0.02 | 0.05 | 0.01 | 0.04 | Height |
| rs1950500 | 14 | 24830850 | 1.00 | T | C | 0.03 | 0.00 | 0.02 | 0.03 | -0.04 | 0.05 | -0.02 | 0.05 | 0.07 | 0.04 | Height |
| rs12435366 | 14 | 35838389 | 1.00 | C | T | 0.02 | 0.00 | 0.07 | 0.03 | 0.09 | 0.06 | 0.11 | 0.06 | 0.05 | 0.04 | Height |
| rs10131337 | 14 | 37144516 | 0.98 | T | C | 0.03 | 0.00 | 0.02 | 0.03 | 0.12 | 0.06 | 0.02 | 0.06 | -0.03 | 0.04 | Height |
| rs8006657 | 14 | 55245149 | 0.97 | G | A | 0.02 | 0.00 | 0.00 | 0.02 | 0.08 | 0.05 | 0.00 | 0.05 | -0.03 | 0.03 | Height |
| rs11624136 | 14 | 59688820 | 1.00 | A | G | 0.02 | 0.00 | -0.03 | 0.02 | -0.06 | 0.05 | 0.00 | 0.05 | -0.04 | 0.03 | Height |
| rs2093210 | 14 | 60957279 | 0.99 | C | T | 0.04 | 0.00 | 0.02 | 0.02 | 0.01 | 0.05 | 0.06 | 0.05 | 0.00 | 0.03 | Height |
| rs2781373 | 14 | 65568215 | 1.00 | G | A | 0.02 | 0.00 | 0.00 | 0.02 | -0.04 | 0.05 | 0.11 | 0.05 | -0.02 | 0.03 | Height |
| rs1980850 | 14 | 68647188 | 1.00 | G | A | 0.03 | 0.00 | 0.03 | 0.03 | -0.03 | 0.06 | 0.07 | 0.06 | 0.03 | 0.04 | Height |
| rs2058092 | 14 | 73932966 | 1.00 | T | C | 0.02 | 0.00 | -0.03 | 0.02 | -0.04 | 0.05 | -0.05 | 0.05 | -0.01 | 0.03 | Height |
| rs862034 | 14 | 74990746 | 1.00 | G | A | 0.03 | 0.00 | 0.01 | 0.02 | 0.02 | 0.05 | -0.05 | 0.05 | 0.03 | 0.03 | Height |
| rs10140101 | 14 | 75038689 | 1.00 | T | C | 0.02 | 0.00 | 0.04 | 0.02 | 0.02 | 0.05 | 0.05 | 0.05 | 0.04 | 0.03 | Height |
| rs7154721 | 14 | 92427348 | 1.00 | T | C | 0.03 | 0.00 | -0.01 | 0.02 | -0.02 | 0.05 | -0.06 | 0.05 | 0.02 | 0.03 | Height |
| rs1036477 | 15 | 48914926 | 1.00 | A | G | 0.03 | 0.00 | 0.02 | 0.04 | 0.05 | 0.08 | -0.12 | 0.08 | 0.07 | 0.05 | Height |
| rs10744956 | 15 | 51269629 | 1.00 | G | A | 0.02 | 0.00 | -0.01 | 0.03 | -0.01 | 0.06 | 0.01 | 0.06 | -0.01 | 0.04 | Height |
| rs16964211 | 15 | 51530495 | 1.00 | G | A | 0.04 | 0.01 | 0.02 | 0.05 | 0.05 | 0.10 | 0.05 | 0.12 | -0.01 | 0.07 | Height |
| rs782930 | 15 | 61408362 | 1.00 | G | A | 0.02 | 0.00 | 0.01 | 0.03 | 0.11 | 0.05 | -0.04 | 0.05 | -0.02 | 0.03 | Height |
| rs7177711 | 15 | 62379971 | 1.00 | A | G | 0.02 | 0.00 | -0.03 | 0.02 | 0.02 | 0.05 | 0.04 | 0.05 | -0.08 | 0.03 | Height |
| rs7162825 | 15 | 63439186 | 0.99 | T | C | 0.02 | 0.00 | 0.05 | 0.02 | 0.02 | 0.05 | 0.05 | 0.05 | 0.07 | 0.03 | Height |
| rs17264185 | 15 | 66997087 | 1.00 | G | A | 0.02 | 0.00 | -0.01 | 0.03 | -0.03 | 0.05 | 0.03 | 0.06 | -0.01 | 0.04 | Height |
| rs731874 | 15 | 67446831 | 1.00 | A | G | 0.02 | 0.00 | 0.03 | 0.03 | 0.00 | 0.05 | 0.03 | 0.06 | 0.04 | 0.04 | Height |
| rs10152591 | 15 | 70048157 | 1.00 | A | C | 0.04 | 0.01 | -0.03 | 0.04 | -0.08 | 0.08 | -0.08 | 0.09 | 0.01 | 0.06 | Height |
| rs975210 | 15 | 70364352 | 0.98 | A | G | 0.03 | 0.00 | 0.04 | 0.03 | 0.08 | 0.06 | -0.06 | 0.07 | 0.05 | 0.04 | Height |
| rs11634405 | 15 | 72084693 | 1.00 | A | G | 0.02 | 0.00 | 0.07 | 0.02 | 0.07 | 0.05 | 0.12 | 0.05 | 0.04 | 0.03 | Height |
| rs12904334 | 15 | 72842705 | 0.99 | A | G | 0.09 | 0.01 | 0.10 | 0.10 | -0.02 | 0.19 | 0.17 | 0.22 | 0.14 | 0.14 | Height |
| rs5742915 | 15 | 74336633 | 1.00 | C | T | 0.04 | 0.00 | -0.04 | 0.02 | 0.01 | 0.05 | -0.11 | 0.05 | -0.04 | 0.03 | Height |
| rs12914466 | 15 | 81836638 | 1.00 | A | G | 0.02 | 0.00 | 0.03 | 0.02 | 0.07 | 0.05 | 0.05 | 0.05 | -0.01 | 0.03 | Height |
| rs2257011 | 15 | 84266145 | 0.98 | T | G | 0.03 | 0.00 | -0.03 | 0.02 | 0.04 | 0.05 | -0.03 | 0.05 | -0.05 | 0.03 | Height |
| rs11855014 | 15 | 85728834 | 1.00 | G | A | 0.02 | 0.00 | 0.05 | 0.03 | 0.11 | 0.05 | 0.04 | 0.05 | 0.03 | 0.04 | Height |
| rs11633371 | 15 | 89356832 | 1.00 | T | G | 0.02 | 0.00 | -0.02 | 0.02 | -0.04 | 0.05 | -0.09 | 0.05 | 0.02 | 0.03 | Height |
| rs16942341 | 15 | 89388905 | 0.93 | C | T | 0.11 | 0.01 | 0.03 | 0.07 | -0.03 | 0.14 | 0.00 | 0.15 | 0.07 | 0.10 | Height |
| rs2280470 | 15 | 89395626 | 0.99 | A | G | 0.03 | 0.00 | 0.00 | 0.03 | 0.03 | 0.05 | -0.04 | 0.05 | 0.01 | 0.03 | Height |
| rs2238300 | 15 | 89851580 | 1.00 | G | A | 0.02 | 0.00 | 0.06 | 0.02 | 0.15 | 0.05 | 0.04 | 0.05 | 0.03 | 0.03 | Height |
| rs8028843 | 15 | 94028149 | 0.99 | T | C | 0.02 | 0.00 | -0.05 | 0.02 | -0.04 | 0.05 | -0.03 | 0.05 | -0.07 | 0.03 | Height |
| rs7181724 | 15 | 94551607 | 1.00 | G | A | 0.02 | 0.00 | 0.02 | 0.02 | 0.01 | 0.05 | 0.03 | 0.05 | 0.01 | 0.03 | Height |
| rs2573625 | 15 | 100513158 | 0.97 | T | C | 0.03 | 0.00 | -0.04 | 0.03 | -0.07 | 0.05 | -0.02 | 0.05 | -0.04 | 0.03 | Height |
| rs4246302 | 15 | 100687967 | 0.99 | G | A | 0.03 | 0.00 | 0.01 | 0.03 | 0.10 | 0.05 | -0.02 | 0.05 | -0.01 | 0.04 | Height |
| rs4548838 | 15 | 100761190 | 0.99 | T | C | 0.03 | 0.00 | 0.02 | 0.02 | 0.03 | 0.05 | -0.04 | 0.05 | 0.04 | 0.03 | Height |
| rs7170986 | 15 | 101632867 | 0.96 | G | A | 0.02 | 0.00 | -0.01 | 0.03 | -0.04 | 0.06 | 0.09 | 0.06 | -0.04 | 0.04 | Height |
| rs8042424 | 15 | 101762539 | 0.99 | C | T | 0.02 | 0.00 | -0.03 | 0.03 | -0.06 | 0.06 | 0.00 | 0.06 | -0.02 | 0.04 | Height |
| rs11648796 | 16 | 792190 | 0.92 | G | A | 0.03 | 0.00 | -0.07 | 0.03 | -0.04 | 0.06 | -0.04 | 0.06 | -0.10 | 0.04 | Height |
| rs12597498 | 16 | 990815 | 1.00 | T | C | 0.02 | 0.00 | 0.00 | 0.02 | 0.01 | 0.05 | -0.04 | 0.05 | 0.02 | 0.03 | Height |
| rs2531992 | 16 | 4021734 | 0.99 | G | A | 0.02 | 0.00 | 0.00 | 0.03 | -0.06 | 0.07 | 0.02 | 0.07 | 0.02 | 0.05 | Height |
| rs960006 | 16 | 4911195 | 0.99 | C | T | 0.02 | 0.00 | -0.01 | 0.02 | 0.00 | 0.05 | -0.01 | 0.05 | -0.01 | 0.03 | Height |
| rs1659127 | 16 | 14388305 | 0.97 | A | G | 0.03 | 0.00 | -0.03 | 0.03 | 0.05 | 0.05 | -0.15 | 0.05 | -0.01 | 0.03 | Height |
| rs2023693 | 16 | 20880040 | 1.00 | G | A | 0.02 | 0.00 | 0.04 | 0.02 | -0.03 | 0.05 | 0.07 | 0.05 | 0.05 | 0.03 | Height |
| rs11642612 | 16 | 30030195 | 1.00 | C | A | 0.02 | 0.00 | 0.01 | 0.02 | 0.11 | 0.05 | -0.03 | 0.05 | -0.01 | 0.03 | Height |
| rs4785393 | 16 | 50259483 | 0.99 | G | A | 0.02 | 0.00 | -0.01 | 0.03 | 0.01 | 0.06 | -0.05 | 0.06 | -0.01 | 0.04 | Height |
| rs9929889 | 16 | 51094038 | 0.98 | C | T | 0.02 | 0.00 | -0.04 | 0.02 | 0.00 | 0.05 | -0.07 | 0.05 | -0.05 | 0.03 | Height |
| rs8058684 | 16 | 53515118 | 0.99 | A | G | 0.02 | 0.00 | 0.04 | 0.03 | -0.05 | 0.05 | 0.03 | 0.05 | 0.08 | 0.04 | Height |
| rs217181 | 16 | 72114002 | 0.98 | T | C | 0.02 | 0.00 | -0.01 | 0.03 | -0.11 | 0.06 | -0.02 | 0.06 | 0.03 | 0.04 | Height |
| rs11640018 | 16 | 75328308 | 0.99 | C | T | 0.02 | 0.00 | -0.09 | 0.02 | -0.05 | 0.05 | -0.07 | 0.05 | -0.11 | 0.03 | Height |
| rs4243206 | 16 | 81589983 | 1.00 | A | C | 0.02 | 0.00 | 0.04 | 0.03 | 0.01 | 0.06 | 0.07 | 0.06 | 0.04 | 0.04 | Height |
| rs2326458 | 16 | 84987679 | 1.00 | C | A | 0.02 | 0.00 | -0.01 | 0.03 | 0.02 | 0.06 | -0.05 | 0.06 | -0.01 | 0.04 | Height |
| rs4843367 | 16 | 86417890 | 0.99 | C | T | 0.02 | 0.00 | -0.04 | 0.03 | -0.10 | 0.05 | 0.05 | 0.05 | -0.05 | 0.03 | Height |
| rs300039 | 16 | 86688976 | 1.00 | C | T | 0.02 | 0.00 | 0.02 | 0.03 | 0.06 | 0.06 | 0.00 | 0.06 | 0.01 | 0.04 | Height |
| rs8052560 | 16 | 88777242 | 0.91 | A | C | 0.04 | 0.00 | 0.02 | 0.03 | 0.05 | 0.06 | 0.06 | 0.07 | -0.01 | 0.04 | Height |
| rs2377058 | 16 | 89734831 | 1.00 | G | A | 0.03 | 0.00 | -0.01 | 0.02 | 0.07 | 0.05 | -0.12 | 0.05 | -0.01 | 0.03 | Height |
| rs11861084 | 16 | 89875710 | 1.00 | A | C | 0.02 | 0.00 | 0.02 | 0.02 | -0.04 | 0.05 | 0.05 | 0.05 | 0.04 | 0.03 | Height |
| rs870183 | 17 | 599811 | 1.00 | G | A | 0.02 | 0.00 | 0.00 | 0.02 | -0.03 | 0.05 | 0.06 | 0.05 | -0.01 | 0.03 | Height |
| rs9217 | 17 | 7363088 | 1.00 | C | T | 0.03 | 0.00 | -0.04 | 0.03 | -0.06 | 0.05 | -0.06 | 0.05 | -0.03 | 0.03 | Height |
| rs8073177 | 17 | 7440584 | 1.00 | C | T | 0.02 | 0.00 | 0.04 | 0.03 | 0.05 | 0.06 | 0.03 | 0.06 | 0.03 | 0.04 | Height |
| rs1625895 | 17 | 7578115 | 1.00 | C | T | 0.03 | 0.00 | -0.10 | 0.03 | -0.09 | 0.07 | -0.17 | 0.07 | -0.07 | 0.05 | Height |
| rs4640244 | 17 | 21284223 | 1.00 | A | G | 0.03 | 0.00 | 0.03 | 0.02 | 0.04 | 0.05 | -0.05 | 0.05 | 0.05 | 0.03 | Height |
| rs3809790 | 17 | 27955540 | 1.00 | C | T | 0.02 | 0.00 | -0.01 | 0.02 | -0.01 | 0.05 | 0.03 | 0.05 | -0.02 | 0.03 | Height |
| rs9889755 | 17 | 29234505 | 1.00 | T | C | 0.04 | 0.01 | 0.01 | 0.04 | -0.10 | 0.08 | 0.15 | 0.08 | 0.00 | 0.05 | Height |
| rs3760318 | 17 | 29247715 | 1.00 | G | A | 0.05 | 0.00 | 0.03 | 0.02 | 0.04 | 0.05 | 0.00 | 0.05 | 0.04 | 0.03 | Height |
| rs2028067 | 17 | 30239698 | 0.99 | C | T | 0.03 | 0.00 | 0.04 | 0.03 | 0.09 | 0.06 | 0.09 | 0.07 | -0.01 | 0.04 | Height |
| rs2338115 | 17 | 36929578 | 1.00 | T | C | 0.02 | 0.00 | -0.03 | 0.02 | -0.03 | 0.05 | -0.01 | 0.05 | -0.05 | 0.03 | Height |
| rs584828 | 17 | 38599230 | 0.97 | C | T | 0.03 | 0.00 | 0.00 | 0.02 | 0.08 | 0.05 | -0.05 | 0.05 | -0.02 | 0.03 | Height |
| rs9766 | 17 | 40852841 | 0.99 | A | G | 0.02 | 0.00 | -0.02 | 0.02 | -0.02 | 0.05 | 0.02 | 0.05 | -0.03 | 0.03 | Height |
| rs4986172 | 17 | 43216281 | 1.00 | C | T | 0.04 | 0.00 | 0.02 | 0.02 | 0.02 | 0.05 | 0.10 | 0.05 | -0.01 | 0.03 | Height |
| rs8073371 | 17 | 46096276 | 1.00 | C | T | 0.03 | 0.00 | -0.04 | 0.03 | -0.03 | 0.06 | -0.03 | 0.06 | -0.05 | 0.04 | Height |
| rs318095 | 17 | 46974734 | 1.00 | T | C | 0.02 | 0.00 | 0.03 | 0.02 | -0.01 | 0.05 | 0.14 | 0.05 | 0.00 | 0.03 | Height |
| rs1401795 | 17 | 54839652 | 0.95 | A | G | 0.03 | 0.00 | 0.05 | 0.02 | 0.14 | 0.05 | 0.12 | 0.05 | -0.02 | 0.03 | Height |
| rs2079795 | 17 | 59496649 | 0.99 | T | C | 0.05 | 0.00 | -0.05 | 0.03 | -0.06 | 0.05 | -0.08 | 0.05 | -0.04 | 0.03 | Height |
| rs2378870 | 17 | 59638623 | 1.00 | T | C | 0.02 | 0.00 | -0.01 | 0.02 | -0.08 | 0.05 | 0.04 | 0.05 | -0.01 | 0.03 | Height |
| rs2044124 | 17 | 61845425 | 0.99 | T | C | 0.05 | 0.01 | -0.05 | 0.05 | 0.01 | 0.10 | -0.07 | 0.11 | -0.08 | 0.07 | Height |
| rs2070776 | 17 | 62007498 | 1.00 | G | A | 0.03 | 0.00 | -0.03 | 0.02 | -0.03 | 0.05 | 0.06 | 0.05 | -0.06 | 0.03 | Height |
| rs3923086 | 17 | 63549488 | 1.00 | C | A | 0.03 | 0.00 | 0.00 | 0.02 | 0.02 | 0.05 | -0.01 | 0.05 | 0.00 | 0.03 | Height |
| rs2072268 | 17 | 66303352 | 1.00 | G | A | 0.02 | 0.00 | -0.03 | 0.02 | -0.05 | 0.05 | -0.08 | 0.05 | 0.00 | 0.03 | Height |
| rs11867479 | 17 | 68090207 | 1.00 | T | C | 0.03 | 0.00 | 0.04 | 0.02 | 0.04 | 0.05 | 0.04 | 0.05 | 0.03 | 0.03 | Height |
| rs10083886 | 17 | 69923355 | 1.00 | T | C | 0.02 | 0.00 | 0.02 | 0.03 | 0.03 | 0.05 | 0.06 | 0.06 | -0.01 | 0.04 | Height |
| rs2117563 | 17 | 73368985 | 1.00 | G | A | 0.02 | 0.00 | 0.06 | 0.03 | 0.03 | 0.06 | 0.11 | 0.06 | 0.06 | 0.04 | Height |
| rs1552173 | 17 | 76718842 | 0.98 | C | T | 0.02 | 0.00 | -0.01 | 0.02 | -0.09 | 0.05 | 0.00 | 0.05 | 0.02 | 0.03 | Height |
| rs1478610 | 17 | 79422252 | 0.72 | A | G | 0.02 | 0.00 | 0.04 | 0.03 | 0.04 | 0.06 | 0.05 | 0.06 | 0.04 | 0.04 | Height |
| rs4239020 | 17 | 80176641 | 1.00 | C | T | 0.02 | 0.00 | 0.04 | 0.03 | 0.05 | 0.05 | 0.04 | 0.05 | 0.04 | 0.04 | Height |
| rs888403 | 18 | 2766938 | 0.97 | G | A | 0.02 | 0.00 | -0.03 | 0.03 | -0.06 | 0.05 | 0.00 | 0.05 | -0.02 | 0.04 | Height |
| rs692964 | 18 | 13094132 | 1.00 | G | A | 0.02 | 0.00 | 0.02 | 0.02 | -0.02 | 0.05 | 0.02 | 0.05 | 0.04 | 0.03 | Height |
| rs14062 | 18 | 19450303 | 1.00 | G | A | 0.02 | 0.00 | 0.03 | 0.03 | 0.00 | 0.05 | 0.04 | 0.05 | 0.05 | 0.04 | Height |
| rs4369779 | 18 | 20735408 | 1.00 | C | T | 0.06 | 0.00 | 0.00 | 0.03 | 0.07 | 0.06 | 0.05 | 0.06 | -0.06 | 0.04 | Height |
| rs11661645 | 18 | 45888770 | 0.99 | A | G | 0.02 | 0.00 | -0.06 | 0.03 | -0.09 | 0.05 | -0.06 | 0.05 | -0.04 | 0.04 | Height |
| rs12454567 | 18 | 46270114 | 1.00 | A | G | 0.03 | 0.00 | 0.01 | 0.04 | -0.08 | 0.08 | 0.07 | 0.08 | 0.03 | 0.05 | Height |
| rs2337143 | 18 | 46482070 | 1.00 | A | G | 0.02 | 0.00 | -0.03 | 0.03 | 0.00 | 0.05 | -0.05 | 0.05 | -0.03 | 0.03 | Height |
| rs12458127 | 18 | 46657358 | 1.00 | C | T | 0.04 | 0.01 | -0.05 | 0.05 | 0.13 | 0.10 | -0.11 | 0.11 | -0.11 | 0.07 | Height |
| rs11152213 | 18 | 57852948 | 1.00 | C | A | 0.03 | 0.00 | 0.04 | 0.03 | 0.06 | 0.06 | 0.00 | 0.06 | 0.04 | 0.04 | Height |
| rs8097893 | 18 | 74983055 | 1.00 | A | G | 0.04 | 0.01 | -0.03 | 0.06 | -0.09 | 0.11 | 0.10 | 0.12 | -0.06 | 0.09 | Height |
| rs11659752 | 18 | 77222862 | 0.99 | T | G | 0.02 | 0.00 | 0.06 | 0.03 | 0.03 | 0.05 | 0.10 | 0.05 | 0.07 | 0.04 | Height |
| rs11880992 | 19 | 2176403 | 1.00 | A | G | 0.03 | 0.00 | 0.02 | 0.02 | 0.00 | 0.05 | 0.01 | 0.05 | 0.04 | 0.03 | Height |
| rs2074977 | 19 | 3434028 | 0.99 | C | A | 0.03 | 0.00 | 0.00 | 0.03 | 0.01 | 0.05 | 0.01 | 0.05 | 0.00 | 0.03 | Height |
| rs2123731 | 19 | 4929473 | 0.99 | A | G | 0.03 | 0.00 | 0.01 | 0.03 | 0.12 | 0.05 | 0.03 | 0.06 | -0.05 | 0.04 | Height |
| rs891088 | 19 | 7184762 | 1.00 | G | A | 0.03 | 0.00 | -0.03 | 0.03 | -0.09 | 0.05 | -0.03 | 0.06 | 0.00 | 0.04 | Height |
| rs1346490 | 19 | 7244233 | 0.98 | A | C | 0.02 | 0.00 | -0.04 | 0.02 | 0.00 | 0.05 | -0.05 | 0.05 | -0.05 | 0.03 | Height |
| rs6511689 | 19 | 10321089 | 1.00 | T | C | 0.02 | 0.00 | -0.01 | 0.03 | -0.04 | 0.05 | 0.05 | 0.05 | -0.01 | 0.04 | Height |
| rs8102380 | 19 | 10801185 | 0.99 | G | A | 0.02 | 0.00 | -0.03 | 0.03 | -0.07 | 0.05 | -0.05 | 0.05 | 0.00 | 0.04 | Height |
| rs7259684 | 19 | 12186611 | 1.00 | G | A | 0.04 | 0.01 | 0.06 | 0.04 | 0.11 | 0.09 | -0.03 | 0.09 | 0.08 | 0.06 | Height |
| rs8103068 | 19 | 17522869 | 1.00 | T | C | 0.03 | 0.00 | 0.05 | 0.03 | 0.00 | 0.07 | 0.15 | 0.07 | 0.03 | 0.05 | Height |
| rs10401193 | 19 | 19591066 | 1.00 | A | G | 0.02 | 0.00 | 0.02 | 0.03 | 0.03 | 0.06 | 0.08 | 0.06 | -0.01 | 0.04 | Height |
| rs8103992 | 19 | 19665643 | 0.98 | A | C | 0.02 | 0.00 | -0.02 | 0.03 | -0.04 | 0.06 | 0.11 | 0.06 | -0.07 | 0.04 | Height |
| rs7253628 | 19 | 31047269 | 0.99 | G | A | 0.02 | 0.00 | 0.04 | 0.03 | 0.11 | 0.06 | 0.05 | 0.07 | 0.00 | 0.05 | Height |
| rs4802134 | 19 | 38346685 | 1.00 | A | G | 0.03 | 0.00 | -0.01 | 0.03 | -0.01 | 0.06 | -0.01 | 0.06 | -0.01 | 0.04 | Height |
| rs4803468 | 19 | 41922352 | 1.00 | A | G | 0.03 | 0.00 | 0.02 | 0.02 | -0.05 | 0.05 | 0.09 | 0.05 | 0.03 | 0.03 | Height |
| rs11880124 | 19 | 42683791 | 1.00 | A | G | 0.04 | 0.01 | -0.05 | 0.04 | -0.13 | 0.09 | -0.02 | 0.09 | -0.04 | 0.06 | Height |
| rs2682587 | 19 | 44082429 | 1.00 | A | C | 0.02 | 0.00 | 0.02 | 0.03 | -0.03 | 0.06 | 0.08 | 0.06 | 0.02 | 0.04 | Height |
| rs2059877 | 19 | 48188809 | 0.99 | T | G | 0.02 | 0.00 | -0.03 | 0.03 | -0.02 | 0.05 | 0.00 | 0.06 | -0.05 | 0.04 | Height |
| rs7273787 | 20 | 4098567 | 1.00 | G | A | 0.02 | 0.00 | 0.02 | 0.03 | 0.02 | 0.05 | -0.02 | 0.05 | 0.04 | 0.03 | Height |
| rs17721822 | 20 | 6469596 | 1.00 | G | A | 0.02 | 0.00 | -0.04 | 0.02 | -0.05 | 0.05 | -0.07 | 0.05 | -0.02 | 0.03 | Height |
| rs1884897 | 20 | 6612832 | 1.00 | A | G | 0.04 | 0.00 | 0.00 | 0.02 | -0.06 | 0.05 | 0.04 | 0.05 | 0.01 | 0.03 | Height |
| rs6080830 | 20 | 17771113 | 1.00 | A | G | 0.02 | 0.00 | 0.01 | 0.02 | 0.01 | 0.05 | -0.03 | 0.05 | 0.03 | 0.03 | Height |
| rs8117259 | 20 | 20348253 | 1.00 | C | T | 0.02 | 0.00 | -0.02 | 0.03 | -0.03 | 0.06 | -0.05 | 0.06 | -0.01 | 0.04 | Height |
| rs6137287 | 20 | 21180259 | 1.00 | T | C | 0.02 | 0.00 | 0.03 | 0.03 | 0.02 | 0.05 | 0.18 | 0.05 | -0.04 | 0.04 | Height |
| rs1535466 | 20 | 33718706 | 1.00 | G | A | 0.04 | 0.00 | 0.01 | 0.03 | 0.06 | 0.06 | 0.05 | 0.06 | -0.03 | 0.04 | Height |
| rs143384 | 20 | 34025756 | 0.95 | G | A | 0.06 | 0.00 | -0.03 | 0.02 | -0.13 | 0.05 | 0.00 | 0.05 | 0.01 | 0.03 | Height |
| rs2425163 | 20 | 34432670 | 1.00 | G | A | 0.04 | 0.00 | -0.01 | 0.03 | 0.00 | 0.06 | -0.06 | 0.06 | 0.01 | 0.04 | Height |
| rs4812586 | 20 | 35544673 | 1.00 | A | G | 0.03 | 0.00 | -0.01 | 0.03 | 0.05 | 0.07 | -0.03 | 0.07 | -0.02 | 0.05 | Height |
| rs2224538 | 20 | 38552078 | 1.00 | T | C | 0.02 | 0.00 | 0.01 | 0.02 | -0.05 | 0.05 | 0.00 | 0.05 | 0.04 | 0.03 | Height |
| rs6020202 | 20 | 48634821 | 0.98 | G | A | 0.02 | 0.00 | 0.02 | 0.03 | -0.01 | 0.06 | 0.08 | 0.06 | 0.01 | 0.04 | Height |
| rs1326023 | 20 | 54842378 | 1.00 | A | G | 0.02 | 0.00 | 0.02 | 0.03 | 0.02 | 0.05 | -0.01 | 0.05 | 0.03 | 0.04 | Height |
| rs2057291 | 20 | 57472043 | 0.92 | A | G | 0.02 | 0.00 | 0.05 | 0.03 | 0.06 | 0.05 | 0.11 | 0.06 | 0.02 | 0.03 | Height |
| rs3026499 | 20 | 57948773 | 0.83 | G | A | 0.03 | 0.00 | 0.02 | 0.03 | 0.00 | 0.06 | 0.04 | 0.06 | 0.02 | 0.03 | Height |
| rs6061231 | 20 | 60956917 | 1.00 | C | A | 0.02 | 0.00 | 0.01 | 0.03 | 0.07 | 0.05 | 0.06 | 0.06 | -0.04 | 0.04 | Height |
| rs2829941 | 21 | 27208935 | 0.99 | T | G | 0.02 | 0.00 | 0.00 | 0.02 | 0.05 | 0.05 | 0.01 | 0.05 | -0.02 | 0.03 | Height |
| rs2211866 | 21 | 39688107 | 0.99 | A | G | 0.02 | 0.00 | 0.02 | 0.02 | -0.03 | 0.05 | 0.05 | 0.05 | 0.02 | 0.03 | Height |
| rs9977276 | 21 | 47436327 | 0.99 | G | T | 0.02 | 0.00 | 0.01 | 0.03 | 0.00 | 0.06 | -0.04 | 0.06 | 0.04 | 0.04 | Height |
| rs7284476 | 22 | 38129332 | 1.00 | A | G | 0.02 | 0.00 | 0.00 | 0.02 | 0.08 | 0.05 | 0.00 | 0.05 | -0.04 | 0.03 | Height |
| rs738288 | 22 | 39907661 | 1.00 | G | A | 0.02 | 0.00 | -0.06 | 0.02 | -0.09 | 0.05 | -0.09 | 0.05 | -0.04 | 0.03 | Height |
| rs11090631 | 22 | 45846371 | 1.00 | T | C | 0.02 | 0.00 | 0.01 | 0.03 | -0.07 | 0.06 | 0.04 | 0.06 | 0.03 | 0.04 | Height |
| rs977747 | 1 | 47684677 | 1.00 | T | G | 0.02 | 0.00 | 0.02 | 0.02 | -0.03 | 0.05 | -0.05 | 0.05 | 0.07 | 0.03 | BMI |
| rs657452 | 1 | 49589847 | 1.00 | A | G | 0.02 | 0.00 | -0.03 | 0.02 | 0.07 | 0.05 | -0.08 | 0.05 | -0.06 | 0.03 | BMI |
| rs11583200 | 1 | 50559820 | 1.00 | C | T | 0.02 | 0.00 | -0.04 | 0.02 | 0.01 | 0.05 | -0.05 | 0.05 | -0.05 | 0.03 | BMI |
| rs3101336 | 1 | 72751185 | 1.00 | C | T | 0.03 | 0.00 | 0.03 | 0.02 | 0.04 | 0.05 | 0.08 | 0.05 | 0.00 | 0.03 | BMI |
| rs12566985 | 1 | 75002193 | 1.00 | G | A | 0.02 | 0.00 | 0.01 | 0.02 | -0.03 | 0.05 | 0.02 | 0.05 | 0.03 | 0.03 | BMI |
| rs12401738 | 1 | 78446761 | 0.99 | A | G | 0.02 | 0.00 | 0.02 | 0.03 | -0.04 | 0.05 | -0.03 | 0.05 | 0.07 | 0.03 | BMI |
| rs11165643 | 1 | 96924097 | 1.00 | T | C | 0.02 | 0.00 | 0.01 | 0.02 | 0.03 | 0.05 | -0.01 | 0.05 | 0.01 | 0.03 | BMI |
| rs17024393 | 1 | 110154688 | 0.99 | C | T | 0.07 | 0.01 | -0.12 | 0.07 | -0.17 | 0.14 | -0.24 | 0.16 | -0.05 | 0.09 | BMI |
| rs543874 | 1 | 177889480 | 1.00 | G | A | 0.05 | 0.00 | 0.02 | 0.03 | 0.06 | 0.06 | -0.05 | 0.06 | 0.02 | 0.04 | BMI |
| rs2820292 | 1 | 201784287 | 0.99 | C | A | 0.02 | 0.00 | 0.00 | 0.02 | 0.00 | 0.05 | -0.04 | 0.05 | 0.03 | 0.03 | BMI |
| rs13021737 | 2 | 632348 | 0.99 | G | A | 0.06 | 0.00 | 0.03 | 0.03 | -0.01 | 0.06 | 0.10 | 0.07 | 0.02 | 0.04 | BMI |
| rs10182181 | 2 | 25150296 | 1.00 | G | A | 0.03 | 0.00 | 0.02 | 0.02 | 0.09 | 0.05 | 0.00 | 0.05 | -0.01 | 0.03 | BMI |
| rs11126666 | 2 | 26928811 | 1.00 | A | G | 0.02 | 0.00 | 0.00 | 0.03 | -0.05 | 0.05 | 0.09 | 0.06 | -0.02 | 0.04 | BMI |
| rs1016287 | 2 | 59305625 | 1.00 | T | C | 0.02 | 0.00 | -0.01 | 0.03 | -0.06 | 0.05 | 0.04 | 0.05 | -0.02 | 0.04 | BMI |
| rs11688816 | 2 | 63053048 | 1.00 | G | A | 0.02 | 0.00 | -0.01 | 0.02 | -0.06 | 0.05 | 0.07 | 0.05 | -0.02 | 0.03 | BMI |
| rs2121279 | 2 | 143043285 | 1.00 | T | C | 0.03 | 0.00 | -0.01 | 0.04 | -0.01 | 0.07 | 0.06 | 0.07 | -0.04 | 0.05 | BMI |
| rs1460676 | 2 | 164567689 | 0.99 | C | T | 0.02 | 0.00 | -0.01 | 0.03 | 0.11 | 0.06 | 0.04 | 0.07 | -0.09 | 0.04 | BMI |
| rs1528435 | 2 | 181550962 | 1.00 | T | C | 0.02 | 0.00 | 0.01 | 0.02 | 0.05 | 0.05 | 0.01 | 0.05 | -0.01 | 0.03 | BMI |
| rs17203016 | 2 | 208255518 | 1.00 | G | A | 0.02 | 0.00 | 0.06 | 0.03 | 0.01 | 0.06 | 0.15 | 0.06 | 0.05 | 0.04 | BMI |
| rs7599312 | 2 | 213413231 | 1.00 | G | A | 0.02 | 0.00 | 0.05 | 0.03 | 0.05 | 0.05 | -0.05 | 0.06 | 0.08 | 0.04 | BMI |
| rs492400 | 2 | 219349752 | 0.99 | C | T | 0.02 | 0.00 | 0.01 | 0.02 | 0.01 | 0.05 | 0.04 | 0.05 | 0.00 | 0.03 | BMI |
| rs2176040 | 2 | 227092802 | 1.00 | A | G | 0.01 | 0.00 | 0.00 | 0.02 | 0.03 | 0.05 | -0.04 | 0.05 | 0.01 | 0.03 | BMI |
| rs6804842 | 3 | 25106437 | 1.00 | G | A | 0.02 | 0.00 | 0.00 | 0.02 | -0.02 | 0.05 | 0.02 | 0.05 | 0.00 | 0.03 | BMI |
| rs2365389 | 3 | 61236462 | 0.99 | C | T | 0.02 | 0.00 | 0.06 | 0.02 | 0.07 | 0.05 | 0.14 | 0.05 | 0.02 | 0.03 | BMI |
| rs3849570 | 3 | 81792112 | 1.00 | A | C | 0.02 | 0.00 | 0.03 | 0.03 | 0.01 | 0.05 | -0.01 | 0.05 | 0.06 | 0.03 | BMI |
| rs13078960 | 3 | 85807590 | 1.00 | G | T | 0.03 | 0.00 | 0.05 | 0.03 | 0.13 | 0.06 | 0.03 | 0.06 | 0.02 | 0.04 | BMI |
| rs16851483 | 3 | 141275436 | 1.00 | T | G | 0.05 | 0.01 | 0.03 | 0.05 | -0.10 | 0.10 | 0.20 | 0.10 | 0.02 | 0.07 | BMI |
| rs1516725 | 3 | 185824004 | 0.99 | C | T | 0.05 | 0.01 | 0.00 | 0.04 | -0.18 | 0.07 | 0.02 | 0.07 | 0.08 | 0.05 | BMI |
| rs10938397 | 4 | 45182527 | 1.00 | G | A | 0.04 | 0.00 | 0.01 | 0.02 | 0.15 | 0.05 | -0.10 | 0.05 | -0.01 | 0.03 | BMI |
| rs13107325 | 4 | 103188709 | 0.99 | T | C | 0.05 | 0.01 | -0.11 | 0.05 | -0.08 | 0.10 | -0.07 | 0.10 | -0.14 | 0.06 | BMI |
| rs11727676 | 4 | 145659064 | 0.84 | T | C | 0.04 | 0.01 | 0.04 | 0.04 | 0.15 | 0.09 | -0.02 | 0.10 | 0.03 | 0.05 | BMI |
| rs2112347 | 5 | 75015242 | 0.99 | T | G | 0.03 | 0.00 | 0.02 | 0.02 | -0.05 | 0.05 | 0.06 | 0.05 | 0.04 | 0.03 | BMI |
| rs7715256 | 5 | 153537893 | 1.00 | G | T | 0.02 | 0.00 | -0.01 | 0.02 | -0.04 | 0.05 | -0.02 | 0.05 | 0.01 | 0.03 | BMI |
| rs205262 | 6 | 34563164 | 1.00 | G | A | 0.02 | 0.00 | 0.05 | 0.03 | 0.05 | 0.05 | 0.08 | 0.06 | 0.02 | 0.04 | BMI |
| rs2033529 | 6 | 40348653 | 1.00 | G | A | 0.02 | 0.00 | -0.06 | 0.03 | -0.09 | 0.05 | -0.01 | 0.05 | -0.06 | 0.04 | BMI |
| rs2207139 | 6 | 50845490 | 1.00 | G | A | 0.05 | 0.00 | 0.04 | 0.03 | 0.04 | 0.06 | 0.08 | 0.07 | 0.02 | 0.04 | BMI |
| rs9400239 | 6 | 108977663 | 0.99 | C | T | 0.02 | 0.00 | 0.04 | 0.03 | 0.10 | 0.05 | -0.02 | 0.05 | 0.05 | 0.04 | BMI |
| rs9374842 | 6 | 120185665 | 1.00 | T | C | 0.02 | 0.00 | -0.02 | 0.03 | -0.04 | 0.05 | 0.01 | 0.06 | -0.02 | 0.04 | BMI |
| rs13201877 | 6 | 137675541 | 0.94 | G | A | 0.02 | 0.01 | -0.03 | 0.04 | -0.02 | 0.07 | -0.19 | 0.08 | 0.04 | 0.05 | BMI |
| rs13191362 | 6 | 163033350 | 0.99 | A | G | 0.03 | 0.01 | -0.05 | 0.04 | -0.05 | 0.08 | -0.04 | 0.08 | -0.05 | 0.05 | BMI |
| rs1167827 | 7 | 75163169 | 1.00 | G | A | 0.02 | 0.00 | 0.06 | 0.02 | -0.02 | 0.05 | 0.09 | 0.05 | 0.09 | 0.03 | BMI |
| rs2245368 | 7 | 76608143 | 0.94 | C | T | 0.03 | 0.01 | 0.02 | 0.03 | -0.07 | 0.06 | 0.01 | 0.07 | 0.06 | 0.04 | BMI |
| rs10464483 | 7 | 93193242 | 1.00 | T | C | 0.02 | 0.00 | -0.02 | 0.02 | -0.03 | 0.05 | 0.05 | 0.05 | -0.04 | 0.03 | BMI |
| rs6465468 | 7 | 95169514 | 0.98 | T | G | 0.02 | 0.00 | 0.00 | 0.03 | -0.06 | 0.05 | 0.02 | 0.05 | 0.02 | 0.04 | BMI |
| rs17405819 | 8 | 76806584 | 1.00 | T | C | 0.02 | 0.00 | 0.05 | 0.03 | 0.04 | 0.05 | 0.00 | 0.05 | 0.07 | 0.04 | BMI |
| rs16907751 | 8 | 81375457 | 0.98 | C | T | 0.04 | 0.01 | -0.04 | 0.04 | -0.14 | 0.08 | 0.06 | 0.08 | -0.03 | 0.05 | BMI |
| rs2033732 | 8 | 85079709 | 0.99 | C | T | 0.02 | 0.00 | -0.01 | 0.03 | 0.03 | 0.06 | 0.00 | 0.06 | -0.02 | 0.04 | BMI |
| rs4740619 | 9 | 15634326 | 1.00 | T | C | 0.02 | 0.00 | -0.03 | 0.02 | 0.06 | 0.05 | -0.09 | 0.05 | -0.04 | 0.03 | BMI |
| rs10968576 | 9 | 28414339 | 1.00 | G | A | 0.03 | 0.00 | 0.00 | 0.03 | -0.01 | 0.05 | 0.04 | 0.05 | -0.01 | 0.04 | BMI |
| rs6477694 | 9 | 111932342 | 1.00 | C | T | 0.02 | 0.00 | 0.01 | 0.02 | 0.03 | 0.05 | 0.05 | 0.05 | -0.01 | 0.03 | BMI |
| rs1928295 | 9 | 120378483 | 1.00 | T | C | 0.02 | 0.00 | 0.08 | 0.02 | 0.02 | 0.05 | 0.11 | 0.05 | 0.10 | 0.03 | BMI |
| rs10733682 | 9 | 129460914 | 1.00 | A | G | 0.02 | 0.00 | 0.03 | 0.02 | 0.11 | 0.05 | 0.02 | 0.05 | 0.01 | 0.03 | BMI |
| rs7899106 | 10 | 87410904 | 1.00 | G | A | 0.04 | 0.01 | 0.07 | 0.06 | -0.01 | 0.11 | 0.16 | 0.12 | 0.07 | 0.08 | BMI |
| rs17094222 | 10 | 102395440 | 0.98 | C | T | 0.03 | 0.00 | -0.03 | 0.03 | 0.00 | 0.06 | -0.02 | 0.06 | -0.04 | 0.04 | BMI |
| rs11191560 | 10 | 104869038 | 1.00 | C | T | 0.03 | 0.01 | 0.01 | 0.04 | 0.00 | 0.08 | -0.03 | 0.09 | 0.04 | 0.06 | BMI |
| rs7903146 | 10 | 114758349 | 1.00 | C | T | 0.02 | 0.00 | -0.01 | 0.03 | 0.04 | 0.05 | -0.03 | 0.05 | -0.02 | 0.04 | BMI |
| rs2316901 | 11 | 8679016 | 1.00 | G | A | 0.02 | 0.00 | -0.05 | 0.02 | -0.07 | 0.05 | 0.02 | 0.05 | -0.08 | 0.03 | BMI |
| rs11030104 | 11 | 27684517 | 1.00 | A | G | 0.04 | 0.00 | -0.08 | 0.03 | -0.18 | 0.06 | 0.05 | 0.06 | -0.09 | 0.04 | BMI |
| rs2176598 | 11 | 43864278 | 1.00 | T | C | 0.02 | 0.00 | -0.04 | 0.03 | -0.03 | 0.05 | 0.00 | 0.06 | -0.06 | 0.04 | BMI |
| rs3817334 | 11 | 47650993 | 1.00 | T | C | 0.03 | 0.00 | 0.03 | 0.02 | 0.06 | 0.05 | 0.03 | 0.05 | 0.01 | 0.03 | BMI |
| rs12286929 | 11 | 115022404 | 0.99 | G | A | 0.02 | 0.00 | 0.03 | 0.02 | 0.03 | 0.05 | 0.04 | 0.05 | 0.03 | 0.03 | BMI |
| rs7138803 | 12 | 50247468 | 1.00 | A | G | 0.03 | 0.00 | -0.01 | 0.02 | 0.03 | 0.05 | -0.07 | 0.05 | 0.00 | 0.03 | BMI |
| rs11057405 | 12 | 122781897 | 1.00 | G | A | 0.03 | 0.01 | 0.05 | 0.04 | 0.14 | 0.08 | 0.11 | 0.09 | -0.01 | 0.05 | BMI |
| rs9581854 | 13 | 28017782 | 0.99 | T | C | 0.03 | 0.01 | -0.04 | 0.03 | 0.02 | 0.06 | -0.03 | 0.07 | -0.07 | 0.04 | BMI |
| rs12429545 | 13 | 54102206 | 1.00 | A | G | 0.03 | 0.01 | -0.03 | 0.04 | -0.08 | 0.07 | 0.09 | 0.07 | -0.06 | 0.05 | BMI |
| rs9540493 | 13 | 66205704 | 0.98 | A | G | 0.02 | 0.00 | -0.02 | 0.02 | 0.00 | 0.05 | 0.01 | 0.05 | -0.04 | 0.03 | BMI |
| rs1441264 | 13 | 79580919 | 1.00 | A | G | 0.02 | 0.00 | 0.05 | 0.02 | 0.03 | 0.05 | 0.08 | 0.05 | 0.05 | 0.03 | BMI |
| rs10132280 | 14 | 25928179 | 0.99 | C | A | 0.02 | 0.00 | 0.07 | 0.03 | 0.08 | 0.05 | 0.08 | 0.05 | 0.06 | 0.04 | BMI |
| rs12885454 | 14 | 29736838 | 0.99 | C | A | 0.02 | 0.00 | -0.02 | 0.03 | -0.05 | 0.05 | -0.05 | 0.05 | 0.02 | 0.03 | BMI |
| rs11847697 | 14 | 30515112 | 0.98 | T | C | 0.05 | 0.01 | 0.03 | 0.06 | 0.01 | 0.13 | -0.01 | 0.13 | 0.05 | 0.08 | BMI |
| rs7141420 | 14 | 79899454 | 1.00 | T | C | 0.02 | 0.00 | 0.04 | 0.02 | -0.03 | 0.05 | 0.05 | 0.05 | 0.06 | 0.03 | BMI |
| rs3736485 | 15 | 51748610 | 0.99 | A | G | 0.02 | 0.00 | -0.01 | 0.02 | 0.02 | 0.05 | -0.06 | 0.05 | 0.00 | 0.03 | BMI |
| rs16951275 | 15 | 68077168 | 1.00 | T | C | 0.03 | 0.00 | 0.02 | 0.03 | -0.05 | 0.06 | 0.11 | 0.06 | 0.01 | 0.04 | BMI |
| rs7164727 | 15 | 73093991 | 1.00 | T | C | 0.02 | 0.00 | -0.09 | 0.03 | -0.09 | 0.05 | -0.10 | 0.05 | -0.09 | 0.04 | BMI |
| rs12446632 | 16 | 19935389 | 1.00 | G | A | 0.04 | 0.01 | 0.04 | 0.03 | 0.09 | 0.07 | 0.02 | 0.07 | 0.02 | 0.05 | BMI |
| rs2650492 | 16 | 28333411 | 1.00 | A | G | 0.02 | 0.00 | -0.02 | 0.03 | 0.05 | 0.05 | -0.09 | 0.06 | -0.01 | 0.04 | BMI |
| rs3888190 | 16 | 28889486 | 1.00 | A | C | 0.03 | 0.00 | 0.01 | 0.02 | 0.05 | 0.05 | -0.05 | 0.05 | 0.02 | 0.03 | BMI |
| rs4787491 | 16 | 30015337 | 0.99 | G | A | 0.02 | 0.00 | -0.01 | 0.02 | 0.03 | 0.05 | -0.02 | 0.05 | -0.03 | 0.03 | BMI |
| rs9925964 | 16 | 31129895 | 1.00 | A | G | 0.02 | 0.00 | 0.03 | 0.02 | 0.01 | 0.05 | -0.03 | 0.05 | 0.06 | 0.03 | BMI |
| rs2080454 | 16 | 49062590 | 1.00 | C | A | 0.02 | 0.00 | -0.04 | 0.02 | 0.03 | 0.05 | 0.00 | 0.05 | -0.08 | 0.03 | BMI |
| rs1421085 | 16 | 53800954 | 1.00 | C | T | 0.08 | 0.00 | 0.06 | 0.02 | 0.11 | 0.05 | 0.01 | 0.05 | 0.05 | 0.03 | BMI |
| rs8082647 | 17 | 2010597 | 1.00 | C | T | 0.01 | 0.00 | 0.03 | 0.03 | 0.00 | 0.06 | 0.05 | 0.06 | 0.04 | 0.04 | BMI |
| rs1000940 | 17 | 5283252 | 1.00 | G | A | 0.02 | 0.00 | 0.00 | 0.03 | -0.05 | 0.05 | 0.01 | 0.05 | 0.02 | 0.04 | BMI |
| rs12940622 | 17 | 78615571 | 1.00 | G | A | 0.02 | 0.00 | -0.04 | 0.02 | 0.04 | 0.05 | -0.12 | 0.05 | -0.05 | 0.03 | BMI |
| rs1808579 | 18 | 21104888 | 1.00 | C | T | 0.02 | 0.00 | 0.03 | 0.02 | 0.09 | 0.05 | 0.14 | 0.05 | -0.03 | 0.03 | BMI |
| rs7239883 | 18 | 40147671 | 1.00 | G | A | 0.02 | 0.00 | 0.02 | 0.02 | -0.08 | 0.05 | 0.01 | 0.05 | 0.07 | 0.03 | BMI |
| rs7243357 | 18 | 56883319 | 0.99 | T | G | 0.02 | 0.00 | 0.14 | 0.03 | 0.18 | 0.06 | 0.18 | 0.07 | 0.10 | 0.04 | BMI |
| rs6567160 | 18 | 57829135 | 0.99 | C | T | 0.06 | 0.00 | 0.03 | 0.03 | 0.05 | 0.06 | 0.01 | 0.06 | 0.04 | 0.04 | BMI |
| rs17724992 | 19 | 18454825 | 1.00 | A | G | 0.02 | 0.00 | -0.04 | 0.03 | -0.07 | 0.05 | -0.06 | 0.06 | -0.01 | 0.04 | BMI |
| rs29941 | 19 | 34309532 | 1.00 | G | A | 0.02 | 0.00 | -0.02 | 0.03 | -0.03 | 0.05 | -0.12 | 0.05 | 0.04 | 0.04 | BMI |
| rs2075650 | 19 | 45395619 | 1.00 | A | G | 0.03 | 0.01 | -0.06 | 0.03 | -0.15 | 0.07 | -0.06 | 0.07 | -0.02 | 0.05 | BMI |
| rs2287019 | 19 | 46202172 | 1.00 | C | T | 0.04 | 0.00 | 0.02 | 0.03 | 0.01 | 0.06 | 0.05 | 0.06 | 0.00 | 0.04 | BMI |
| rs3810291 | 19 | 47569003 | 0.91 | A | G | 0.03 | 0.00 | -0.06 | 0.03 | -0.01 | 0.05 | -0.11 | 0.06 | -0.05 | 0.03 | BMI |
| rs6091540 | 20 | 51087862 | 1.00 | C | T | 0.02 | 0.00 | 0.00 | 0.03 | -0.01 | 0.05 | 0.05 | 0.06 | -0.01 | 0.04 | BMI |
| rs2836754 | 21 | 40291740 | 1.00 | C | T | 0.02 | 0.00 | 0.05 | 0.02 | 0.01 | 0.05 | 0.08 | 0.05 | 0.06 | 0.03 | BMI |
| rs2765539 | 1 | 119549418 | 1.00 | T | C | 0.03 | 0.00 | -0.03 | 0.03 | -0.08 | 0.06 | -0.03 | 0.06 | -0.02 | 0.04 | Waist-to-hip ratio |
| rs1011731 | 1 | 172346548 | 1.00 | G | A | 0.02 | 0.00 | 0.04 | 0.02 | -0.01 | 0.05 | 0.03 | 0.05 | 0.06 | 0.03 | Waist-to-hip ratio |
| rs1563355 | 1 | 219653101 | 1.00 | C | T | 0.03 | 0.00 | 0.06 | 0.03 | 0.02 | 0.05 | 0.06 | 0.05 | 0.08 | 0.04 | Waist-to-hip ratio |
| rs929641 | 2 | 58792377 | 1.00 | A | G | 0.02 | 0.00 | 0.02 | 0.02 | -0.02 | 0.05 | 0.01 | 0.05 | 0.04 | 0.03 | Waist-to-hip ratio |
| rs1128249 | 2 | 165528624 | 0.99 | G | T | 0.02 | 0.00 | 0.02 | 0.02 | 0.06 | 0.05 | 0.08 | 0.05 | -0.02 | 0.03 | Waist-to-hip ratio |
| rs1569135 | 2 | 188115398 | 1.00 | A | G | 0.02 | 0.00 | -0.05 | 0.02 | -0.04 | 0.05 | -0.06 | 0.05 | -0.05 | 0.03 | Waist-to-hip ratio |
| rs2972164 | 3 | 12334416 | 0.99 | C | T | 0.02 | 0.00 | -0.03 | 0.02 | -0.08 | 0.05 | 0.00 | 0.05 | -0.02 | 0.03 | Waist-to-hip ratio |
| rs904453 | 3 | 12704894 | 1.00 | T | G | 0.02 | 0.00 | -0.05 | 0.02 | -0.09 | 0.05 | -0.12 | 0.05 | 0.00 | 0.03 | Waist-to-hip ratio |
| rs9860730 | 3 | 64701146 | 0.99 | A | G | 0.02 | 0.00 | 0.03 | 0.03 | 0.10 | 0.05 | 0.02 | 0.05 | 0.00 | 0.04 | Waist-to-hip ratio |
| rs17451107 | 3 | 156797609 | 0.98 | T | C | 0.02 | 0.00 | 0.03 | 0.02 | 0.01 | 0.05 | 0.04 | 0.05 | 0.04 | 0.03 | Waist-to-hip ratio |
| rs459193 | 5 | 55806751 | 0.98 | A | G | 0.03 | 0.00 | -0.02 | 0.03 | -0.11 | 0.06 | 0.07 | 0.06 | -0.02 | 0.04 | Waist-to-hip ratio |
| rs1294421 | 6 | 6743149 | 0.99 | G | T | 0.03 | 0.00 | -0.01 | 0.02 | -0.01 | 0.05 | -0.06 | 0.05 | 0.01 | 0.03 | Waist-to-hip ratio |
| rs11755724 | 6 | 7118990 | 1.00 | G | A | 0.02 | 0.00 | -0.02 | 0.02 | -0.08 | 0.05 | 0.04 | 0.05 | -0.01 | 0.03 | Waist-to-hip ratio |
| rs998584 | 6 | 43757896 | 0.92 | A | C | 0.03 | 0.00 | 0.03 | 0.02 | 0.04 | 0.05 | 0.05 | 0.05 | 0.03 | 0.03 | Waist-to-hip ratio |
| rs2207139 | 6 | 50845490 | 1.00 | G | A | 0.03 | 0.00 | 0.04 | 0.03 | 0.04 | 0.06 | 0.08 | 0.07 | 0.02 | 0.04 | Waist-to-hip ratio |
| rs2745359 | 6 | 127381956 | 0.90 | C | T | 0.06 | 0.01 | -0.01 | 0.05 | -0.04 | 0.11 | -0.02 | 0.12 | 0.00 | 0.07 | Waist-to-hip ratio |
| rs10245353 | 7 | 25858614 | 0.99 | A | C | 0.03 | 0.00 | -0.01 | 0.03 | 0.01 | 0.06 | -0.05 | 0.06 | 0.00 | 0.04 | Waist-to-hip ratio |
| rs7801581 | 7 | 27223771 | 0.99 | T | C | 0.02 | 0.00 | 0.02 | 0.03 | -0.06 | 0.06 | 0.08 | 0.06 | 0.02 | 0.04 | Waist-to-hip ratio |
| rs12549058 | 8 | 72492238 | 1.00 | G | T | 0.04 | 0.01 | 0.02 | 0.05 | 0.07 | 0.09 | -0.01 | 0.10 | 0.00 | 0.07 | Waist-to-hip ratio |
| rs4929927 | 11 | 8658485 | 1.00 | G | A | 0.02 | 0.00 | -0.05 | 0.02 | -0.07 | 0.05 | 0.02 | 0.05 | -0.08 | 0.03 | Waist-to-hip ratio |
| rs11048470 | 12 | 26487283 | 1.00 | T | G | 0.03 | 0.00 | -0.02 | 0.03 | -0.01 | 0.05 | -0.06 | 0.06 | 0.00 | 0.04 | Waist-to-hip ratio |
| rs10783615 | 12 | 54349773 | 0.95 | G | A | 0.04 | 0.00 | -0.04 | 0.03 | 0.03 | 0.07 | -0.06 | 0.07 | -0.07 | 0.05 | Waist-to-hip ratio |
| rs10876528 | 12 | 54421476 | 0.99 | A | C | 0.03 | 0.00 | 0.03 | 0.02 | 0.07 | 0.05 | -0.03 | 0.05 | 0.05 | 0.03 | Waist-to-hip ratio |
| rs1316952 | 12 | 124399550 | 1.00 | T | C | 0.03 | 0.00 | -0.02 | 0.03 | -0.05 | 0.07 | 0.10 | 0.07 | -0.05 | 0.05 | Waist-to-hip ratio |
| rs17109256 | 14 | 79939993 | 1.00 | A | G | 0.02 | 0.00 | 0.05 | 0.03 | -0.01 | 0.06 | 0.12 | 0.06 | 0.04 | 0.04 | Waist-to-hip ratio |
| rs1440372 | 15 | 67033151 | 0.99 | C | T | 0.02 | 0.00 | -0.01 | 0.03 | 0.02 | 0.05 | -0.04 | 0.05 | 0.00 | 0.04 | Waist-to-hip ratio |
| rs1121980 | 16 | 53809247 | 1.00 | A | G | 0.04 | 0.00 | 0.06 | 0.02 | 0.10 | 0.05 | 0.02 | 0.05 | 0.06 | 0.03 | Waist-to-hip ratio |
| rs4640244 | 17 | 21284223 | 1.00 | G | A | 0.02 | 0.00 | -0.03 | 0.02 | -0.04 | 0.05 | 0.05 | 0.05 | -0.05 | 0.03 | Waist-to-hip ratio |
| rs11663816 | 18 | 57876227 | 1.00 | C | T | 0.03 | 0.00 | 0.02 | 0.03 | 0.04 | 0.05 | -0.01 | 0.06 | 0.02 | 0.04 | Waist-to-hip ratio |
| rs3786897 | 19 | 33893008 | 0.99 | G | A | 0.02 | 0.00 | -0.03 | 0.02 | -0.05 | 0.05 | -0.09 | 0.05 | 0.01 | 0.03 | Waist-to-hip ratio |
| rs2075650 | 19 | 45395619 | 1.00 | A | G | 0.03 | 0.00 | -0.06 | 0.03 | -0.15 | 0.07 | -0.06 | 0.07 | -0.02 | 0.05 | Waist-to-hip ratio |
| rs2287019 | 19 | 46202172 | 1.00 | C | T | 0.03 | 0.00 | 0.02 | 0.03 | 0.01 | 0.06 | 0.05 | 0.06 | 0.00 | 0.04 | Waist-to-hip ratio |
| rs16996700 | 20 | 50981945 | 1.00 | T | C | 0.02 | 0.00 | 0.01 | 0.03 | 0.06 | 0.05 | -0.04 | 0.05 | 0.02 | 0.04 | Waist-to-hip ratio |
| rs2179129 | 22 | 29450923 | 1.00 | A | G | 0.02 | 0.00 | -0.02 | 0.02 | -0.01 | 0.05 | 0.02 | 0.05 | -0.05 | 0.03 | Waist-to-hip ratio |
| rs12748152 | 1 | 27138393 | 0.99 | C | T | 0.05 | 0.01 | 0.05 | 0.04 | -0.01 | 0.09 | 0.04 | 0.10 | 0.07 | 0.06 | HDL |
| rs4660293 | 1 | 40028180 | 1.00 | A | G | 0.04 | 0.00 | -0.04 | 0.03 | 0.03 | 0.06 | -0.10 | 0.06 | -0.04 | 0.04 | HDL |
| rs12145743 | 1 | 156700651 | 1.00 | G | T | 0.02 | 0.00 | 0.00 | 0.02 | -0.05 | 0.05 | 0.06 | 0.05 | -0.01 | 0.03 | HDL |
| rs4650994 | 1 | 178515312 | 1.00 | G | A | 0.02 | 0.00 | 0.02 | 0.02 | -0.05 | 0.05 | 0.03 | 0.05 | 0.05 | 0.03 | HDL |
| rs1689800 | 1 | 182168885 | 0.99 | A | G | 0.03 | 0.00 | -0.03 | 0.02 | -0.01 | 0.05 | -0.13 | 0.05 | 0.01 | 0.03 | HDL |
| rs4846914 | 1 | 230295691 | 0.98 | A | G | 0.05 | 0.00 | 0.03 | 0.02 | -0.04 | 0.05 | -0.02 | 0.05 | 0.09 | 0.03 | HDL |
| rs1042034 | 2 | 21225281 | 1.00 | C | T | 0.07 | 0.00 | 0.04 | 0.03 | -0.01 | 0.06 | 0.07 | 0.06 | 0.06 | 0.04 | HDL |
| rs12328675 | 2 | 165540800 | 0.99 | C | T | 0.04 | 0.01 | -0.01 | 0.04 | -0.09 | 0.07 | 0.05 | 0.07 | 0.01 | 0.05 | HDL |
| rs1047891 | 2 | 211540507 | 0.90 | C | A | 0.03 | 0.00 | 0.01 | 0.03 | 0.02 | 0.05 | 0.02 | 0.06 | 0.01 | 0.04 | HDL |
| rs2972146 | 2 | 227100698 | 1.00 | G | T | 0.03 | 0.00 | 0.01 | 0.02 | 0.03 | 0.05 | -0.03 | 0.05 | 0.01 | 0.03 | HDL |
| rs2606736 | 3 | 11400249 | 0.99 | C | T | 0.02 | 0.00 | -0.03 | 0.02 | 0.04 | 0.05 | -0.09 | 0.05 | -0.03 | 0.03 | HDL |
| rs2290547 | 3 | 47061183 | 1.00 | G | A | 0.03 | 0.00 | 0.02 | 0.03 | -0.09 | 0.06 | 0.07 | 0.07 | 0.05 | 0.04 | HDL |
| rs2013208 | 3 | 50129399 | 1.00 | T | C | 0.03 | 0.00 | -0.02 | 0.02 | 0.00 | 0.05 | -0.01 | 0.05 | -0.03 | 0.03 | HDL |
| rs13326165 | 3 | 52532118 | 1.00 | A | G | 0.03 | 0.00 | -0.02 | 0.03 | 0.00 | 0.06 | -0.08 | 0.06 | -0.01 | 0.04 | HDL |
| rs6805251 | 3 | 119560606 | 1.00 | T | C | 0.02 | 0.00 | 0.03 | 0.02 | 0.05 | 0.05 | -0.01 | 0.05 | 0.04 | 0.03 | HDL |
| rs17404153 | 3 | 132163200 | 1.00 | T | G | 0.01 | 0.01 | 0.02 | 0.04 | 0.02 | 0.07 | 0.02 | 0.08 | 0.03 | 0.05 | HDL |
| rs10019888 | 4 | 26062990 | 0.99 | A | G | 0.03 | 0.00 | 0.02 | 0.03 | -0.03 | 0.06 | 0.03 | 0.07 | 0.05 | 0.04 | HDL |
| rs3822072 | 4 | 89741269 | 0.99 | G | A | 0.03 | 0.00 | 0.00 | 0.02 | -0.05 | 0.05 | -0.01 | 0.05 | 0.02 | 0.03 | HDL |
| rs2602836 | 4 | 100014805 | 1.00 | A | G | 0.02 | 0.00 | 0.01 | 0.02 | 0.03 | 0.05 | -0.01 | 0.05 | 0.01 | 0.03 | HDL |
| rs13107325 | 4 | 103188709 | 0.99 | C | T | 0.07 | 0.01 | 0.11 | 0.05 | 0.08 | 0.10 | 0.07 | 0.10 | 0.14 | 0.06 | HDL |
| rs6450176 | 5 | 53298025 | 1.00 | G | A | 0.03 | 0.00 | -0.04 | 0.03 | 0.00 | 0.06 | -0.09 | 0.06 | -0.04 | 0.04 | HDL |
| rs2814944 | 6 | 34552797 | 1.00 | G | A | 0.03 | 0.00 | -0.01 | 0.03 | 0.01 | 0.06 | -0.05 | 0.07 | -0.01 | 0.05 | HDL |
| rs998584 | 6 | 43757896 | 0.92 | C | A | 0.03 | 0.00 | -0.03 | 0.02 | -0.04 | 0.05 | -0.05 | 0.05 | -0.03 | 0.03 | HDL |
| rs1936800 | 6 | 127436064 | 0.99 | C | T | 0.02 | 0.00 | -0.05 | 0.02 | -0.05 | 0.05 | -0.09 | 0.05 | -0.03 | 0.03 | HDL |
| rs605066 | 6 | 139829666 | 0.99 | T | C | 0.03 | 0.00 | 0.04 | 0.02 | 0.08 | 0.05 | -0.05 | 0.05 | 0.06 | 0.03 | HDL |
| rs702485 | 7 | 6449272 | 1.00 | G | A | 0.02 | 0.00 | 0.00 | 0.02 | 0.06 | 0.05 | -0.05 | 0.05 | -0.01 | 0.03 | HDL |
| rs4142995 | 7 | 17919258 | 1.00 | G | T | 0.03 | 0.00 | 0.00 | 0.02 | -0.01 | 0.05 | -0.06 | 0.05 | 0.03 | 0.03 | HDL |
| rs4917014 | 7 | 50305863 | 1.00 | G | T | 0.02 | 0.00 | 0.02 | 0.03 | 0.03 | 0.05 | 0.02 | 0.05 | 0.01 | 0.03 | HDL |
| rs17145738 | 7 | 72982874 | 0.98 | T | C | 0.04 | 0.01 | 0.06 | 0.04 | 0.08 | 0.07 | -0.01 | 0.08 | 0.09 | 0.05 | HDL |
| rs4731702 | 7 | 130433384 | 1.00 | T | C | 0.03 | 0.00 | -0.02 | 0.02 | -0.01 | 0.05 | -0.10 | 0.05 | 0.01 | 0.03 | HDL |
| rs17173637 | 7 | 150529449 | 1.00 | T | C | 0.04 | 0.01 | -0.02 | 0.04 | 0.01 | 0.08 | 0.07 | 0.08 | -0.08 | 0.06 | HDL |
| rs9987289 | 8 | 9183358 | 1.00 | G | A | 0.08 | 0.01 | 0.14 | 0.04 | 0.26 | 0.08 | -0.02 | 0.09 | 0.15 | 0.06 | HDL |
| rs12678919 | 8 | 19844222 | 1.00 | G | A | 0.16 | 0.01 | 0.00 | 0.04 | 0.02 | 0.08 | 0.07 | 0.08 | -0.04 | 0.05 | HDL |
| rs2293889 | 8 | 116599199 | 1.00 | G | T | 0.03 | 0.00 | -0.01 | 0.02 | -0.12 | 0.05 | 0.04 | 0.05 | 0.01 | 0.03 | HDL |
| rs638491 | 9 | 15290012 | 1.00 | G | A | 0.04 | 0.00 | 0.00 | 0.03 | -0.03 | 0.06 | 0.01 | 0.07 | 0.00 | 0.04 | HDL |
| rs1883025 | 9 | 107664301 | 0.99 | C | T | 0.07 | 0.00 | 0.02 | 0.03 | 0.02 | 0.06 | 0.03 | 0.06 | 0.02 | 0.04 | HDL |
| rs970548 | 10 | 46013277 | 1.00 | C | A | 0.03 | 0.00 | -0.07 | 0.03 | -0.15 | 0.05 | -0.09 | 0.06 | -0.02 | 0.04 | HDL |
| rs2923084 | 11 | 10388782 | 1.00 | A | G | 0.03 | 0.00 | 0.02 | 0.03 | 0.04 | 0.06 | -0.04 | 0.06 | 0.04 | 0.04 | HDL |
| rs3136441 | 11 | 46743247 | 1.00 | C | T | 0.05 | 0.00 | 0.00 | 0.04 | -0.09 | 0.07 | 0.02 | 0.08 | 0.04 | 0.05 | HDL |
| rs11246602 | 11 | 51512090 | 1.00 | C | T | 0.03 | 0.01 | -0.06 | 0.04 | -0.04 | 0.07 | -0.11 | 0.08 | -0.04 | 0.05 | HDL |
| rs174546 | 11 | 61569830 | 1.00 | C | T | 0.04 | 0.00 | 0.00 | 0.03 | -0.05 | 0.05 | 0.02 | 0.05 | 0.01 | 0.03 | HDL |
| rs12801636 | 11 | 65391317 | 1.00 | A | G | 0.02 | 0.00 | -0.03 | 0.03 | 0.02 | 0.06 | -0.18 | 0.06 | 0.02 | 0.04 | HDL |
| rs499974 | 11 | 75455021 | 1.00 | C | A | 0.03 | 0.00 | -0.03 | 0.03 | -0.07 | 0.06 | -0.12 | 0.07 | 0.04 | 0.04 | HDL |
| rs3741298 | 11 | 116657561 | 0.98 | T | C | 0.05 | 0.01 | 0.05 | 0.03 | 0.05 | 0.06 | 0.18 | 0.06 | -0.01 | 0.04 | HDL |
| rs7134375 | 12 | 20473758 | 1.00 | A | C | 0.02 | 0.00 | 0.01 | 0.02 | 0.03 | 0.05 | 0.00 | 0.05 | 0.01 | 0.03 | HDL |
| rs11613352 | 12 | 57792580 | 1.00 | T | C | 0.03 | 0.00 | 0.01 | 0.03 | 0.01 | 0.06 | -0.01 | 0.06 | 0.02 | 0.04 | HDL |
| rs7134594 | 12 | 110000193 | 1.00 | T | C | 0.04 | 0.00 | 0.01 | 0.02 | -0.03 | 0.05 | -0.04 | 0.05 | 0.06 | 0.03 | HDL |
| rs4759375 | 12 | 123796238 | 0.95 | T | C | 0.06 | 0.01 | 0.08 | 0.04 | 0.09 | 0.09 | 0.12 | 0.09 | 0.05 | 0.06 | HDL |
| rs4765127 | 12 | 124460167 | 1.00 | T | G | 0.03 | 0.01 | -0.02 | 0.02 | -0.02 | 0.05 | -0.03 | 0.05 | -0.01 | 0.03 | HDL |
| rs838880 | 12 | 125261593 | 0.99 | C | T | 0.05 | 0.00 | 0.04 | 0.03 | 0.07 | 0.05 | 0.10 | 0.05 | -0.01 | 0.04 | HDL |
| rs4983559 | 14 | 105277209 | 1.00 | G | A | 0.02 | 0.00 | 0.02 | 0.02 | -0.03 | 0.05 | 0.04 | 0.05 | 0.03 | 0.03 | HDL |
| rs1532085 | 15 | 58683366 | 1.00 | A | G | 0.11 | 0.00 | 0.01 | 0.02 | 0.06 | 0.05 | 0.01 | 0.05 | -0.02 | 0.03 | HDL |
| rs2652834 | 15 | 63396867 | 1.00 | G | A | 0.03 | 0.00 | 0.04 | 0.03 | -0.01 | 0.06 | 0.06 | 0.06 | 0.06 | 0.04 | HDL |
| rs1121980 | 16 | 53809247 | 1.00 | G | A | 0.02 | 0.00 | -0.06 | 0.02 | -0.10 | 0.05 | -0.02 | 0.05 | -0.06 | 0.03 | HDL |
| rs3764261 | 16 | 56993324 | 0.98 | A | C | 0.24 | 0.00 | 0.01 | 0.03 | 0.06 | 0.05 | 0.04 | 0.05 | -0.02 | 0.04 | HDL |
| rs16942887 | 16 | 67928042 | 1.00 | A | G | 0.08 | 0.01 | 0.00 | 0.04 | 0.10 | 0.07 | -0.05 | 0.08 | -0.01 | 0.05 | HDL |
| rs2925979 | 16 | 81534790 | 1.00 | C | T | 0.04 | 0.00 | 0.06 | 0.03 | 0.07 | 0.05 | 0.03 | 0.05 | 0.07 | 0.04 | HDL |
| rs1877031 | 17 | 37814080 | 1.00 | A | G | 0.03 | 0.00 | -0.08 | 0.03 | -0.02 | 0.05 | -0.04 | 0.05 | -0.13 | 0.03 | HDL |
| rs4148005 | 17 | 66882466 | 1.00 | T | G | 0.03 | 0.00 | -0.03 | 0.03 | 0.03 | 0.05 | -0.01 | 0.05 | -0.08 | 0.04 | HDL |
| rs4129767 | 17 | 76403984 | 1.00 | A | G | 0.02 | 0.00 | 0.01 | 0.02 | 0.08 | 0.05 | -0.01 | 0.05 | -0.01 | 0.03 | HDL |
| rs7241918 | 18 | 47160953 | 1.00 | T | G | 0.09 | 0.01 | -0.04 | 0.03 | -0.01 | 0.06 | -0.03 | 0.07 | -0.05 | 0.04 | HDL |
| rs12967135 | 18 | 57849023 | 1.00 | G | A | 0.03 | 0.00 | -0.04 | 0.03 | -0.05 | 0.06 | -0.01 | 0.06 | -0.04 | 0.04 | HDL |
| rs7255436 | 19 | 8433196 | 1.00 | A | C | 0.03 | 0.01 | 0.02 | 0.02 | 0.03 | 0.05 | -0.02 | 0.05 | 0.03 | 0.03 | HDL |
| rs737337 | 19 | 11347493 | 1.00 | T | C | 0.06 | 0.01 | -0.10 | 0.04 | -0.19 | 0.08 | -0.06 | 0.09 | -0.07 | 0.06 | HDL |
| rs731839 | 19 | 33899065 | 0.99 | A | G | 0.02 | 0.00 | -0.01 | 0.03 | -0.03 | 0.05 | -0.07 | 0.05 | 0.03 | 0.03 | HDL |
| rs4420638 | 19 | 45422946 | 0.81 | A | G | 0.07 | 0.01 | -0.02 | 0.03 | -0.10 | 0.07 | 0.01 | 0.08 | 0.01 | 0.05 | HDL |
| rs17695224 | 19 | 52324216 | 1.00 | G | A | 0.03 | 0.00 | -0.04 | 0.03 | -0.02 | 0.05 | 0.02 | 0.06 | -0.07 | 0.04 | HDL |
| rs103294 | 19 | 54797848 | 1.00 | T | C | 0.05 | 0.00 | -0.02 | 0.03 | -0.13 | 0.06 | 0.07 | 0.06 | 0.00 | 0.04 | HDL |
| rs1800961 | 20 | 43042364 | 0.99 | C | T | 0.13 | 0.01 | 0.14 | 0.07 | 0.18 | 0.14 | 0.11 | 0.15 | 0.14 | 0.10 | HDL |
| rs6065906 | 20 | 44554015 | 1.00 | T | C | 0.06 | 0.00 | 0.03 | 0.03 | -0.01 | 0.06 | -0.01 | 0.07 | 0.07 | 0.04 | HDL |
| rs10903129 | 1 | 25768937 | 1.00 | G | A | 0.03 | 0.00 | -0.01 | 0.02 | -0.03 | 0.05 | 0.05 | 0.05 | -0.02 | 0.03 | LDL |
| rs12748152 | 1 | 27138393 | 0.99 | T | C | 0.05 | 0.01 | -0.05 | 0.04 | 0.01 | 0.09 | -0.04 | 0.10 | -0.07 | 0.06 | LDL |
| rs2479409 | 1 | 55504650 | 0.95 | G | A | 0.06 | 0.00 | 0.00 | 0.03 | -0.01 | 0.05 | 0.03 | 0.05 | 0.00 | 0.03 | LDL |
| rs2131925 | 1 | 63025942 | 1.00 | T | G | 0.05 | 0.00 | -0.03 | 0.03 | -0.04 | 0.05 | -0.05 | 0.05 | -0.01 | 0.03 | LDL |
| rs629301 | 1 | 109818306 | 1.00 | T | G | 0.17 | 0.00 | 0.03 | 0.03 | 0.02 | 0.06 | 0.08 | 0.06 | 0.01 | 0.04 | LDL |
| rs267733 | 1 | 150958836 | 1.00 | A | G | 0.03 | 0.01 | -0.02 | 0.03 | -0.07 | 0.07 | 0.04 | 0.07 | -0.03 | 0.04 | LDL |
| rs2642442 | 1 | 220973563 | 0.96 | T | C | 0.04 | 0.01 | 0.01 | 0.03 | 0.03 | 0.05 | 0.00 | 0.05 | 0.00 | 0.04 | LDL |
| rs484084 | 1 | 234857676 | 0.98 | C | T | 0.03 | 0.01 | -0.05 | 0.02 | -0.03 | 0.05 | -0.04 | 0.05 | -0.06 | 0.03 | LDL |
| rs1367117 | 2 | 21263900 | 0.98 | A | G | 0.12 | 0.00 | 0.00 | 0.03 | 0.07 | 0.05 | -0.01 | 0.05 | -0.03 | 0.04 | LDL |
| rs4299376 | 2 | 44072576 | 1.00 | G | T | 0.08 | 0.00 | -0.02 | 0.03 | -0.03 | 0.05 | -0.02 | 0.05 | -0.01 | 0.04 | LDL |
| rs2710642 | 2 | 63149557 | 1.00 | A | G | 0.02 | 0.00 | -0.04 | 0.03 | -0.07 | 0.05 | 0.02 | 0.05 | -0.05 | 0.03 | LDL |
| rs10490626 | 2 | 118835841 | 1.00 | G | A | 0.05 | 0.01 | 0.03 | 0.04 | 0.05 | 0.09 | -0.05 | 0.09 | 0.06 | 0.06 | LDL |
| rs2030746 | 2 | 121309488 | 1.00 | T | C | 0.02 | 0.00 | 0.02 | 0.02 | -0.08 | 0.05 | 0.07 | 0.05 | 0.05 | 0.03 | LDL |
| rs1250229 | 2 | 216304384 | 0.98 | C | T | 0.02 | 0.00 | -0.03 | 0.03 | -0.12 | 0.06 | 0.09 | 0.06 | -0.04 | 0.04 | LDL |
| rs11563251 | 2 | 234679384 | 0.99 | T | C | 0.03 | 0.01 | -0.03 | 0.04 | -0.05 | 0.08 | 0.15 | 0.08 | -0.11 | 0.05 | LDL |
| rs7640978 | 3 | 32533010 | 0.99 | C | T | 0.04 | 0.01 | 0.02 | 0.04 | 0.05 | 0.08 | -0.14 | 0.09 | 0.08 | 0.06 | LDL |
| rs17404153 | 3 | 132163200 | 1.00 | G | T | 0.03 | 0.01 | -0.02 | 0.04 | -0.02 | 0.07 | -0.02 | 0.08 | -0.03 | 0.05 | LDL |
| rs6831256 | 4 | 3473139 | 1.00 | G | A | 0.02 | 0.00 | 0.02 | 0.02 | 0.05 | 0.05 | -0.03 | 0.05 | 0.03 | 0.03 | LDL |
| rs12916 | 5 | 74656539 | 1.00 | C | T | 0.07 | 0.00 | -0.04 | 0.02 | 0.03 | 0.05 | -0.11 | 0.05 | -0.04 | 0.03 | LDL |
| rs4530754 | 5 | 122855416 | 1.00 | A | G | 0.03 | 0.00 | 0.01 | 0.02 | -0.03 | 0.05 | 0.07 | 0.05 | 0.00 | 0.03 | LDL |
| rs6882076 | 5 | 156390297 | 0.99 | C | T | 0.05 | 0.00 | 0.00 | 0.02 | 0.01 | 0.05 | 0.05 | 0.05 | -0.03 | 0.03 | LDL |
| rs3757354 | 6 | 16127407 | 1.00 | C | T | 0.04 | 0.00 | 0.01 | 0.03 | 0.04 | 0.06 | -0.01 | 0.06 | 0.01 | 0.04 | LDL |
| rs1800562 | 6 | 26093141 | 1.00 | G | A | 0.06 | 0.01 | 0.02 | 0.05 | -0.02 | 0.10 | -0.11 | 0.10 | 0.09 | 0.07 | LDL |
| rs3798236 | 6 | 116309649 | 0.99 | T | C | 0.02 | 0.00 | -0.01 | 0.03 | -0.04 | 0.05 | -0.07 | 0.05 | 0.02 | 0.03 | LDL |
| rs1564348 | 6 | 160578860 | 1.00 | C | T | 0.05 | 0.01 | -0.02 | 0.03 | -0.03 | 0.07 | 0.01 | 0.07 | -0.02 | 0.04 | LDL |
| rs12670798 | 7 | 21607352 | 1.00 | C | T | 0.03 | 0.00 | -0.01 | 0.03 | 0.01 | 0.05 | -0.08 | 0.06 | 0.01 | 0.04 | LDL |
| rs4722551 | 7 | 25991826 | 1.00 | C | T | 0.04 | 0.00 | -0.03 | 0.03 | -0.10 | 0.06 | -0.04 | 0.07 | 0.02 | 0.04 | LDL |
| rs2072183 | 7 | 44581986 | 1.00 | C | T | 0.05 | 0.00 | -0.04 | 0.03 | -0.12 | 0.06 | -0.06 | 0.06 | 0.00 | 0.04 | LDL |
| rs9987289 | 8 | 9183358 | 1.00 | G | A | 0.07 | 0.01 | 0.14 | 0.04 | 0.26 | 0.08 | -0.02 | 0.09 | 0.15 | 0.06 | LDL |
| rs10102164 | 8 | 55421614 | 0.99 | A | G | 0.03 | 0.00 | 0.02 | 0.03 | 0.02 | 0.06 | 0.07 | 0.06 | 0.00 | 0.04 | LDL |
| rs2081687 | 8 | 59388565 | 1.00 | T | C | 0.03 | 0.01 | -0.03 | 0.02 | 0.02 | 0.05 | -0.04 | 0.05 | -0.04 | 0.03 | LDL |
| rs10808546 | 8 | 126495818 | 1.00 | C | T | 0.05 | 0.00 | -0.01 | 0.02 | -0.04 | 0.05 | 0.04 | 0.05 | -0.02 | 0.03 | LDL |
| rs11136341 | 8 | 145043543 | 0.96 | G | A | 0.04 | 0.01 | -0.01 | 0.02 | -0.05 | 0.05 | 0.00 | 0.05 | 0.00 | 0.03 | LDL |
| rs3780181 | 9 | 2640759 | 1.00 | A | G | 0.04 | 0.01 | 0.03 | 0.05 | -0.02 | 0.09 | 0.10 | 0.10 | 0.02 | 0.07 | LDL |
| rs2255141 | 10 | 113933886 | 1.00 | A | G | 0.03 | 0.00 | 0.00 | 0.03 | 0.00 | 0.05 | 0.02 | 0.05 | 0.00 | 0.04 | LDL |
| rs11220462 | 11 | 126243952 | 1.00 | A | G | 0.06 | 0.01 | -0.01 | 0.03 | -0.01 | 0.07 | -0.02 | 0.07 | 0.00 | 0.05 | LDL |
| rs11065987 | 12 | 112072424 | 1.00 | A | G | 0.03 | 0.00 | 0.03 | 0.02 | 0.03 | 0.05 | 0.12 | 0.05 | 0.00 | 0.03 | LDL |
| rs1169288 | 12 | 121416650 | 0.98 | C | A | 0.04 | 0.00 | 0.12 | 0.03 | 0.19 | 0.05 | 0.13 | 0.05 | 0.07 | 0.03 | LDL |
| rs4942486 | 13 | 32953388 | 1.00 | T | C | 0.02 | 0.00 | -0.03 | 0.02 | 0.00 | 0.05 | -0.01 | 0.05 | -0.05 | 0.03 | LDL |
| rs8017377 | 14 | 24883887 | 1.00 | A | G | 0.03 | 0.00 | 0.03 | 0.02 | 0.07 | 0.05 | -0.08 | 0.05 | 0.05 | 0.03 | LDL |
| rs3764261 | 16 | 56993324 | 0.98 | C | A | 0.05 | 0.00 | -0.01 | 0.03 | -0.06 | 0.05 | -0.04 | 0.05 | 0.02 | 0.04 | LDL |
| rs2000999 | 16 | 72108093 | 1.00 | A | G | 0.07 | 0.00 | 0.01 | 0.03 | 0.05 | 0.06 | -0.03 | 0.06 | 0.01 | 0.04 | LDL |
| rs314253 | 17 | 7091650 | 1.00 | T | C | 0.02 | 0.00 | 0.04 | 0.03 | 0.03 | 0.05 | 0.08 | 0.05 | 0.03 | 0.04 | LDL |
| rs7206971 | 17 | 45425115 | 1.00 | A | G | 0.03 | 0.01 | 0.04 | 0.02 | 0.06 | 0.05 | 0.09 | 0.05 | 0.00 | 0.03 | LDL |
| rs1801689 | 17 | 64210580 | 0.72 | C | A | 0.10 | 0.01 | -0.14 | 0.07 | -0.33 | 0.18 | -0.12 | 0.17 | -0.10 | 0.09 | LDL |
| rs6511720 | 19 | 11202306 | 1.00 | G | T | 0.22 | 0.01 | -0.06 | 0.04 | -0.10 | 0.08 | 0.04 | 0.08 | -0.08 | 0.05 | LDL |
| rs10401969 | 19 | 19407718 | 0.99 | T | C | 0.12 | 0.01 | -0.06 | 0.04 | -0.07 | 0.09 | -0.09 | 0.09 | -0.04 | 0.06 | LDL |
| rs4420638 | 19 | 45422946 | 0.81 | G | A | 0.23 | 0.01 | 0.02 | 0.03 | 0.10 | 0.07 | -0.01 | 0.08 | -0.01 | 0.05 | LDL |
| rs364585 | 20 | 12962718 | 1.00 | G | A | 0.02 | 0.00 | 0.00 | 0.02 | -0.03 | 0.05 | 0.07 | 0.05 | -0.01 | 0.03 | LDL |
| rs2328223 | 20 | 17845921 | 1.00 | C | A | 0.03 | 0.01 | -0.05 | 0.03 | -0.16 | 0.06 | -0.05 | 0.06 | 0.00 | 0.04 | LDL |
| rs2902940 | 20 | 39091487 | 1.00 | A | G | 0.03 | 0.00 | 0.04 | 0.03 | -0.03 | 0.05 | 0.04 | 0.05 | 0.07 | 0.04 | LDL |
| rs6016505 | 20 | 39678289 | 0.98 | T | C | 0.04 | 0.01 | 0.02 | 0.02 | 0.03 | 0.05 | -0.08 | 0.05 | 0.06 | 0.03 | LDL |
| rs5763662 | 22 | 30378703 | 1.00 | T | C | 0.08 | 0.01 | 0.10 | 0.08 | 0.29 | 0.16 | 0.15 | 0.17 | -0.01 | 0.11 | LDL |
| rs4253772 | 22 | 46627603 | 1.00 | T | C | 0.03 | 0.01 | -0.07 | 0.04 | -0.06 | 0.08 | -0.06 | 0.08 | -0.08 | 0.05 | LDL |
| rs1077514 | 1 | 23766233 | 0.99 | T | C | 0.03 | 0.01 | 0.06 | 0.03 | 0.10 | 0.07 | 0.06 | 0.07 | 0.05 | 0.05 | Total cholesterol |
| rs10903129 | 1 | 25768937 | 1.00 | G | A | 0.03 | 0.00 | -0.01 | 0.02 | -0.03 | 0.05 | 0.05 | 0.05 | -0.02 | 0.03 | Total cholesterol |
| rs2479409 | 1 | 55504650 | 0.95 | G | A | 0.05 | 0.00 | 0.00 | 0.03 | -0.01 | 0.05 | 0.03 | 0.05 | 0.00 | 0.03 | Total cholesterol |
| rs2131925 | 1 | 63025942 | 1.00 | T | G | 0.07 | 0.00 | -0.03 | 0.03 | -0.04 | 0.05 | -0.05 | 0.05 | -0.01 | 0.03 | Total cholesterol |
| rs7515577 | 1 | 93009438 | 1.00 | A | C | 0.04 | 0.01 | 0.07 | 0.03 | 0.02 | 0.06 | 0.06 | 0.06 | 0.09 | 0.04 | Total cholesterol |
| rs629301 | 1 | 109818306 | 1.00 | T | G | 0.13 | 0.00 | 0.03 | 0.03 | 0.02 | 0.06 | 0.08 | 0.06 | 0.01 | 0.04 | Total cholesterol |
| rs2642442 | 1 | 220973563 | 0.96 | T | C | 0.04 | 0.01 | 0.01 | 0.03 | 0.03 | 0.05 | 0.00 | 0.05 | 0.00 | 0.04 | Total cholesterol |
| rs484084 | 1 | 234857676 | 0.98 | C | T | 0.03 | 0.01 | -0.05 | 0.02 | -0.03 | 0.05 | -0.04 | 0.05 | -0.06 | 0.03 | Total cholesterol |
| rs1367117 | 2 | 21263900 | 0.98 | A | G | 0.10 | 0.00 | 0.00 | 0.03 | 0.07 | 0.05 | -0.01 | 0.05 | -0.03 | 0.04 | Total cholesterol |
| rs1260326 | 2 | 27730940 | 1.00 | T | C | 0.05 | 0.00 | -0.02 | 0.02 | -0.01 | 0.05 | -0.08 | 0.05 | 0.01 | 0.03 | Total cholesterol |
| rs4299376 | 2 | 44072576 | 1.00 | G | T | 0.08 | 0.00 | -0.02 | 0.03 | -0.03 | 0.05 | -0.02 | 0.05 | -0.01 | 0.04 | Total cholesterol |
| rs10490626 | 2 | 118835841 | 1.00 | G | A | 0.04 | 0.01 | 0.03 | 0.04 | 0.05 | 0.09 | -0.05 | 0.09 | 0.06 | 0.06 | Total cholesterol |
| rs2030746 | 2 | 121309488 | 1.00 | T | C | 0.02 | 0.00 | 0.02 | 0.02 | -0.08 | 0.05 | 0.07 | 0.05 | 0.05 | 0.03 | Total cholesterol |
| rs7570971 | 2 | 135837906 | 0.92 | A | C | 0.03 | 0.00 | 0.01 | 0.03 | -0.07 | 0.05 | -0.03 | 0.06 | 0.08 | 0.04 | Total cholesterol |
| rs2287623 | 2 | 169830155 | 1.00 | G | A | 0.03 | 0.00 | -0.01 | 0.02 | 0.02 | 0.05 | -0.03 | 0.05 | -0.01 | 0.03 | Total cholesterol |
| rs11694172 | 2 | 203532304 | 1.00 | G | A | 0.03 | 0.00 | 0.01 | 0.03 | 0.00 | 0.06 | -0.03 | 0.06 | 0.03 | 0.04 | Total cholesterol |
| rs11563251 | 2 | 234679384 | 0.99 | T | C | 0.04 | 0.01 | -0.03 | 0.04 | -0.05 | 0.08 | 0.15 | 0.08 | -0.11 | 0.05 | Total cholesterol |
| rs7956 | 3 | 12624763 | 1.00 | T | C | 0.03 | 0.01 | -0.03 | 0.03 | -0.12 | 0.06 | 0.00 | 0.06 | 0.00 | 0.04 | Total cholesterol |
| rs7640978 | 3 | 32533010 | 0.99 | C | T | 0.04 | 0.01 | 0.02 | 0.04 | 0.05 | 0.08 | -0.14 | 0.09 | 0.08 | 0.06 | Total cholesterol |
| rs13315871 | 3 | 58381287 | 1.00 | G | A | 0.04 | 0.01 | 0.05 | 0.04 | -0.08 | 0.08 | 0.14 | 0.08 | 0.07 | 0.06 | Total cholesterol |
| rs6831256 | 4 | 3473139 | 1.00 | G | A | 0.02 | 0.00 | 0.02 | 0.02 | 0.05 | 0.05 | -0.03 | 0.05 | 0.03 | 0.03 | Total cholesterol |
| rs12916 | 5 | 74656539 | 1.00 | C | T | 0.07 | 0.00 | -0.04 | 0.02 | 0.03 | 0.05 | -0.11 | 0.05 | -0.04 | 0.03 | Total cholesterol |
| rs4530754 | 5 | 122855416 | 1.00 | A | G | 0.02 | 0.00 | 0.01 | 0.02 | -0.03 | 0.05 | 0.07 | 0.05 | 0.00 | 0.03 | Total cholesterol |
| rs6882076 | 5 | 156390297 | 0.99 | C | T | 0.05 | 0.00 | 0.00 | 0.02 | 0.01 | 0.05 | 0.05 | 0.05 | -0.03 | 0.03 | Total cholesterol |
| rs3757354 | 6 | 16127407 | 1.00 | C | T | 0.03 | 0.00 | 0.01 | 0.03 | 0.04 | 0.06 | -0.01 | 0.06 | 0.01 | 0.04 | Total cholesterol |
| rs1800562 | 6 | 26093141 | 1.00 | G | A | 0.06 | 0.01 | 0.02 | 0.05 | -0.02 | 0.10 | -0.11 | 0.10 | 0.09 | 0.07 | Total cholesterol |
| rs2814982 | 6 | 34546560 | 0.99 | C | T | 0.04 | 0.01 | 0.02 | 0.04 | -0.01 | 0.07 | -0.03 | 0.08 | 0.07 | 0.05 | Total cholesterol |
| rs2758886 | 6 | 39250837 | 1.00 | A | G | 0.02 | 0.00 | 0.01 | 0.03 | 0.02 | 0.05 | 0.10 | 0.05 | -0.03 | 0.04 | Total cholesterol |
| rs3798236 | 6 | 116309649 | 0.99 | T | C | 0.03 | 0.00 | -0.01 | 0.03 | -0.04 | 0.05 | -0.07 | 0.05 | 0.02 | 0.03 | Total cholesterol |
| rs9376090 | 6 | 135411228 | 0.99 | T | C | 0.03 | 0.00 | -0.04 | 0.03 | -0.01 | 0.05 | -0.01 | 0.06 | -0.06 | 0.04 | Total cholesterol |
| rs1564348 | 6 | 160578860 | 1.00 | C | T | 0.05 | 0.00 | -0.02 | 0.03 | -0.03 | 0.07 | 0.01 | 0.07 | -0.02 | 0.04 | Total cholesterol |
| rs1997243 | 7 | 1083777 | 1.00 | G | A | 0.03 | 0.01 | -0.05 | 0.03 | -0.05 | 0.07 | -0.03 | 0.07 | -0.05 | 0.04 | Total cholesterol |
| rs12670798 | 7 | 21607352 | 1.00 | C | T | 0.04 | 0.00 | -0.01 | 0.03 | 0.01 | 0.05 | -0.08 | 0.06 | 0.01 | 0.04 | Total cholesterol |
| rs4722551 | 7 | 25991826 | 1.00 | C | T | 0.03 | 0.00 | -0.03 | 0.03 | -0.10 | 0.06 | -0.04 | 0.07 | 0.02 | 0.04 | Total cholesterol |
| rs2072183 | 7 | 44581986 | 1.00 | C | T | 0.04 | 0.00 | -0.04 | 0.03 | -0.12 | 0.06 | -0.06 | 0.06 | 0.00 | 0.04 | Total cholesterol |
| rs9987289 | 8 | 9183358 | 1.00 | G | A | 0.08 | 0.01 | 0.14 | 0.04 | 0.26 | 0.08 | -0.02 | 0.09 | 0.15 | 0.06 | Total cholesterol |
| rs1495741 | 8 | 18272881 | 1.00 | G | A | 0.03 | 0.01 | 0.06 | 0.03 | 0.11 | 0.05 | 0.11 | 0.06 | 0.01 | 0.04 | Total cholesterol |
| rs10102164 | 8 | 55421614 | 0.99 | A | G | 0.03 | 0.00 | 0.02 | 0.03 | 0.02 | 0.06 | 0.07 | 0.06 | 0.00 | 0.04 | Total cholesterol |
| rs2081687 | 8 | 59388565 | 1.00 | T | C | 0.04 | 0.01 | -0.03 | 0.02 | 0.02 | 0.05 | -0.04 | 0.05 | -0.04 | 0.03 | Total cholesterol |
| rs2737229 | 8 | 116648565 | 1.00 | A | C | 0.03 | 0.00 | -0.03 | 0.03 | -0.03 | 0.05 | 0.00 | 0.05 | -0.04 | 0.04 | Total cholesterol |
| rs10808546 | 8 | 126495818 | 1.00 | C | T | 0.06 | 0.00 | -0.01 | 0.02 | -0.04 | 0.05 | 0.04 | 0.05 | -0.02 | 0.03 | Total cholesterol |
| rs11136341 | 8 | 145043543 | 0.96 | G | A | 0.04 | 0.01 | -0.01 | 0.02 | -0.05 | 0.05 | 0.00 | 0.05 | 0.00 | 0.03 | Total cholesterol |
| rs3780181 | 9 | 2640759 | 1.00 | A | G | 0.04 | 0.01 | 0.03 | 0.05 | -0.02 | 0.09 | 0.10 | 0.10 | 0.02 | 0.07 | Total cholesterol |
| rs638491 | 9 | 15290012 | 1.00 | G | A | 0.03 | 0.00 | 0.00 | 0.03 | -0.03 | 0.06 | 0.01 | 0.07 | 0.00 | 0.04 | Total cholesterol |
| rs1883025 | 9 | 107664301 | 0.99 | C | T | 0.07 | 0.00 | 0.02 | 0.03 | 0.02 | 0.06 | 0.03 | 0.06 | 0.02 | 0.04 | Total cholesterol |
| rs10904908 | 10 | 17260290 | 0.99 | G | A | 0.03 | 0.00 | -0.03 | 0.02 | -0.06 | 0.05 | 0.01 | 0.05 | -0.04 | 0.03 | Total cholesterol |
| rs970548 | 10 | 46013277 | 1.00 | C | A | 0.03 | 0.00 | -0.07 | 0.03 | -0.15 | 0.05 | -0.09 | 0.06 | -0.02 | 0.04 | Total cholesterol |
| rs2255141 | 10 | 113933886 | 1.00 | A | G | 0.03 | 0.00 | 0.00 | 0.03 | 0.00 | 0.05 | 0.02 | 0.05 | 0.00 | 0.04 | Total cholesterol |
| rs10128711 | 11 | 18632984 | 0.98 | C | T | 0.03 | 0.00 | 0.05 | 0.03 | 0.06 | 0.05 | 0.00 | 0.06 | 0.06 | 0.04 | Total cholesterol |
| rs174546 | 11 | 61569830 | 1.00 | C | T | 0.05 | 0.00 | 0.00 | 0.03 | -0.05 | 0.05 | 0.02 | 0.05 | 0.01 | 0.03 | Total cholesterol |
| rs3741298 | 11 | 116657561 | 0.98 | C | T | 0.07 | 0.01 | -0.05 | 0.03 | -0.05 | 0.06 | -0.18 | 0.06 | 0.01 | 0.04 | Total cholesterol |
| rs11603023 | 11 | 118486067 | 1.00 | T | C | 0.02 | 0.00 | -0.02 | 0.02 | -0.01 | 0.05 | 0.01 | 0.05 | -0.04 | 0.03 | Total cholesterol |
| rs11220462 | 11 | 126243952 | 1.00 | A | G | 0.05 | 0.01 | -0.01 | 0.03 | -0.01 | 0.07 | -0.02 | 0.07 | 0.00 | 0.05 | Total cholesterol |
| rs4883201 | 12 | 9082581 | 1.00 | A | G | 0.04 | 0.01 | 0.06 | 0.04 | -0.07 | 0.08 | 0.26 | 0.08 | 0.03 | 0.05 | Total cholesterol |
| rs11065987 | 12 | 112072424 | 1.00 | A | G | 0.03 | 0.00 | 0.03 | 0.02 | 0.03 | 0.05 | 0.12 | 0.05 | 0.00 | 0.03 | Total cholesterol |
| rs1169288 | 12 | 121416650 | 0.98 | C | A | 0.03 | 0.00 | 0.12 | 0.03 | 0.19 | 0.05 | 0.13 | 0.05 | 0.07 | 0.03 | Total cholesterol |
| rs1532085 | 15 | 58683366 | 1.00 | A | G | 0.05 | 0.00 | 0.01 | 0.02 | 0.06 | 0.05 | 0.01 | 0.05 | -0.02 | 0.03 | Total cholesterol |
| rs3764261 | 16 | 56993324 | 0.98 | A | C | 0.05 | 0.00 | 0.01 | 0.03 | 0.06 | 0.05 | 0.04 | 0.05 | -0.02 | 0.04 | Total cholesterol |
| rs2000999 | 16 | 72108093 | 1.00 | A | G | 0.06 | 0.00 | 0.01 | 0.03 | 0.05 | 0.06 | -0.03 | 0.06 | 0.01 | 0.04 | Total cholesterol |
| rs314253 | 17 | 7091650 | 1.00 | T | C | 0.02 | 0.00 | 0.04 | 0.03 | 0.03 | 0.05 | 0.08 | 0.05 | 0.03 | 0.04 | Total cholesterol |
| rs7206971 | 17 | 45425115 | 1.00 | A | G | 0.03 | 0.01 | 0.04 | 0.02 | 0.06 | 0.05 | 0.09 | 0.05 | 0.00 | 0.03 | Total cholesterol |
| rs7241918 | 18 | 47160953 | 1.00 | T | G | 0.06 | 0.01 | -0.04 | 0.03 | -0.01 | 0.06 | -0.03 | 0.07 | -0.05 | 0.04 | Total cholesterol |
| rs6511720 | 19 | 11202306 | 1.00 | G | T | 0.19 | 0.01 | -0.06 | 0.04 | -0.10 | 0.08 | 0.04 | 0.08 | -0.08 | 0.05 | Total cholesterol |
| rs10401969 | 19 | 19407718 | 0.99 | T | C | 0.14 | 0.01 | -0.06 | 0.04 | -0.07 | 0.09 | -0.09 | 0.09 | -0.04 | 0.06 | Total cholesterol |
| rs4420638 | 19 | 45422946 | 0.81 | G | A | 0.20 | 0.01 | 0.02 | 0.03 | 0.10 | 0.07 | -0.01 | 0.08 | -0.01 | 0.05 | Total cholesterol |
| rs492602 | 19 | 49206417 | 1.00 | G | A | 0.03 | 0.00 | 0.01 | 0.02 | -0.05 | 0.05 | 0.04 | 0.05 | 0.02 | 0.03 | Total cholesterol |
| rs2277862 | 20 | 34152782 | 1.00 | C | T | 0.03 | 0.01 | -0.03 | 0.03 | 0.02 | 0.07 | -0.05 | 0.07 | -0.04 | 0.05 | Total cholesterol |
| rs2902940 | 20 | 39091487 | 1.00 | A | G | 0.02 | 0.00 | 0.04 | 0.03 | -0.03 | 0.05 | 0.04 | 0.05 | 0.07 | 0.04 | Total cholesterol |
| rs6016505 | 20 | 39678289 | 0.98 | T | C | 0.04 | 0.01 | 0.02 | 0.02 | 0.03 | 0.05 | -0.08 | 0.05 | 0.06 | 0.03 | Total cholesterol |
| rs1800961 | 20 | 43042364 | 0.99 | C | T | 0.11 | 0.01 | 0.14 | 0.07 | 0.18 | 0.14 | 0.11 | 0.15 | 0.14 | 0.10 | Total cholesterol |
| rs138777 | 22 | 35711098 | 1.00 | A | G | 0.02 | 0.00 | -0.01 | 0.02 | -0.02 | 0.05 | -0.01 | 0.05 | 0.00 | 0.03 | Total cholesterol |
| rs4253772 | 22 | 46627603 | 1.00 | T | C | 0.03 | 0.01 | -0.07 | 0.04 | -0.06 | 0.08 | -0.06 | 0.08 | -0.08 | 0.05 | Total cholesterol |
| rs12748152 | 1 | 27138393 | 0.99 | T | C | 0.04 | 0.01 | -0.05 | 0.04 | 0.01 | 0.09 | -0.04 | 0.10 | -0.07 | 0.06 | Triglycerides |
| rs2131925 | 1 | 63025942 | 1.00 | T | G | 0.07 | 0.00 | -0.03 | 0.03 | -0.04 | 0.05 | -0.05 | 0.05 | -0.01 | 0.03 | Triglycerides |
| rs4846914 | 1 | 230295691 | 0.98 | G | A | 0.04 | 0.00 | -0.03 | 0.02 | 0.04 | 0.05 | 0.02 | 0.05 | -0.09 | 0.03 | Triglycerides |
| rs1042034 | 2 | 21225281 | 1.00 | T | C | 0.07 | 0.00 | -0.04 | 0.03 | 0.01 | 0.06 | -0.07 | 0.06 | -0.06 | 0.04 | Triglycerides |
| rs1260326 | 2 | 27730940 | 1.00 | T | C | 0.11 | 0.00 | -0.02 | 0.02 | -0.01 | 0.05 | -0.08 | 0.05 | 0.01 | 0.03 | Triglycerides |
| rs10195252 | 2 | 165513091 | 1.00 | T | C | 0.03 | 0.00 | 0.02 | 0.02 | 0.05 | 0.05 | 0.07 | 0.05 | -0.02 | 0.03 | Triglycerides |
| rs2972146 | 2 | 227100698 | 1.00 | T | G | 0.03 | 0.00 | -0.01 | 0.02 | -0.03 | 0.05 | 0.03 | 0.05 | -0.01 | 0.03 | Triglycerides |
| rs645040 | 3 | 135926622 | 1.00 | T | G | 0.03 | 0.00 | -0.03 | 0.03 | 0.02 | 0.06 | -0.03 | 0.06 | -0.04 | 0.04 | Triglycerides |
| rs6831256 | 4 | 3473139 | 1.00 | G | A | 0.03 | 0.00 | 0.02 | 0.02 | 0.05 | 0.05 | -0.03 | 0.05 | 0.03 | 0.03 | Triglycerides |
| rs442177 | 4 | 88030261 | 1.00 | T | G | 0.03 | 0.00 | 0.00 | 0.02 | -0.02 | 0.05 | 0.03 | 0.05 | 0.00 | 0.03 | Triglycerides |
| rs9686661 | 5 | 55861786 | 0.98 | T | C | 0.04 | 0.00 | 0.06 | 0.03 | 0.13 | 0.06 | 0.13 | 0.06 | 0.01 | 0.04 | Triglycerides |
| rs6882076 | 5 | 156390297 | 0.99 | C | T | 0.03 | 0.00 | 0.00 | 0.02 | 0.01 | 0.05 | 0.05 | 0.05 | -0.03 | 0.03 | Triglycerides |
| rs998584 | 6 | 43757896 | 0.92 | A | C | 0.03 | 0.00 | 0.03 | 0.02 | 0.04 | 0.05 | 0.05 | 0.05 | 0.03 | 0.03 | Triglycerides |
| rs1936800 | 6 | 127436064 | 0.99 | T | C | 0.02 | 0.00 | 0.05 | 0.02 | 0.05 | 0.05 | 0.09 | 0.05 | 0.03 | 0.03 | Triglycerides |
| rs4722551 | 7 | 25991826 | 1.00 | T | C | 0.03 | 0.00 | 0.03 | 0.03 | 0.10 | 0.06 | 0.04 | 0.07 | -0.02 | 0.04 | Triglycerides |
| rs17145738 | 7 | 72982874 | 0.98 | C | T | 0.11 | 0.01 | -0.06 | 0.04 | -0.08 | 0.07 | 0.01 | 0.08 | -0.09 | 0.05 | Triglycerides |
| rs38855 | 7 | 116358044 | 1.00 | A | G | 0.02 | 0.00 | 0.01 | 0.02 | -0.05 | 0.05 | 0.07 | 0.05 | 0.01 | 0.03 | Triglycerides |
| rs2271357 | 8 | 10683623 | 1.00 | A | G | 0.02 | 0.00 | 0.01 | 0.02 | -0.01 | 0.05 | 0.01 | 0.05 | 0.03 | 0.03 | Triglycerides |
| rs1495741 | 8 | 18272881 | 1.00 | G | A | 0.04 | 0.01 | 0.06 | 0.03 | 0.11 | 0.05 | 0.11 | 0.06 | 0.01 | 0.04 | Triglycerides |
| rs12678919 | 8 | 19844222 | 1.00 | A | G | 0.17 | 0.01 | 0.00 | 0.04 | -0.02 | 0.08 | -0.07 | 0.08 | 0.04 | 0.05 | Triglycerides |
| rs10808546 | 8 | 126495818 | 1.00 | C | T | 0.08 | 0.00 | -0.01 | 0.02 | -0.04 | 0.05 | 0.04 | 0.05 | -0.02 | 0.03 | Triglycerides |
| rs1832007 | 10 | 5254847 | 1.00 | A | G | 0.03 | 0.00 | -0.03 | 0.03 | -0.05 | 0.07 | -0.06 | 0.07 | 0.00 | 0.05 | Triglycerides |
| rs7080386 | 10 | 65048306 | 0.99 | C | A | 0.03 | 0.00 | 0.04 | 0.02 | 0.03 | 0.05 | 0.07 | 0.05 | 0.03 | 0.03 | Triglycerides |
| rs2068888 | 10 | 94839642 | 1.00 | G | A | 0.02 | 0.00 | -0.03 | 0.02 | -0.02 | 0.05 | 0.01 | 0.05 | -0.06 | 0.03 | Triglycerides |
| rs174546 | 11 | 61569830 | 1.00 | T | C | 0.04 | 0.00 | 0.00 | 0.03 | 0.05 | 0.05 | -0.02 | 0.05 | -0.01 | 0.03 | Triglycerides |
| rs3741298 | 11 | 116657561 | 0.98 | C | T | 0.15 | 0.01 | -0.05 | 0.03 | -0.05 | 0.06 | -0.18 | 0.06 | 0.01 | 0.04 | Triglycerides |
| rs11613352 | 12 | 57792580 | 1.00 | C | T | 0.03 | 0.00 | -0.01 | 0.03 | -0.01 | 0.06 | 0.01 | 0.06 | -0.02 | 0.04 | Triglycerides |
| rs4765127 | 12 | 124460167 | 1.00 | G | T | 0.03 | 0.00 | 0.02 | 0.02 | 0.02 | 0.05 | 0.03 | 0.05 | 0.01 | 0.03 | Triglycerides |
| rs2412710 | 15 | 42683787 | 0.95 | A | G | 0.10 | 0.01 | 0.14 | 0.09 | 0.36 | 0.19 | -0.03 | 0.19 | 0.11 | 0.11 | Triglycerides |
| rs1532085 | 15 | 58683366 | 1.00 | A | G | 0.03 | 0.00 | 0.01 | 0.02 | 0.06 | 0.05 | 0.01 | 0.05 | -0.02 | 0.03 | Triglycerides |
| rs3198697 | 16 | 15129940 | 1.00 | C | T | 0.02 | 0.00 | -0.01 | 0.02 | 0.05 | 0.05 | -0.02 | 0.05 | -0.03 | 0.03 | Triglycerides |
| rs1121980 | 16 | 53809247 | 1.00 | A | G | 0.02 | 0.00 | 0.06 | 0.02 | 0.10 | 0.05 | 0.02 | 0.05 | 0.06 | 0.03 | Triglycerides |
| rs8077889 | 17 | 41878166 | 0.99 | C | A | 0.03 | 0.00 | -0.03 | 0.03 | -0.05 | 0.06 | -0.11 | 0.06 | 0.01 | 0.04 | Triglycerides |
| rs7248104 | 19 | 7224431 | 1.00 | G | A | 0.02 | 0.00 | 0.05 | 0.02 | 0.08 | 0.05 | 0.04 | 0.05 | 0.03 | 0.03 | Triglycerides |
| rs10401969 | 19 | 19407718 | 0.99 | T | C | 0.12 | 0.01 | -0.06 | 0.04 | -0.07 | 0.09 | -0.09 | 0.09 | -0.04 | 0.06 | Triglycerides |
| rs731839 | 19 | 33899065 | 0.99 | G | A | 0.02 | 0.00 | 0.01 | 0.03 | 0.03 | 0.05 | 0.07 | 0.05 | -0.03 | 0.03 | Triglycerides |
| rs439401 | 19 | 45414451 | 1.00 | C | T | 0.07 | 0.00 | -0.01 | 0.02 | 0.08 | 0.05 | 0.00 | 0.05 | -0.05 | 0.03 | Triglycerides |
| rs6065906 | 20 | 44554015 | 1.00 | C | T | 0.05 | 0.00 | -0.03 | 0.03 | 0.01 | 0.06 | 0.01 | 0.07 | -0.07 | 0.04 | Triglycerides |
| rs5756931 | 22 | 38546033 | 0.98 | T | C | 0.02 | 0.00 | 0.05 | 0.02 | 0.10 | 0.05 | -0.01 | 0.05 | 0.05 | 0.03 | Triglycerides |
| rs340874 | 1 | 214159256 | 1.00 | C | T | 0.02 | 0.00 | 0.04 | 0.02 | 0.01 | 0.05 | 0.03 | 0.05 | 0.06 | 0.03 | Fasting glucose |
| rs780094 | 2 | 27741237 | 1.00 | C | T | 0.04 | 0.00 | 0.01 | 0.02 | 0.00 | 0.05 | 0.08 | 0.05 | -0.02 | 0.03 | Fasting glucose |
| rs560887 | 2 | 169763148 | 1.00 | C | T | 0.09 | 0.00 | -0.03 | 0.03 | -0.07 | 0.05 | -0.09 | 0.06 | 0.00 | 0.04 | Fasting glucose |
| rs11715915 | 3 | 49455330 | 1.00 | C | T | 0.02 | 0.00 | 0.08 | 0.03 | 0.03 | 0.05 | 0.05 | 0.05 | 0.11 | 0.04 | Fasting glucose |
| rs11708067 | 3 | 123065778 | 0.99 | A | G | 0.03 | 0.00 | 0.00 | 0.03 | 0.00 | 0.06 | 0.09 | 0.06 | -0.03 | 0.04 | Fasting glucose |
| rs1280 | 3 | 170713290 | 1.00 | T | C | 0.03 | 0.00 | -0.01 | 0.03 | 0.00 | 0.07 | -0.05 | 0.07 | 0.00 | 0.05 | Fasting glucose |
| rs7651090 | 3 | 185513392 | 1.00 | G | A | 0.02 | 0.00 | 0.00 | 0.03 | 0.05 | 0.05 | -0.05 | 0.05 | -0.01 | 0.04 | Fasting glucose |
| rs7708285 | 5 | 76425867 | 1.00 | G | A | 0.02 | 0.00 | 0.01 | 0.03 | 0.07 | 0.05 | -0.01 | 0.05 | -0.01 | 0.04 | Fasting glucose |
| rs4869272 | 5 | 95539448 | 1.00 | T | C | 0.02 | 0.00 | 0.02 | 0.03 | 0.03 | 0.05 | 0.02 | 0.05 | 0.01 | 0.04 | Fasting glucose |
| rs17762454 | 6 | 7213200 | 1.00 | T | C | 0.02 | 0.00 | 0.03 | 0.03 | 0.00 | 0.05 | 0.00 | 0.06 | 0.06 | 0.04 | Fasting glucose |
| rs9368222 | 6 | 20686996 | 1.00 | A | C | 0.02 | 0.00 | -0.01 | 0.03 | -0.02 | 0.05 | -0.06 | 0.06 | 0.02 | 0.04 | Fasting glucose |
| rs2191349 | 7 | 15064309 | 1.00 | T | G | 0.04 | 0.00 | 0.01 | 0.02 | -0.06 | 0.05 | 0.06 | 0.05 | 0.02 | 0.03 | Fasting glucose |
| rs2908289 | 7 | 44223942 | 1.00 | A | G | 0.08 | 0.00 | 0.04 | 0.03 | 0.07 | 0.06 | 0.07 | 0.06 | 0.01 | 0.04 | Fasting glucose |
| rs6943153 | 7 | 50791579 | 1.00 | T | C | 0.02 | 0.00 | 0.04 | 0.03 | -0.01 | 0.05 | 0.01 | 0.06 | 0.07 | 0.04 | Fasting glucose |
| rs983309 | 8 | 9177732 | 1.00 | T | G | 0.03 | 0.00 | -0.13 | 0.04 | -0.22 | 0.08 | 0.00 | 0.08 | -0.14 | 0.05 | Fasting glucose |
| rs11558471 | 8 | 118185733 | 1.00 | A | G | 0.04 | 0.00 | 0.01 | 0.03 | 0.06 | 0.05 | -0.05 | 0.05 | 0.01 | 0.04 | Fasting glucose |
| rs10814916 | 9 | 4293150 | 1.00 | C | A | 0.02 | 0.00 | -0.02 | 0.02 | 0.01 | 0.05 | 0.00 | 0.05 | -0.04 | 0.03 | Fasting glucose |
| rs10811661 | 9 | 22134094 | 0.97 | T | C | 0.03 | 0.00 | 0.00 | 0.03 | -0.05 | 0.06 | 0.10 | 0.07 | -0.02 | 0.04 | Fasting glucose |
| rs16913693 | 9 | 111680359 | 0.99 | T | G | 0.06 | 0.01 | 0.11 | 0.07 | -0.05 | 0.13 | -0.01 | 0.15 | 0.27 | 0.10 | Fasting glucose |
| rs3829109 | 9 | 139256766 | 0.92 | G | A | 0.02 | 0.00 | -0.03 | 0.03 | -0.07 | 0.06 | -0.02 | 0.06 | -0.01 | 0.04 | Fasting glucose |
| rs11195502 | 10 | 113039667 | 1.00 | C | T | 0.04 | 0.00 | -0.02 | 0.04 | -0.02 | 0.08 | -0.03 | 0.09 | -0.01 | 0.06 | Fasting glucose |
| rs10885122 | 10 | 113042093 | 1.00 | G | T | 0.04 | 0.00 | -0.04 | 0.04 | -0.04 | 0.07 | -0.02 | 0.08 | -0.05 | 0.05 | Fasting glucose |
| rs7901695 | 10 | 114754088 | 1.00 | C | T | 0.03 | 0.00 | 0.01 | 0.03 | -0.03 | 0.05 | 0.01 | 0.05 | 0.03 | 0.04 | Fasting glucose |
| rs11605924 | 11 | 45873091 | 0.99 | A | C | 0.03 | 0.00 | -0.02 | 0.02 | 0.08 | 0.05 | -0.14 | 0.05 | -0.02 | 0.03 | Fasting glucose |
| rs11039182 | 11 | 47346723 | 0.99 | T | C | 0.03 | 0.00 | 0.01 | 0.03 | 0.13 | 0.05 | 0.02 | 0.06 | -0.04 | 0.04 | Fasting glucose |
| rs174576 | 11 | 61603510 | 1.00 | C | A | 0.03 | 0.00 | -0.01 | 0.03 | -0.05 | 0.05 | 0.00 | 0.05 | 0.00 | 0.03 | Fasting glucose |
| rs11603334 | 11 | 72432985 | 1.00 | G | A | 0.03 | 0.00 | 0.01 | 0.03 | 0.04 | 0.06 | -0.03 | 0.07 | 0.01 | 0.05 | Fasting glucose |
| rs11020124 | 11 | 92690661 | 0.99 | C | T | 0.08 | 0.00 | 0.00 | 0.03 | 0.10 | 0.05 | -0.05 | 0.05 | -0.03 | 0.04 | Fasting glucose |
| rs2657879 | 12 | 56865338 | 0.99 | G | A | 0.02 | 0.00 | -0.04 | 0.03 | 0.02 | 0.06 | -0.14 | 0.06 | -0.03 | 0.04 | Fasting glucose |
| rs10747083 | 12 | 133041618 | 0.97 | A | G | 0.02 | 0.00 | -0.04 | 0.03 | -0.02 | 0.05 | -0.03 | 0.05 | -0.04 | 0.04 | Fasting glucose |
| rs11619319 | 13 | 28487599 | 1.00 | G | A | 0.03 | 0.00 | -0.12 | 0.03 | -0.06 | 0.06 | -0.12 | 0.06 | -0.16 | 0.04 | Fasting glucose |
| rs576674 | 13 | 33554302 | 1.00 | G | A | 0.02 | 0.00 | 0.03 | 0.03 | -0.07 | 0.06 | 0.07 | 0.07 | 0.05 | 0.04 | Fasting glucose |
| rs3783347 | 14 | 100839261 | 0.99 | G | T | 0.02 | 0.00 | 0.09 | 0.03 | 0.06 | 0.06 | 0.22 | 0.06 | 0.05 | 0.04 | Fasting glucose |
| rs4502156 | 15 | 62383155 | 0.98 | T | C | 0.03 | 0.00 | -0.04 | 0.02 | 0.04 | 0.05 | -0.04 | 0.05 | -0.07 | 0.03 | Fasting glucose |
| rs12440695 | 15 | 62435156 | 1.00 | T | C | 0.01 | 0.00 | 0.03 | 0.02 | 0.08 | 0.05 | 0.03 | 0.05 | 0.00 | 0.03 | Fasting glucose |
| rs6113722 | 20 | 22557099 | 0.99 | G | A | 0.05 | 0.01 | -0.02 | 0.06 | 0.10 | 0.12 | -0.07 | 0.11 | -0.04 | 0.08 | Fasting glucose |
| rs6072275 | 20 | 39743905 | 1.00 | A | G | 0.02 | 0.00 | 0.01 | 0.03 | 0.09 | 0.07 | -0.04 | 0.07 | 0.00 | 0.05 | Fasting glucose |
| rs4846565 | 1 | 219722104 | 1.00 | G | A | 0.03 | 0.00 | 0.05 | 0.03 | -0.01 | 0.05 | 0.10 | 0.05 | 0.06 | 0.03 | Fasting insulin |
| rs1530559 | 2 | 135755629 | 0.87 | A | G | 0.03 | 0.00 | 0.01 | 0.03 | 0.10 | 0.05 | 0.08 | 0.05 | -0.06 | 0.04 | Fasting insulin |
| rs10195252 | 2 | 165513091 | 1.00 | T | C | 0.03 | 0.00 | 0.02 | 0.02 | 0.05 | 0.05 | 0.07 | 0.05 | -0.02 | 0.03 | Fasting insulin |
| rs2943645 | 2 | 227099180 | 1.00 | T | C | 0.02 | 0.00 | 0.00 | 0.02 | -0.03 | 0.05 | 0.04 | 0.05 | -0.01 | 0.03 | Fasting insulin |
| rs17036328 | 3 | 12390484 | 1.00 | T | C | 0.03 | 0.01 | 0.03 | 0.04 | 0.03 | 0.07 | 0.04 | 0.07 | 0.03 | 0.05 | Fasting insulin |
| rs3822072 | 4 | 89741269 | 0.99 | A | G | 0.02 | 0.00 | 0.00 | 0.02 | 0.05 | 0.05 | 0.01 | 0.05 | -0.02 | 0.03 | Fasting insulin |
| rs974801 | 4 | 106071064 | 1.00 | G | A | 0.03 | 0.00 | 0.02 | 0.02 | 0.00 | 0.05 | 0.01 | 0.05 | 0.03 | 0.03 | Fasting insulin |
| rs6822892 | 4 | 157734675 | 0.99 | A | G | 0.02 | 0.00 | 0.03 | 0.02 | 0.03 | 0.05 | 0.02 | 0.05 | 0.03 | 0.03 | Fasting insulin |
| rs4865796 | 5 | 53272664 | 0.99 | A | G | 0.03 | 0.00 | 0.02 | 0.03 | 0.05 | 0.05 | 0.08 | 0.05 | -0.03 | 0.04 | Fasting insulin |
| rs459193 | 5 | 55806751 | 0.98 | G | A | 0.03 | 0.00 | 0.02 | 0.03 | 0.11 | 0.06 | -0.07 | 0.06 | 0.02 | 0.04 | Fasting insulin |
| rs6912327 | 6 | 34764922 | 1.00 | T | C | 0.03 | 0.01 | -0.09 | 0.03 | -0.11 | 0.06 | -0.09 | 0.06 | -0.07 | 0.04 | Fasting insulin |
| rs2745353 | 6 | 127452935 | 1.00 | T | C | 0.03 | 0.00 | 0.04 | 0.02 | 0.01 | 0.05 | 0.06 | 0.05 | 0.05 | 0.03 | Fasting insulin |
| rs1167800 | 7 | 75176196 | 0.99 | A | G | 0.03 | 0.00 | 0.05 | 0.02 | -0.01 | 0.05 | 0.08 | 0.05 | 0.08 | 0.03 | Fasting insulin |
| rs2126259 | 8 | 9185146 | 1.00 | T | C | 0.05 | 0.01 | -0.13 | 0.04 | -0.24 | 0.08 | -0.01 | 0.09 | -0.12 | 0.06 | Fasting insulin |
| rs7903146 | 10 | 114758349 | 1.00 | C | T | 0.03 | 0.01 | -0.01 | 0.03 | 0.04 | 0.05 | -0.03 | 0.05 | -0.02 | 0.04 | Fasting insulin |
| rs1421085 | 16 | 53800954 | 1.00 | C | T | 0.04 | 0.00 | 0.06 | 0.02 | 0.11 | 0.05 | 0.01 | 0.05 | 0.05 | 0.03 | Fasting insulin |
| rs731839 | 19 | 33899065 | 0.99 | G | A | 0.03 | 0.00 | 0.01 | 0.03 | 0.03 | 0.05 | 0.07 | 0.05 | -0.03 | 0.03 | Fasting insulin |
| rs1260326 | 2 | 27730940 | 1.00 | T | C | 0.03 | 0.01 | -0.02 | 0.02 | -0.01 | 0.05 | -0.08 | 0.05 | 0.01 | 0.03 | Glucose post 2-hours |
| rs2877716 | 3 | 123094451 | 1.00 | C | T | 0.05 | 0.01 | 0.02 | 0.03 | 0.01 | 0.06 | 0.13 | 0.06 | -0.02 | 0.04 | Glucose post 2-hours |
| rs7651090 | 3 | 185513392 | 1.00 | G | A | 0.03 | 0.01 | 0.00 | 0.03 | 0.05 | 0.05 | -0.05 | 0.05 | -0.01 | 0.04 | Glucose post 2-hours |
| rs1019503 | 5 | 96254817 | 1.00 | A | G | 0.04 | 0.01 | -0.03 | 0.02 | -0.01 | 0.05 | -0.04 | 0.05 | -0.04 | 0.03 | Glucose post 2-hours |
| rs6975024 | 7 | 44231886 | 1.00 | C | T | 0.06 | 0.01 | 0.04 | 0.03 | 0.07 | 0.06 | 0.07 | 0.06 | 0.02 | 0.04 | Glucose post 2-hours |
| rs11782386 | 8 | 9201787 | 0.99 | C | T | 0.06 | 0.01 | 0.08 | 0.04 | 0.12 | 0.08 | 0.00 | 0.09 | 0.09 | 0.05 | Glucose post 2-hours |
| rs12255372 | 10 | 114808902 | 1.00 | T | G | 0.05 | 0.01 | 0.01 | 0.03 | -0.05 | 0.05 | 0.04 | 0.05 | 0.03 | 0.04 | Glucose post 2-hours |
| rs1436958 | 15 | 62338797 | 1.00 | T | G | 0.03 | 0.01 | 0.02 | 0.02 | -0.04 | 0.05 | -0.02 | 0.05 | 0.07 | 0.03 | Glucose post 2-hours |
| rs11672660 | 19 | 46180184 | 1.00 | T | C | 0.07 | 0.01 | -0.02 | 0.03 | -0.01 | 0.06 | -0.05 | 0.06 | -0.02 | 0.04 | Glucose post 2-hours |
| rs340874 | 1 | 214159256 | 1.00 | C | T | 0.07 | 0.01 | 0.04 | 0.02 | 0.01 | 0.05 | 0.03 | 0.05 | 0.06 | 0.03 | Type 2 diabetes |
| rs780094 | 2 | 27741237 | 1.00 | C | T | 0.06 | 0.01 | 0.01 | 0.02 | 0.00 | 0.05 | 0.08 | 0.05 | -0.02 | 0.03 | Type 2 diabetes |
| rs77981966 | 2 | 43777964 | 0.98 | C | T | 0.15 | 0.02 | -0.07 | 0.04 | -0.10 | 0.09 | -0.16 | 0.09 | -0.02 | 0.06 | Type 2 diabetes |
| rs243020 | 2 | 60585028 | 1.00 | G | A | 0.06 | 0.01 | -0.03 | 0.02 | -0.12 | 0.05 | -0.03 | 0.05 | 0.01 | 0.03 | Type 2 diabetes |
| rs75297654 | 2 | 165545615 | 1.00 | C | T | 0.10 | 0.01 | 0.01 | 0.04 | 0.09 | 0.07 | -0.05 | 0.07 | 0.00 | 0.05 | Type 2 diabetes |
| rs2943645 | 2 | 227099180 | 1.00 | T | C | 0.09 | 0.01 | 0.00 | 0.02 | -0.03 | 0.05 | 0.04 | 0.05 | -0.01 | 0.03 | Type 2 diabetes |
| rs17036160 | 3 | 12329783 | 0.99 | C | T | 0.13 | 0.01 | 0.03 | 0.04 | 0.04 | 0.07 | 0.02 | 0.08 | 0.02 | 0.05 | Type 2 diabetes |
| rs17676309 | 3 | 64730121 | 0.99 | C | T | 0.07 | 0.01 | 0.03 | 0.02 | 0.11 | 0.05 | 0.01 | 0.05 | 0.00 | 0.03 | Type 2 diabetes |
| rs11708067 | 3 | 123065778 | 0.99 | A | G | 0.10 | 0.01 | 0.00 | 0.03 | 0.00 | 0.06 | 0.09 | 0.06 | -0.03 | 0.04 | Type 2 diabetes |
| rs35510946 | 3 | 185518910 | 1.00 | A | G | 0.13 | 0.01 | 0.00 | 0.03 | 0.05 | 0.05 | -0.05 | 0.05 | -0.01 | 0.04 | Type 2 diabetes |
| rs1046314 | 4 | 6303955 | 0.99 | A | G | 0.09 | 0.01 | -0.02 | 0.02 | 0.03 | 0.05 | -0.07 | 0.05 | -0.02 | 0.03 | Type 2 diabetes |
| rs7732130 | 5 | 76435004 | 0.99 | G | A | 0.08 | 0.01 | 0.01 | 0.03 | 0.10 | 0.05 | -0.05 | 0.05 | -0.01 | 0.04 | Type 2 diabetes |
| rs35261542 | 6 | 20675792 | 1.00 | A | C | 0.16 | 0.01 | -0.01 | 0.03 | -0.02 | 0.05 | -0.05 | 0.06 | 0.02 | 0.04 | Type 2 diabetes |
| rs10276674 | 7 | 14922007 | 0.98 | C | T | 0.08 | 0.01 | 0.05 | 0.03 | -0.01 | 0.06 | 0.16 | 0.06 | 0.04 | 0.04 | Type 2 diabetes |
| rs1974620 | 7 | 15065467 | 0.98 | T | C | 0.06 | 0.01 | 0.00 | 0.02 | -0.06 | 0.05 | 0.06 | 0.05 | 0.01 | 0.03 | Type 2 diabetes |
| rs1513272 | 7 | 28200097 | 0.98 | C | T | 0.10 | 0.01 | -0.02 | 0.02 | -0.09 | 0.05 | -0.14 | 0.05 | 0.06 | 0.03 | Type 2 diabetes |
| rs878521 | 7 | 44255643 | 0.98 | A | G | 0.07 | 0.01 | 0.02 | 0.03 | 0.07 | 0.06 | 0.01 | 0.06 | 0.00 | 0.04 | Type 2 diabetes |
| rs13266634 | 8 | 118184783 | 1.00 | C | T | 0.11 | 0.01 | 0.01 | 0.03 | 0.06 | 0.05 | -0.03 | 0.05 | 0.01 | 0.04 | Type 2 diabetes |
| rs10974438 | 9 | 4291928 | 0.98 | C | A | 0.07 | 0.01 | -0.05 | 0.02 | -0.02 | 0.05 | -0.02 | 0.05 | -0.07 | 0.03 | Type 2 diabetes |
| rs10811660 | 9 | 22134068 | 0.97 | G | A | 0.24 | 0.02 | 0.00 | 0.03 | -0.05 | 0.06 | 0.10 | 0.07 | -0.02 | 0.04 | Type 2 diabetes |
| rs10757283 | 9 | 22134172 | 0.99 | T | C | 0.11 | 0.01 | -0.01 | 0.02 | -0.05 | 0.05 | -0.04 | 0.05 | 0.03 | 0.03 | Type 2 diabetes |
| rs11187140 | 10 | 94466910 | 0.99 | G | A | 0.11 | 0.01 | -0.06 | 0.02 | -0.01 | 0.05 | -0.06 | 0.05 | -0.09 | 0.03 | Type 2 diabetes |
| rs7903146 | 10 | 114758349 | 1.00 | T | C | 0.31 | 0.01 | 0.01 | 0.03 | -0.04 | 0.05 | 0.03 | 0.05 | 0.02 | 0.04 | Type 2 diabetes |
| rs231360 | 11 | 2692249 | 0.98 | T | C | 0.08 | 0.01 | 0.00 | 0.02 | 0.04 | 0.05 | -0.04 | 0.05 | 0.00 | 0.03 | Type 2 diabetes |
| rs2283220 | 11 | 2755548 | 0.98 | A | G | 0.06 | 0.01 | 0.01 | 0.03 | -0.01 | 0.05 | -0.05 | 0.05 | 0.04 | 0.04 | Type 2 diabetes |
| rs2237895 | 11 | 2857194 | 0.94 | C | A | 0.07 | 0.01 | 0.03 | 0.02 | 0.00 | 0.05 | 0.01 | 0.05 | 0.05 | 0.03 | Type 2 diabetes |
| rs74046911 | 11 | 2858636 | 0.96 | C | T | 0.25 | 0.02 | 0.07 | 0.06 | 0.13 | 0.11 | -0.06 | 0.13 | 0.08 | 0.08 | Type 2 diabetes |
| rs5215 | 11 | 17408630 | 1.00 | C | T | 0.07 | 0.01 | -0.03 | 0.02 | -0.03 | 0.05 | 0.02 | 0.05 | -0.05 | 0.03 | Type 2 diabetes |
| rs74333814 | 11 | 72457487 | 0.99 | C | T | 0.10 | 0.01 | 0.01 | 0.03 | 0.04 | 0.07 | -0.03 | 0.07 | 0.01 | 0.05 | Type 2 diabetes |
| rs2583941 | 12 | 66204598 | 1.00 | A | G | 0.10 | 0.01 | 0.00 | 0.04 | 0.01 | 0.08 | -0.02 | 0.08 | 0.00 | 0.05 | Type 2 diabetes |
| rs7961581 | 12 | 71663102 | 0.99 | C | T | 0.06 | 0.01 | 0.00 | 0.03 | -0.03 | 0.05 | -0.02 | 0.06 | 0.02 | 0.04 | Type 2 diabetes |
| rs1169288 | 12 | 121416650 | 0.98 | C | A | 0.09 | 0.01 | 0.12 | 0.03 | 0.19 | 0.05 | 0.13 | 0.05 | 0.07 | 0.03 | Type 2 diabetes |
| rs1800574 | 12 | 121416864 | 0.92 | T | C | 0.20 | 0.03 | 0.06 | 0.07 | 0.06 | 0.15 | -0.13 | 0.15 | 0.14 | 0.10 | Type 2 diabetes |
| rs7172432 | 15 | 62396389 | 1.00 | A | G | 0.06 | 0.01 | -0.04 | 0.02 | 0.02 | 0.05 | -0.04 | 0.05 | -0.07 | 0.03 | Type 2 diabetes |
| rs3803563 | 15 | 91531352 | 1.00 | A | C | 0.08 | 0.01 | -0.08 | 0.03 | -0.05 | 0.06 | -0.10 | 0.06 | -0.09 | 0.04 | Type 2 diabetes |
| rs7193144 | 16 | 53810686 | 1.00 | C | T | 0.13 | 0.01 | 0.05 | 0.02 | 0.11 | 0.05 | 0.00 | 0.05 | 0.05 | 0.03 | Type 2 diabetes |
| rs4430796 | 17 | 36098040 | 1.00 | G | A | 0.09 | 0.01 | 0.00 | 0.02 | 0.04 | 0.05 | 0.02 | 0.05 | -0.02 | 0.03 | Type 2 diabetes |
| rs7234864 | 18 | 57734857 | 1.00 | T | C | 0.06 | 0.01 | 0.02 | 0.03 | 0.04 | 0.05 | 0.01 | 0.06 | 0.01 | 0.04 | Type 2 diabetes |
| rs17066842 | 18 | 58040624 | 0.99 | G | A | 0.11 | 0.02 | -0.04 | 0.06 | -0.02 | 0.12 | -0.16 | 0.13 | 0.00 | 0.08 | Type 2 diabetes |
| rs72999033 | 19 | 19366632 | 0.96 | T | C | 0.15 | 0.02 | 0.07 | 0.05 | 0.10 | 0.10 | 0.03 | 0.10 | 0.07 | 0.07 | Type 2 diabetes |
| rs4399645 | 19 | 46166073 | 0.96 | T | C | 0.06 | 0.01 | 0.04 | 0.02 | 0.07 | 0.05 | 0.02 | 0.05 | 0.03 | 0.03 | Type 2 diabetes |
| rs2238689 | 19 | 46178661 | 0.98 | C | T | 0.08 | 0.01 | -0.01 | 0.02 | -0.04 | 0.05 | 0.02 | 0.05 | -0.01 | 0.03 | Type 2 diabetes |
| rs1800961 | 20 | 43042364 | 0.99 | T | C | 0.15 | 0.03 | -0.14 | 0.07 | -0.18 | 0.14 | -0.11 | 0.15 | -0.14 | 0.10 | Type 2 diabetes |

* BMI: Body mass index; Chr: Chromosome; HDL: High-density lipoprotein cholesterol; LDL: Low-density lipoprotein cholesterol; SE: Standard error; SNP: Single nucleotide polymorphism.

**Supplementary Table 3. Power assessment to validate previously observed risk from potential risk parameters using Mendelian randomization analysis**

| **Exposure** | **Previous observed risk increase** | | | |  | **Power assessment** | |  |
| --- | --- | --- | --- | --- | --- | --- | --- | --- |
|  | **Units** | **Risk increase** | **95% Confidence Interval** | **Publication** |  | **Standard deviation of the exposure** | **Risk increase per standard deviation** | **Mendelian randomization power (%)** |
| Height (cm) | 5 | 1.07 | 1.03-1.12 | Aune et al. 2012b (10) |  | 6.9 | 1.10 | 57.7 |
| Body mass index | 5 | 1.10 | 1.07-1.14 | Aune et al. 2012a (8) |  | 4.6 | 1.09 | 12.3 |
| Body mass index | 5 | 1.43 | 1.14-180 | Urayama et al 2011 (9) |  | 4.6 | 1.39 | 86.3 |
| Waist to hip ratio | 0.1 | 1.19 | 1.09-1.31 | Aune et al. 2012 (8) |  | 0.1 | 1.19 | 21.2 |
| Fasting glucose (mmol/L) | 0.56 | 1.14 | 1.06-1.22 | Liao et al. 2014 (12) |  | 0.8 | 1.21 | 65.5 |
| Type 2 diabetes status | - | 1.40 | 1.07-1.84 | Elena et al. 2013 (13) |  | - | 1.40 | 99.5 |

**Supplementary Table 4. Risk increase on pancreatic cancer excluding SNPs also robustly associated with body mass index (BMI)**

| **Phenotype** | **No. of SNPs** | **P_Heterogeneity_*** | **Likelihood-based estimate** | |
| --- | --- | --- | --- | --- |
|  |  |  | **OR (LCI, UCI)** | **P†** |
| High density cholesterol | 67 | 0.01 | 1.08 (0.95 to 1.22) | 0.25 |
| Low density cholesterol | 53 | 0.004 | 0.99 (0.87 to 1.12) | 0.87 |
| Total cholesterol | 71 | 0.001 | 1.03 (0.90 to 1.16) | 0.70 |
| Triglycerides | 38 | 0.09 | 0.89 (0.76 to 1.04) | 0.14 |
| Fasting glucose | 36 | <0.001 | 0.93 (0.71 to 1.20) | 0.57 |
| Fasting insulin | 14 | 0.005 | 1.26 (0.73 to 2.16) | 0.41 |
| 2h post-challenge glucose | 7 | 0.24 | 1.32 (0.81 to 2.15) | 0.27 |
| Type 2 diabetes | 39 | 0.001 | 1.00 (0.92 to 1.10) | 0.92 |

***** Heterogeneity Q test**.** P_Heterogeneity_: Two-sided P value of heterogeneity between instrumental SNP causal estimates (β_GD_/β_GP_) from genetic effects in **Supplementary Table 1**.

**†** Likelihood-based Mendelian randomization test. P: Two-sided P value.

OR: Odds ratio; LCI: 95% lower confidence interval; UCI: 95% upper confidence interval

**Supplementary Table 5. Risk increase on pancreatic cancer provided by the weighted median approach and overall pleiotropic effect assessment provided by MR-Egger test**

| **Phenotype** | **No. of SNPs** | **P_Heterogeneity_*** | **Weighted median estimator** | | |  | **MR-Egger intercept** | | |  | **MR-Egger risk estimate** | | |
| --- | --- | --- | --- | --- | --- | --- | --- | --- | --- | --- | --- | --- | --- |
|  |  |  | **OR** | **(LCI,UCI)** | **P†** |  | **Est** | **(LCI,UCI)** | **P**‡ |  | **OR** | **(LCI,UCI)** | **P**§ |
| Height | 558 | <0.001 | 1.14 | (1.00 to 1.29) | 0.05 |  | -4.1x10^-3^ | (-0.01 to 2.0x10^-3^) | 0.19 |  | 1.18 | (0.95 to 1.45) | 0.13 |
| Body mass index | 95 | <0.001 | 1.70 | (1.22 to 2.36) | 0.002 |  | -3.1x10^-3^ | (-0.02 to 0.01) | 0.64 |  | 1.48 | (0.90 to 2.44) | 0.12 |
| Waist to hip ratio | 34 | 0.19 | 1.62 | (0.92 to 2.85) | 0.10 |  | -0.04 | (-0.07 to -3.1x10^-4^) | 0.05 |  | 4.63 | (1.07 to 19.94) | 0.04 |
| High density cholesterol | 70 | 0.002 | 1.06 | (0.86 to 1.29) | 0.61 |  | -0.01 | (-0.02 to 1.7x10^-3^) | 0.10 |  | 1.22 | (1.00 to 1.49) | 0.05 |
| Low density cholesterol | 54 | 0.003 | 1.00 | (0.82 to 1.21) | 0.98 |  | 2.3x10^-3^ | (-0.01 to 0.01) | 0.71 |  | 0.94 | (0.77 to 1.16) | 0.56 |
| Total cholesterol | 72 | <0.001 | 0.99 | (0.81 to 1.21) | 0.91 |  | 0.01 | (-0.01 to 0.02) | 0.30 |  | 0.91 | (0.73 to 1.14) | 0.42 |
| Triglycerides | 39 | 0.03 | 0.84 | (0.67 to 1.06) | 0.15 |  | 0.02 | (0.01 to 0.04) | 0.002 |  | 0.63 | (0.48 to 0.83) | 0.001 |
| Fasting glucose | 37 | <0.001 | 0.97 | (0.66 to 1.43) | 0.89 |  | 0.01 | (-0.01 to 0.03) | 0.29 |  | 0.74 | (0.45 to 1.24) | 0.25 |
| Fasting insulin | 17 | 0.002 | 1.94 | (1.02 to 3.68) | 0.04 |  | 0.05 | (-0.01 to 0.12) | 0.11 |  | 0.23 | (0.02 to 2.72) | 0.22 |
| 2h post-challenge glucose | 9 | 0.28 | 1.15 | (0.68 to 1.95) | 0.62 |  | -0.03 | (-0.11 to 0.06) | 0.49 |  | 1.91 | (0.33 to 11.05) | 0.41 |
| Type 2 diabetes | 43 | 0.001 | 1.03 | (0.91 to 1.17) | 0.68 |  | 4.9x10^-4^ | (-0.02 to 0.02) | 0.96 |  | 1.02 | (0.86 to 1.22) | 0.78 |

***** Heterogeneity Q test**.** P_Heterogeneity_: Two-sided P value of heterogeneity between instrumental SNP causal estimates (β_GD_/β_GP_) from genetic effects in **Supplementary Table 1**..

† Weighted median Mendelian randomization test. P: Two-sided P value.

‡ Mendelian randomization – Egger intercept test. P: Two-sided P value.

§ Mendelian randomization – Egger test. P: Two-sided P value.

OR: Odds ratio; Est: Estimate; LCI: 95% lower confidence interval; UCI: 95% upper confidence interval

**Supplementary Table 6. Risk increase from mechanistic pathways components of risk factors on pancreatic cancer.**

| **Factor** | **Mechanistic pathway** | **No. of SNPs** | **OR** | **(LCI, UCI)** | **P*** |
| --- | --- | --- | --- | --- | --- |
| **Height** | Abnormal Skeleton Morphology | 40 | 1.11 | (0.82 to 1.51) | 0.51 |
|  | Growth Factor Binding | 38 | 1.25 | (0.95 to 1.64) | 0.11 |
|  | Abnormal Bone Ossification | 30 | 1.01 | (0.70 to 1.48) | 0.94 |
|  | Short Mandible | 29 | 0.94 | (0.65 to 1.37) | 0.75 |
|  | Abnormal Middle Ear Ossicle Morphology | 28 | 0.99 | (0.71 to 1.39) | 0.97 |
|  | Short Snout | 27 | 1.01 | (0.70 to 1.44) | 0.98 |
|  | TGFB1 protein complex | 27 | 1.16 | (0.85 to 1.59) | 0.35 |
|  | Abnormal Cartilage Morphology | 26 | 0.75 | (0.53 to 1.06) | 0.11 |
|  | Short Limbs | 26 | 0.91 | (0.63 to 1.32) | 0.61 |
|  | Abnormal Rib Morphology | 25 | 0.91 | (0.65 to 1.27) | 0.59 |
|  | Short Ulna | 25 | 0.87 | (0.61 to 1.24) | 0.43 |
|  | Tgf Beta Signaling Pathway | 25 | 1.11 | (0.80 to 1.56) | 0.53 |
|  | Delayed Bone Ossification | 24 | 1.02 | (0.69 to 1.49) | 0.94 |
|  | Delayed Endochondral Bone Ossification | 24 | 0.87 | (0.60 to 1.27) | 0.48 |
|  | Short Nasal Bone | 23 | 0.94 | (0.65 to 1.38) | 0.76 |
|  | Abnormal Forelimb Morphology | 22 | 1.01 | (0.69 to 1.47) | 0.98 |
|  | Abnormal Long Bone Morphology | 22 | 0.78 | (0.52 to 1.18) | 0.24 |
|  | Abnormal Skeleton Development | 22 | 0.90 | (0.60 to 1.33) | 0.58 |
|  | Decreased Length Of Long Bones | 22 | 0.85 | (0.55 to 1.30) | 0.45 |
|  | Abnormal Femur Morphology | 21 | 0.78 | (0.50 to 1.19) | 0.25 |
|  | Abnormal Vertebrae Morphology | 21 | 0.90 | (0.57 to 1.43) | 0.67 |
|  | Abnormal Axial Skeleton Morphology | 20 | 0.82 | (0.55 to 1.23) | 0.34 |
|  | Abnormal Fibula Morphology | 19 | 0.93 | (0.59 to 1.46) | 0.74 |
|  | Cleft Secondary Palate | 19 | 1.00 | (0.64 to 1.58) | 0.99 |
|  | Abnormal Craniofacial Bone Morphology | 18 | 1.32 | (0.81 to 2.13) | 0.27 |
|  | Cleft Palate | 18 | 0.97 | (0.64 to 1.49) | 0.90 |
|  | Complete Neonatal Lethality | 18 | 0.84 | (0.53 to 1.33) | 0.45 |
|  | Wnt-Protein Binding | 18 | 0.96 | (0.61 to 1.51) | 0.87 |
|  | Abnormal Neurocranium Morphology | 17 | 0.95 | (0.59 to 1.53) | 0.83 |
|  | Basal Cell Carcinoma | 17 | 0.75 | (0.48 to 1.17) | 0.20 |
|  | Abnormal Blood Vessel Morphology | 16 | 1.28 | (0.80 to 2.04) | 0.31 |
|  | Abnormal Frontal Bone Morphology | 16 | 0.97 | (0.61 to 1.55) | 0.91 |
|  | Abnormal Parietal Bone Morphology | 16 | 1.02 | (0.67 to 1.57) | 0.92 |
|  | Abnormal Tibia Morphology | 16 | 0.92 | (0.57 to 1.51) | 0.75 |
|  | Chromatin Binding | 16 | 1.56 | (0.94 to 2.60) | 0.09 |
|  | Complete Perinatal Lethality | 16 | 1.08 | (0.65 to 1.79) | 0.77 |
|  | Small Lung | 16 | 1.03 | (0.67 to 1.60) | 0.88 |
|  | Small Thoracic Cage | 16 | 0.81 | (0.50 to 1.32) | 0.40 |
|  | Abnormal Limb Morphology | 15 | 1.01 | (0.60 to 1.70) | 0.98 |
|  | Abnormal Vertebral Body Morphology | 15 | 0.73 | (0.44 to 1.21) | 0.23 |
|  | Complete Embryonic Lethality During Organogenesis | 15 | 1.01 | (0.60 to 1.67) | 0.98 |
|  | Embryonic Growth Retardation | 15 | 0.92 | (0.55 to 1.53) | 0.74 |
|  | Abnormal Xiphoid Process Morphology | 14 | 0.94 | (0.55 to 1.61) | 0.83 |
|  | Disproportionate Dwarf | 14 | 0.76 | (0.44 to 1.31) | 0.32 |
|  | Transcription Regulatory Region Dna Binding | 14 | 0.77 | (0.48 to 1.24) | 0.29 |
|  | Wnt-Activated Receptor Activity | 14 | 1.24 | (0.75 to 2.03) | 0.40 |
|  | Abnormal Cranium Morphology | 13 | 0.96 | (0.49 to 1.85) | 0.89 |
|  | Pathways In Cancer | 13 | 1.22 | (0.72 to 2.07) | 0.46 |
|  | Regulatory Region Dna Binding | 13 | 0.86 | (0.52 to 1.40) | 0.54 |
|  | Regulatory Region Nucleic Acid Binding | 13 | 0.86 | (0.52 to 1.40) | 0.54 |
|  | Small Basisphenoid Bone | 13 | 1.34 | (0.88 to 2.05) | 0.18 |
|  | Abnormal Basisphenoid Bone Morphology | 12 | 1.06 | (0.62 to 1.83) | 0.83 |
|  | Abnormal Neural Tube MorphologyDevelopment | 12 | 0.84 | (0.49 to 1.44) | 0.53 |
|  | Hemorrhage | 12 | 1.11 | (0.62 to 1.98) | 0.73 |
|  | Partial Lethality Throughout Fetal Growth And Development | 12 | 0.88 | (0.48 to 1.62) | 0.68 |
|  | Shortened Head | 12 | 0.92 | (0.51 to 1.67) | 0.79 |
|  | SP1 protein complex | 12 | 1.36 | (0.74 to 2.50) | 0.33 |
|  | Transcription Factor Binding | 12 | 1.48 | (0.84 to 2.60) | 0.17 |
|  | Abnormal Craniofacial Morphology | 11 | 1.06 | (0.61 to 1.83) | 0.84 |
|  | Domed Cranium | 11 | 0.89 | (0.48 to 1.64) | 0.70 |
|  | Partial Postnatal Lethality | 11 | 1.04 | (0.61 to 1.77) | 0.88 |
|  | LRP6 protein complex | 10 | 1.29 | (0.67 to 2.50) | 0.45 |
|  | Rib Fusion | 10 | 0.95 | (0.56 to 1.61) | 0.84 |
|  | Abnormal Extraembryonic Tissue Morphology | 9 | 1.15 | (0.58 to 2.29) | 0.68 |
|  | Abnormal Tooth Development | 9 | 0.88 | (0.44 to 1.77) | 0.72 |
|  | AR protein complex | 9 | 0.99 | (0.50 to 1.96) | 0.98 |
|  | Cartilage Development | 9 | 0.69 | (0.41 to 1.13) | 0.14 |
|  | Decreased Cell Proliferation | 9 | 1.08 | (0.55 to 2.12) | 0.83 |
|  | Decreased Cranium Height | 9 | 0.99 | (0.53 to 1.85) | 0.98 |
|  | HDAC1 protein complex | 9 | 0.93 | (0.49 to 1.78) | 0.83 |
|  | Pi-3K Cascade | 9 | 0.92 | (0.52 to 1.61) | 0.76 |
|  | Renal Hypoplasia | 9 | 1.33 | (0.76 to 2.31) | 0.32 |
|  | Structure-Specific Dna Binding | 9 | 1.71 | (0.85 to 3.43) | 0.13 |
|  | SUFU protein complex | 9 | 0.95 | (0.54 to 1.67) | 0.86 |
|  | TGFBR2 protein complex | 9 | 1.23 | (0.61 to 2.50) | 0.56 |
|  | Abnormal Eye Development | 8 | 0.79 | (0.43 to 1.47) | 0.46 |
|  | Abnormal Head Morphology | 8 | 0.90 | (0.47 to 1.71) | 0.74 |
|  | Abnormal Vitelline Vasculature Morphology | 8 | 0.75 | (0.32 to 1.79) | 0.52 |
|  | CREBBP protein complex | 8 | 0.86 | (0.45 to 1.64) | 0.64 |
|  | Decreased Fetal Size | 8 | 0.87 | (0.47 to 1.60) | 0.64 |
|  | EP300 protein complex | 8 | 1.54 | (0.72 to 3.28) | 0.27 |
|  | HDAC3 protein complex | 8 | 1.12 | (0.58 to 2.17) | 0.74 |
|  | NCOR1 protein complex | 8 | 0.92 | (0.47 to 1.80) | 0.82 |
|  | Partial Perinatal Lethality | 8 | 1.44 | (0.58 to 3.61) | 0.43 |
|  | Short Maxilla | 8 | 1.18 | (0.55 to 2.52) | 0.68 |
|  | Smad Binding | 8 | 1.15 | (0.52 to 2.53) | 0.73 |
|  | SMAD2 protein complex | 8 | 1.07 | (0.47 to 2.45) | 0.87 |
|  | Abnormal Middle Ear Morphology | 7 | 0.81 | (0.43 to 1.54) | 0.53 |
|  | Complete Lethality Throughout Fetal Growth And Development | 7 | 1.72 | (0.77 to 3.82) | 0.19 |
|  | Decreased Embryo Size | 7 | 1.56 | (0.76 to 3.18) | 0.23 |
|  | Protein Binding Transcription Factor Activity | 7 | 1.31 | (0.53 to 3.22) | 0.56 |
|  | Sequence-Specific Dna Binding Rna Polymerase Ii Transcription Factor Activity | 7 | 1.29 | (0.61 to 2.72) | 0.50 |
|  | SMAD3 protein complex | 7 | 1.31 | (0.55 to 3.16) | 0.54 |
|  | SMAD7 protein complex | 7 | 0.92 | (0.43 to 1.98) | 0.83 |
|  | Transcription Cofactor Activity | 7 | 1.31 | (0.53 to 3.22) | 0.56 |
|  | Transcription Factor Binding Transcription Factor Activity | 7 | 0.65 | (0.27 to 1.57) | 0.33 |
|  | Transcription Factor Complex | 7 | 1.14 | (0.49 to 2.65) | 0.76 |
|  | Abnormal Head Shape | 6 | 0.85 | (0.41 to 1.78) | 0.67 |
|  | Abnormal Incisor Morphology | 6 | 0.99 | (0.40 to 2.45) | 0.99 |
|  | CBX3 protein complex | 6 | 1.45 | (0.65 to 3.22) | 0.37 |
|  | Decreased Body Height | 6 | 1.65 | (0.71 to 3.83) | 0.24 |
|  | Endochondral Bone Morphogenesis | 6 | 0.84 | (0.41 to 1.71) | 0.62 |
|  | ESR1 protein complex | 6 | 1.46 | (0.58 to 3.69) | 0.42 |
|  | Exencephaly | 6 | 0.82 | (0.34 to 1.98) | 0.66 |
|  | INS-IGF2 protein complex | 6 | 1.52 | (0.75 to 3.06) | 0.24 |
|  | Malocclusion | 6 | 0.93 | (0.44 to 1.98) | 0.85 |
|  | MYBL2 protein complex | 6 | 2.20 | (0.88 to 5.49) | 0.09 |
|  | Open Neural Tube | 6 | 0.89 | (0.36 to 2.19) | 0.80 |
|  | Partial Embryonic Lethality During Organogenesis | 6 | 1.31 | (0.52 to 3.32) | 0.57 |
|  | Rna Polymerase Ii Distal Enhancer Sequence-Specific Dna Binding Transcription Factor Activity | 6 | 0.85 | (0.42 to 1.70) | 0.64 |
|  | Skeletal System Development | 6 | 0.90 | (0.44 to 1.84) | 0.77 |
|  | Abnormal Cell Differentiation | 5 | 2.36 | (0.78 to 7.13) | 0.13 |
|  | Abnormal Cervical Atlas Morphology | 5 | 0.96 | (0.45 to 2.02) | 0.91 |
|  | Abnormal Sternum Morphology | 5 | 0.95 | (0.48 to 1.90) | 0.89 |
|  | Abnormal Ulna Morphology | 5 | 0.99 | (0.35 to 2.80) | 0.98 |
|  | Abnormal Visceral Yolk Sac Morphology | 5 | 0.77 | (0.33 to 1.81) | 0.55 |
|  | Bone Morphogenesis | 5 | 0.93 | (0.43 to 1.99) | 0.84 |
|  | Chronic Myeloid Leukemia | 5 | 1.00 | (0.42 to 2.34) | 0.99 |
|  | Complete Embryonic Lethality | 5 | 1.40 | (0.59 to 3.32) | 0.44 |
|  | Increased Apoptosis | 5 | 2.91 | (1.16 to 7.29) | 0.02 |
|  | Morphogenesis Of A Branching Structure | 5 | 1.55 | (0.66 to 3.65) | 0.32 |
|  | Negative Regulation Of Growth | 5 | 0.76 | (0.30 to 1.91) | 0.56 |
|  | Ossification | 5 | 1.24 | (0.57 to 2.70) | 0.60 |
|  | Regulation Of Canonical Wnt Receptor Signaling Pathway | 5 | 0.94 | (0.45 to 1.94) | 0.86 |
|  | SMAD9 protein complex | 5 | 0.95 | (0.40 to 2.26) | 0.90 |
|  | Small Cell Lung Cancer | 5 | 2.07 | (0.83 to 5.15) | 0.12 |
|  | TFDP1 protein complex | 5 | 2.72 | (1.06 to 7.00) | 0.04 |
| **Body-mass index** | Neuronal Developmental processes | 28 | 1.17 | (0.77 to 1.77) | 0.47 |
|  | Hypothalamic expression and regulatory function | 11 | 1.27 | (0.81 to 1.98) | 0.31 |
|  | Neuronal Expression | 11 | 1.41 | (0.72 to 2.76) | 0.32 |
|  | Lipid biosynthesis and metabolism | 10 | 1.31 | (0.59 to 2.89) | 0.51 |
|  | Neurotransmission | 9 | 1.90 | (0.91 to 3.95) | 0.09 |
|  | Bone Development | 8 | 2.34 | (1.03 to 5.32) | 0.04 |
|  | **Signalling** |  |  |  |  |
|  | Mitogen activated protein kinase1 Extracellular signal regulated kinases | 8 | 0.67 | (0.98 to 4.92) | 0.06 |
|  | CyclicAMP | 5 | 1.48 | (0.29 to 1.53) | 0.34 |
|  | Wnt | 5 | 1.34 | (0.75 to 2.92) | 0.25 |
|  | **Guanine Nucleotide Binding-Protein Coupled Receptors (GPCRs)** |  |  |  |  |
|  | Cell cycle | 19 | 1.87 | (0.77 to 2.31) | 0.30 |
|  | Endocytosis Exocytosis | 13 | 1.73 | (1.01 to 3.68) | 0.05 |
|  | Immune System | 13 | 1.29 | (0.98 to 3.58) | 0.06 |
|  | Apoptosis | 12 | 1.37 | (0.84 to 3.59) | 0.14 |
|  | Glucose homeostasis and or diabetes | 11 | 1.29 | (0.71 to 2.36) | 0.41 |
|  | Membrane Proteins | 11 | 0.52 | (0.81 to 2.30) | 0.24 |
|  | Tumorigenesis | 10 | 0.76 | (0.63 to 2.60) | 0.49 |
|  | Mitochondrial | 7 | 1.25 | (0.24 to 1.11) | 0.09 |
|  | Monogenic Obesity and or Energy Homeostasis | 7 | 1.43 | (0.42 to 1.40) | 0.38 |
|  | Retinoic Acid Receptors | 6 | 1.10 | (0.53 to 2.97) | 0.61 |
| **High-density lipoprotein cholesterol** | FXR/RXR activation | 7 | 1.10 | (1.05 to 1.96) | 0.02 |
|  | LPS IL-1 mediated inhibition of RXR function | 6 | 0.99 | (0.92 to 1.31) | 0.28 |
|  | Cholesterol metabolic process | 5 | 1.11 | (0.91 to 1.32) | 0.34 |
| **Low-density lipoprotein cholesterol** | Cholesterol metabolic process | 9 | 0.93 | (0.83 to 1.19) | 0.95 |
|  | FXR/RXR activation | 6 | 0.89 | (0.88 to 1.41) | 0.38 |
|  | Lipid transport | 6 | 1.06 | (0.77 to 1.13) | 0.48 |
| **Total cholesterol** | Steroid metabolic process | 9 | 1.76 | (0.69 to 1.15) | 0.38 |
|  | LPS IL-1 Mediated Inhibition of RXR Function | 7 | 0.85 | (0.81 to 1.39) | 0.66 |
|  | Hepatic cholestasis | 6 | 0.66 | (0.98 to 3.16) | 0.06 |
|  | PXR/RXR activation | 5 | 1.11 | (0.41 to 1.75) | 0.66 |
| **Triglycerides** | FXR/RXR activation | 5 | 1.25 | (0.46 to 0.96) | 0.03 |

* Likelihood-based Mendelian randomization test. P: Two-sided P value.

OR: Odds ratio; LCI: 95% lower confidence interval; UCI: 95% upper confidence interval

**Supplementary Figure 1. Power calculations in Mendelian randomization with the 7,110 cases and 7,265 controls for genetic instruments explaining different proportion of phenotype variance (10.0%, 5.0%, 2.7% and 1.5%).**

**
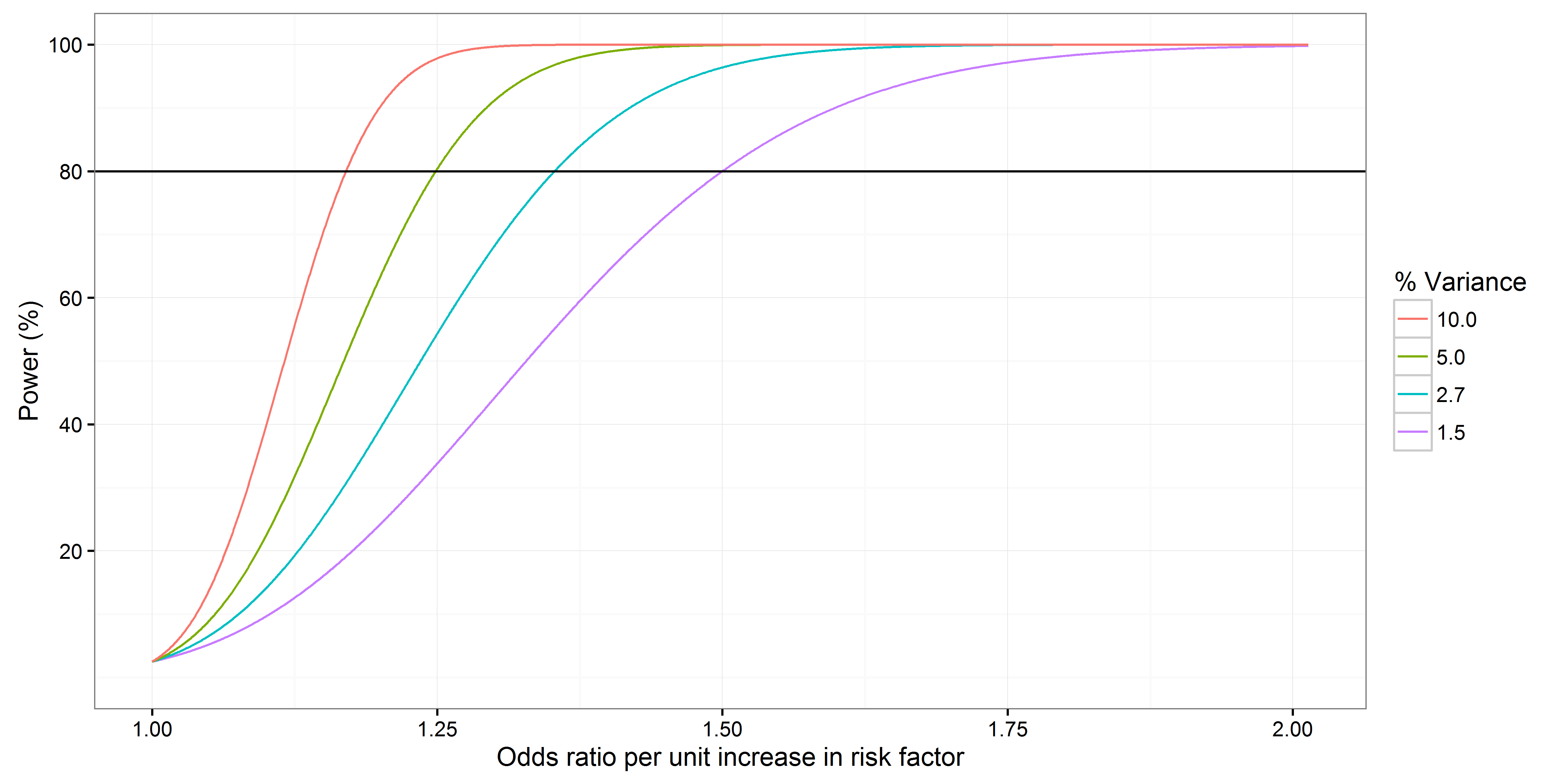
**

**Supplementary Figure 2. Forest plots of risk incrase on pancreatic cancer for each standard deviation increase in height (A), waist-to-hip ratio (B), HDL (C), LDL (D), fasting glucose (E) and 2h post-challenge glucose (F) stratified by publication sets and sex.**

**
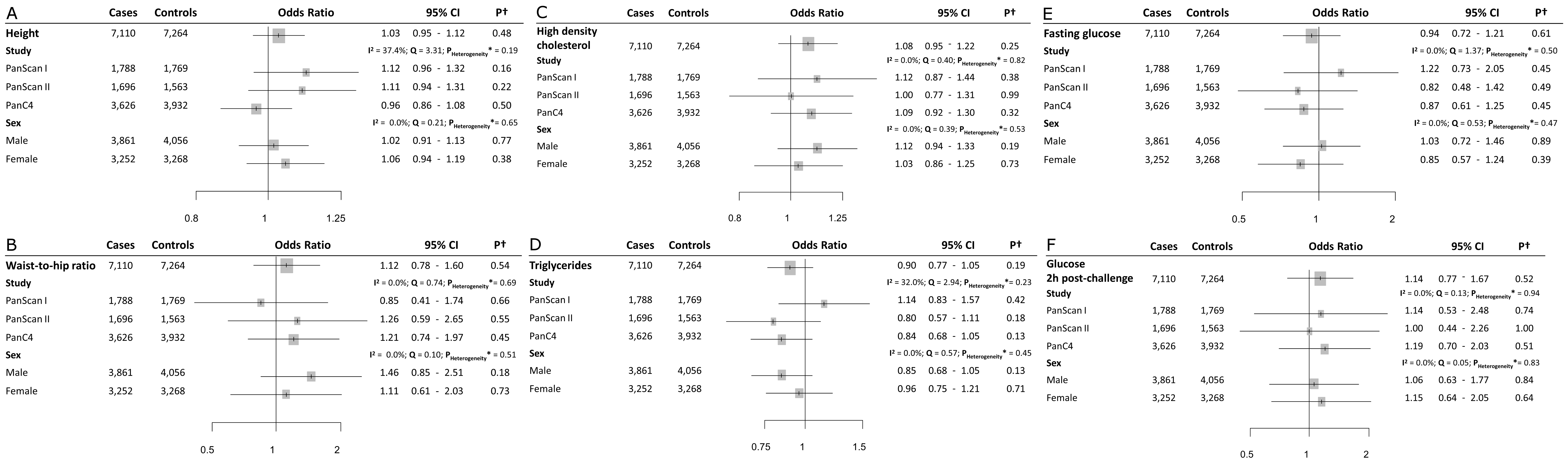
**

* Heterogeneity Q test**.** P_Heterogeneity_: Two-sided P value. I^2^: Index of between-strata heterogeneity; Q: Statistic for between-strata heterogeneity.

† Likelihood-based Mendelian randomization test. P: Two-sided P value. CI: Confidence interval.

**Supplementary Figure 3. Funnel plots of risk estimates of instrumental SNPs for height (A), waist-to-hip ratio (B), HDL (C), LDL (D), fasting glucose (E) and 2h post-challenge glucose (F) on pancreatic cancer against instrumental strength*.**


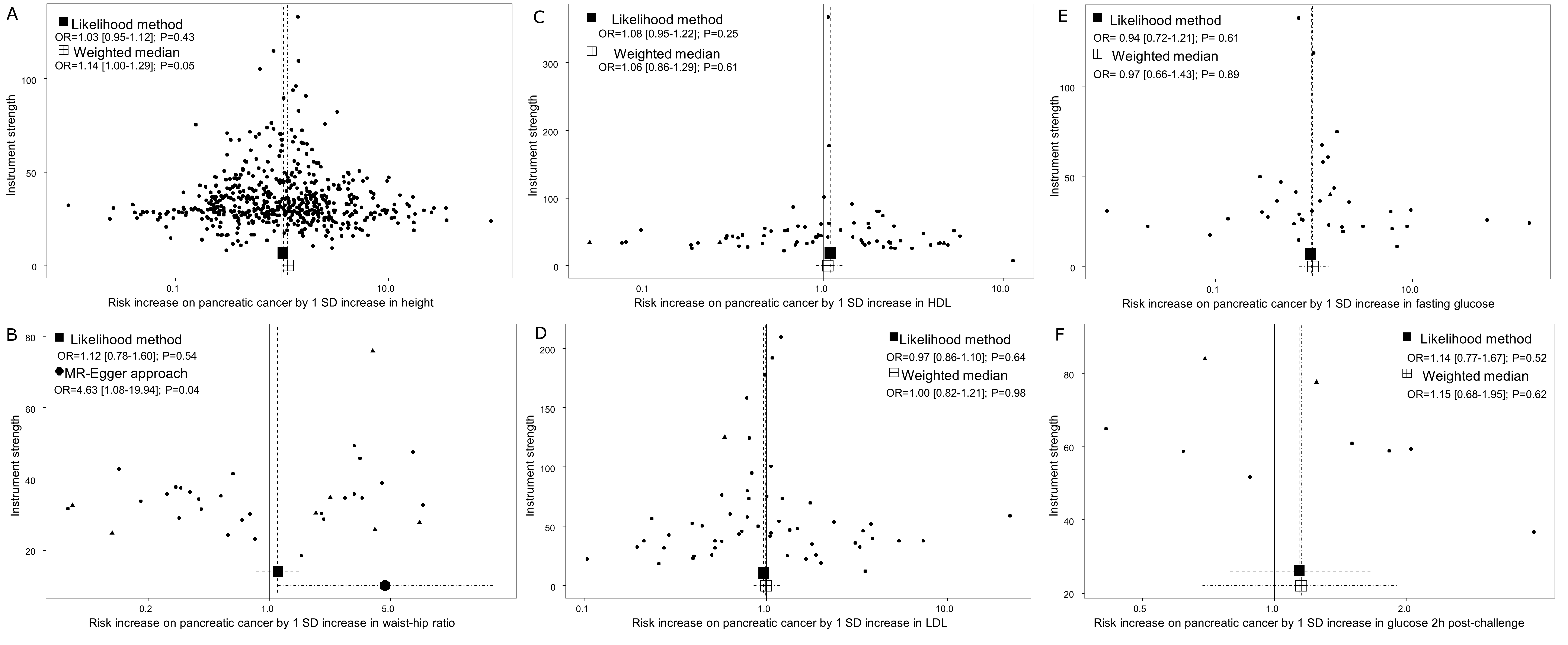


*Instrumental strength: SNP to pancreatic cancer effect corrected by SNP to exposure standard error of the effect. X-axes are in logarithmic scale. SNPs also robustly associated with BMI (3 SNPs for HDL, 2 for 2h post-challenge glucose, and 1 for LDL and fasting glucose) are depicted as triangles. SNP: Single nucleotide polymorphism. BMI: Body mass index. HDL: High-density lipoprotein cholesterol. LDL: Low-density lipoprotein cholesterol. OR: Odds ratio. SD: Standard deviation. P: Two-sided P value of Mendelian randomization test
